# Supplementary material for: Ultrathin covalent organic overlayers on metal nanocrystals for highly selective plasmonic photocatalysis
Source: Nat Commun. 2023 Nov 23;14:7667. doi: 10.1038/s41467-023-43482-x (PMC10667221; doi:10.1038/s41467-023-43482-x)
Supplement: Supplementary file 1 — Supplementary Information [file 41467_2023_43482_MOESM1_ESM.pdf]

Supplementary Information

Ultrathin Covalent Organic Overlayers on Metal Nanocrystals for Highly Selective Plasmonic Photocatalysis

Anubhab Acharya,[1,2] Trimbak Baliram Mete,[1,2] Nitee Kumari,[1,2] Youngkwan Yoon,[2] Hayoung Jeong,[3] Taehyung Jang,[4] Byeongju Song,[3] Hee Cheul Choi,[2] Jeong Woo Han,[3] Yoonsoo Pang,[4] Yongju Yun,[3] Amit Kumar,*[1,2] In Su Lee*[1,2,5]

[1]Creative Research Initiative Center for Nanospace-confined Chemical Reactions (NCCR) and Department of Chemistry, Pohang University of Science and Technology (POSTECH), Pohang 37673 (Korea)

[2]Department of Materials Science & Engineering, Pohang University of Science and Technology (POSTECH), Pohang 37673 (Korea)

[3]Department of Chemical Engineering, Pohang University of Science and Technology (POSTECH), Pohang 37673 (Korea)

[4]Department of Chemistry, Gwangju Institute of Science and Technology (GIST), Gwangju 61005 (Korea)

[5]Institute for Convergence Research and Education in Advanced Technology (I-CREATE), Yonsei University, Seoul 03722 (Korea)

*Correspondence: amitkumar@postech.ac.kr (A.K.); insulee97@postech.ac.kr (I.S.L.)

Table of Contents

1. Supplementary Information……….…………………………………………………………..Page-1

2. Supplementary Methods……………………………………………….……………...Page-2

3. Supplementary Figures………………………………………………………………. Page-14

4. Supplementary Synthesis Methods and Catalysis.……………...…………………………… Page-61

5. Supplementary ^{1}H NMR Spectra…………………………………………………………..Page-69

6. Supplementary Table………………………………………………………………… Page-80

7. Supplementary References…………………………………………………………... Page-81

1. Supplementary Information

1.1. Instruments and characterization.

Several chemical reactions were performed using different wavelength of lasers as mentioned here with specific model numbers, 405 nm: MDL-III-405nm-300mW-BL22446 (PO:EG1012918-CNI), 532 nm: MGL-III-532nm-300mW-BH80260 (PO:EG1012918-CNI), 808 nm: FC-W-808A-50W-17180 (PO:1127-0907-12C8R) Transmission electron microscopy (TEM) was conducted using JEOL (JEM-2100), JEM-ARM200F [coupled with a scanning transmission electron microscope (STEM)-energy dispersive spectrometry (EDS) elemental mapping module], and JEM-2100F (coupled with an STEM-EDS elemental mapping and line profiling module) instruments. Atomic scale HAADF-STEM analysis was performed using a 200 kV operated STEM (JEOL ARM200F) with a spherical aberration corrector (ASCOR, CEOS GmbH, Germany). Electron energy loss spectroscopy (EELS) elemental mapping was performed using JEM-2200FS at 200 kV. XPS measurements were performed on a PHI versa probe system, using a monochromatic Al Kα source. The contents of the metal elements in the nanoparticles were measured by inductive coupled plasma atomic emission spectrometry (ICP-AES) using iCAP 7400 (Thermo Scientific TM). UV−vis spectroscopy was carried out with a JASCO V-650 UV−vis spectrophotometer. Each chemical structure of the synthesized samples were investigated by Fourier transform infrared spectroscopy (FT-IR, Two IR spectrometer, PerkinElmer). Powder X-ray diffraction (XRD) patterns were recorded using a D/MAX-2500/PC (18 kW) (Rigaku) diffractometer with Cu-Kα radiation (λ = 0.15418 nm) at 40 kV and 100 mA. ^{1}H nuclear magnetic resonance (NMR) spectra were recorded on a Bruker 500 MHz (AVANCE III Ascend 500) in the deuterated solvents. Chemical shifts for proton and carbon spectra are reported on the δ scale in ppm. The Raman spectra were recorded using a WITECH Alpha 300 R Raman spectroscope equipped with a Nd:YAG laser (excitation wavelength: 532 nm). The full width at half maximum (FWHM) value of 2D-band was extracted from a 100 % Lorentzian profile of each Raman spectrum. For real-time Raman experiments, microscope slides (Citoglas; 1 mm-1.2 mm thick) with micro-holes were used. For Raman thermometry experiments, Raman spectra were obtained with a home-built micro-Raman setup.[1] An excitation laser beam, 632.8 nm in wavelength was focused on samples using an objective lens (40×, numerical aperture = 0.60). Backscattered Raman signals collected by the same objective lens were guided into a Czerny−Turner spectrometer (Princeton Instruments, SP2300) that was connected to a CCD (charge-coupled device camera, Princeton Instruments, PyLon). The spectral resolution defined by fwhm of a Rayleigh peak was 9.0 cm^{-1}, and the spectral accuracy was better than 1.0 cm^{-1}.

1.2. Reagents and materials

All chemicals were used without purification as commercially available unless otherwise noted. $HAuCl_4.6H_2O$ (Sigma-Aldrich), $Na_2PdCl_4·xH_2O$ (Strem), hexadecyltrimethylammonium chloride

(Sigma-Aldrich), silver nitrate (99.8 %; Samchun), tetraethyl orthosilicate (Acros), ethylene glycol (99.5 %; Samchun), 1,3,5-tris(4-aminophenyl)benzene (>93.0 %; TCI), 1,4-benzenedimethanol (99 %, Acros organics), terephthaldehyde (99 %; Sigma-Aldrich) 1,4-dioxane (99.5 % Samchun), mesitylene (99 %; Acros organics), methyl methacrylate (99 %; Sigma-Aldrich), diphenylacetylene (99 %; Strem), borane-ammonia complex (97 %; Sigma-Aldrich), 4-iodoanisole (98 %; Sigma-Aldrich), $Pd(PPh_3)_4$ (99 %; Sigma-Aldrich), Copper(I) iodide (98 %; Sigma-Aldrich), phenylacetylene (99 %; Sigma-Aldrich), Iodobenzene (98 %; Sigma-Aldrich), Triethylamine (99 %; Sigma-Aldrich), 4-Iodotoluene (99 %; Sigma-Aldrich), 4-Bromobenzaldehyde (99 %; Sigma-Aldrich), Sodium borohydride (98 %; Sigma-Aldrich), Phosphorus tribromide (99 %; Sigma-Aldrich), Thioacetic acid (96 %; Sigma-Aldrich), 4-Ethynylanisole (97 %; Sigma-Aldrich), 4-tert-Butylphenylacetylene (96 %; Sigma-Aldrich), 3-Ethynyltoluene (97 %; Sigma-Aldrich), 1-Octyne (97 %; Sigma-Aldrich), Propargyl alcohol (99 %; Sigma-Aldrich), 1-Ethynylpyrene (97 %; Sigma-Aldrich) (97 %; Sigma-Aldrich) (97 %; Sigma-Aldrich)

2. Supplementary Methods

2.1. Synthesis of silver nanocubes (AgNCs). The AgNCs with high dispersity and homogeneity were synthesized via a modified method reported by S. Singamaneni.[2] At first, 2 mL of hexadecyltrimethylammonium chloride (CTAC; 100 mM), 5 µL sodium hydroxide (NaOH; 0.5 N) and 1 mL ascorbic acid (AA; 10 mM) were mixed together in a 20 mL vial and kept this in stirring to prepare the reduction solution (A). In another vial, a template solution (B) was prepared by mixing 0.5 mL silver nitrate ($AgNO_3$; 10 mM) and seed solution of 50 µL Au-nanorods (2 mM in hexadecyltrimethylammonium bromide; CTAB) under stirring inside an incubator (60 °C) followed by quick addition of 0.5 mL silver nitrate ($AgNO_3$; 10 mM) solution. After few minutes, the template solution (B) was poured into the reduction solution (A) at 60 °C and kept in gentle stirring for 90 min. Then, the mixture was placed at room temperature undisturbed for 24 h. The product was centrifuged (10,000 rpm, 10 min) and purified with CTAB (2 mM) for further use.

2.2. Synthesis of AgNC encapsulated inside hollow silica (AgNC@*h*-SiO_2). Stepwise silica (SiO_2) coating on AgNC and hollowing of SiO_2-shell were executed by following the reported method.[3] For SiO_2-coating, 10 mL of Ag-NCs were centrifuged to remove the excess surfactant molecules and further dissolved in a 15 mL CTAB (1 mM) solution. To adjust the pH value (~10.5), NaOH (50 µL; 0.1 N) solution was injected quickly and stirred for the next few minutes. A diluted solution (20% in anhy. MeOH) of tetraethyl orthosilicate (TEOS; 100 µL) was used as a precursor for SiO_2-coating adapted by a modified Stöber method. Then the TEOS solution was injected slowly (10 µL/min) under vigorous stirring at 30 °C for 30 min. After that, the solution was kept incubated overnight in unstirred condition for the complete growth of SiO_2–shell on AgNC, designated as, AgNC@SiO_2. The final product was centrifuged (10,000 rpm, 10 min) and washed with DI-water (2 mL) for further use. Then, the hollowing of SiO_2–shell was performed by hot water treatment to etch out the fragile SiO_2 network in the inner

core of the SiO_2-shell. For that, as prepared AgNC@SiO_2 nanoparticles are dissolved in 20 mL of DI-water and placed in an oil bath at 80 °C for 30 min with vigorous stirring. After completing the hydrolysis process, the product was centrifuged (10,000 rpm, 10 min) and washed with DI-water. For further use, the final product (AgNC@*h*-SiO_2) was redispersed in 2 mL DI-water and stored at 4 °C.

2.3. Synthesis of AuNC@*h*-SiO_2. At first, homogeneous AuNCs were synthesized by following a reported method reported by Nam et al.[3] 5 ml of AuNCs were centrifuged to remove the excess surfactant molecules and further dissolved in a 15 mL CTAB (1 mM) solution. To adjust the pH value (~10.5), NaOH (50 µL; 0.1 N) solution was injected quickly and stirred for the next few minutes. A diluted solution (20% in anhy. MeOH) of tetraethyl orthosilicate (TEOS; 100 µL) was used as a precursor for SiO_2-coating adapted by a modified stöber method. Then the TEOS solution was injected slowly (10 µL/min) under vigorous stirring at 30 °C for 30 min. After that, the solution was kept incubated overnight in unstirred condition for the complete growth of SiO_2–shell on AuNC, designated as, AuNC@SiO_2. The final product was centrifuged (10,000 rpm, 10 min) and washed with DI-water (2 mL) for further use. Then, similarly the hollowing of SiO_2–shell was performed by hot water treatment. For that, as prepared AuNC@SiO_2 nanoparticles are dissolved in 20 mL of DI-water and placed in an oil bath at 80 °C for 30 min with vigorous stirring. After completing the hydrolysis process, the product was centrifuged (10,000 rpm, 10 min) and washed with DI-water. For further use, the final product (AuNC@*h*-SiO_2) was redispersed in 2 mL DI-water and stored at 4 °C.

Experimental setup-1 to synthesize pCOL on AgNC@*h*-SiO_2

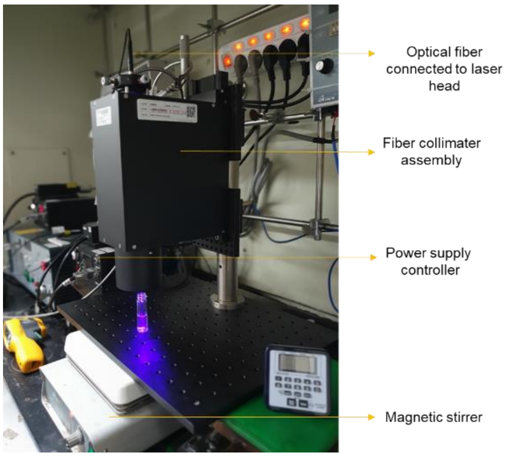

2.4. Synthesis of control catalyst Pd/AuNC@*h*-SiO_2. AuNC@*h*-SiO_2 was mixed with 1 mL of 1,2-ethanedithiol (H_2O/ethanol; 2/3) and kept overnight with stirring at room temperature. Then, the solution was washed with H_2O/ethanol (2/3) solution and dispersed completely in ethylene glycol through sonication. The whole solution was then transferred into an 8 mL glass vial, followed by quick addition of 20 µL Na_2PdCl_4 solution (5 mM in ethylene glycol). Then the vial was placed under the exposure of 532 nm laser (0.3 W/cm^2) with constant stirring for 30 min. The final product was

centrifuged, washed with ethanol (2 mL) and redispersed in ethanol.

2.5. Evidence for photochemical oxidation of 1,4-benzenedimethanol (DAL) on AgNC@*h*-SiO_2. A mixture of 1,4-benzenedimethanol (DAL) (0.072 mmol) and AgNC@*h*-SiO_2 (500 µL 5 gm/mL) was taken in a glass vial and dispersed well under sonication for 2 min.. After that, whole solution was irradiated under 405 nm laser (0.3 W/cm^2) for 5 h under constant stirring using a small (10 x 3 mm) magnetic stirring bar. After that, nanoparticles were removed by centrifugation (10,000 rpm) and solution was concentrated under reduced pressure. Finally, the crude product was analyzed by ^{1}H NMR.

2.6. Attempt to synthesize pCOL by direct condensation of aldehyde and amine on AgNC@*h*-SiO_2. First of all, 8.8 mg of 1,3,5-tris(4-aminophenyl)benzene (TAE) (0.025 mmol) and 7 mg of terephthaldehyde (DAE) (0.051 mmol) were dissolved in a 5 mL solution of 1,4-dioxane/mesitylene (4/1, v/v) under sonication and transferred into a 8 mL glass vial. In another vial, 5 mg of AgNC@*h*-SiO_2 was also completely dispersed in 1 mL of 1,4-dioxane/mesitylene (4/1, v/v) under sonication and injected the solution quickly into the first vial under vigorous stirring using small (10 x 3 mm) magnetic stirring bar. In next step, the whole solution was placed under a blue laser (405 nm; 0.3 W/cm^2) for 1 h with constant stirring at 500 rpm. The product was collected and centrifuged and washed with 1,4-dioxane/mesitylene (4/1, v/v) and anhy. ethanol (99.9 %). Finally, the product was redispersed in ethanol and prepared for TEM analysis.

2.7. Attempt to synthesize pCOL by direct condensation of aldehyde and amine on CTAB-AgNCs. In a 20 mL glass vial, 10.5 mg of 1,3,5- tris(4-aminophenyl)benzene (0.03 mmol) and 8.7 mg of terephthaldehyde (0.045 mmol) were dissolved in 4 mL of 1,4-dioxane:butanol (v/v, 1:1) solution. Then, 1 mL of as prepared AgNC (CTAB)-solution was added into the mixture followed by addition of 0.05 mL aqueous acetic acid (12 M) and placed the whole solution in an oil bath (70 °C) for 24 h with constant stirring. After cooling to room temperature, the product was collected by centrifugation, washed with 1,4-dioxane:butanol (1 mL) and acetone (1 mL). Finally the product was vacuum dried at room temperature overnight and prepared for further analysis.

2.8. Attempt to synthesize pCOL on ligand-deficient AgNCs using laser. At first, 8.8 mg of TAE (0.025 mmol) and 7 mg of DAL (0.051 mmol) were dissolved in a 5 mL solution of 1,4-dioxane/mesitylene (4/1, v/v) under sonication and transferred the solution into a 8 mL glass vial. Then, 1 mL AgNC (CTAB stabilized) solution (5 mg/mL) was centrifuged (2x) to remove surfactants from the surface and dispersed in 1 mL of 1,4-dioxane/mesitylene (4/1, v/v) under sonication. This process was leading to a change in colour of the solution from pale yellow to dusky colour indicating the etching of surfactants and quick aggregation of AgNCs in 1,4-dioxane/mesitylene (4/1, v/v) solution. Immediately, the AgNCs solution was transferred into the first vial under vigorous stirring. Then, the whole solution was placed under a 405 nm laser (0.3 W/cm^2) for 1 h with constant stirring at 500 rpm.

The product was collected, centrifuged and washed with 1,4-dioxane/mesitylene (4/1, v/v) and anhy. ethanol. Finally, the product was redispersed in ethanol and prepared for TEM analysis.

2.9. Synthesis of control catalyst having bulk COF encapsulating Pd/AuNCs. In the first step, Pd/AuNC was synthesized by taking a dispersive solution of AuNC (500 µL, 5 mg/mL) in ethylene glycol in a glass vial. Then, 20 µL Na_2PdCl_4 solution (5 mM in ethylene glycol) solution was quickly injected into the solution and placed under 532 nm laser (0.3 W/cm^2) for 30 min with continuous stirring. Then the as synthesized Pd/AuNC was centrifuged and washed with water (2 mL) two times and used in next step. After that, in a 20 mL glass vial, 10.5 mg of 1,3,5- tris(4-aminophenyl)benzene (0.03 mmol) and 8.7 mg of terephthaldehyde (0.045 mmol) were dissolved in 4 mL of 1,4-dioxane:butanol (v/v, 1:1) solution. Then, 1 mL of as prepared Pd/AuNC was added into the mixture followed by addition of 0.05 mL aqueous acetic acid (12 M) and placed the whole solution in an oil bath (70 °C) for 24 h with constant stirring. After cooling to room temperature, the product was collected by centrifugation, washed with 1,4-dioxane:butanol (1 mL) and acetone (1 mL). Finally the product was vacuum dried at room temperature overnight and prepared for further analysis.

2.10. Attempt to synthesize pCOL on AgNC@*h*-SiO_2 by external heating. At first, 8.8 mg of TAE (0.025 mmol) and 7 mg of DAE (0.05 mmol) were dissolved in a 5 mL solution of 1,4-dioxane/mesitylene (4/1, v/v) under sonication and transferred the solution into a 8 mL glass vial. In another vial, 5 mg of AgNC@*h*-SiO_2 was also completely dispersed in 1 mL of 1,4-dioxane/mesitylene (4/1, v/v) under sonication and injected quickly into the first vial under vigorous stirring. Then, the whole solution was placed into a pre-heated (100 °C) oil bath and kept on constant stirring at 500 rpm for next 1 h. The product was centrifuged and washed with 1,4-dioxane/mesitylene (4/1, v/v) and anhy. ethanol. Finally, the product was redispersed in ethanol and prepared for TEM analysis.

2.11. Attempt to synthesize pCOL on AgNC@*h*-SiO_2 under non-resonant laser (808 nm) irradiation: At first, 8.8 mg of TAE (0.025 mmol) and 7 mg of DAL (0.05 mmol) were dissolved in a 5 mL solution of 1,4-dioxane/mesitylene (4/1, v/v) under sonication and transferred the solution into a 8 mL glas vial. In another vial, 5 mg of AgNC@*h*-SiO_2 was also completely dispersed in 1 mL of 1,4-dioxane/mesitylene (4/1, v/v) under sonication and injected the solution quickly into the first vial under vigorous stirring. Then, the whole solution was placed under a 808 nm laser (0.4 W/cm^2) and kept on stirring at 500 rpm for 1 h. The product was centrifuged and washed with 1,4-dioxane/mesitylene (4/1, v/v) and anhy. ethanol (99.9 %). Finally, the product was redispersed in ethanol and prepared for TEM analysis.

2.12. Attempt to synthesize pCOL on AgNC@*h*-SiO_2 under deoxygenated environment: To carry out this controlled experiment under deoxygenated condition, 8.8 mg of TAE (0.025 mmol) and 7 mg of DAL (0.05 mmol) were dissolved in a 5 mL solution of 1,4-dioxane/mesitylene (4/1, v/v) with

AgNC@*h*-SiO_2 under sonication. Then, the solution was constantly purged with N_2 gas for 15 min and immediately transferred into a sealed glass vial having a single outlet closed with rubber septum attached with a N_2 gas balloon (as shown in the **Experimental setup 2**). The whole set-up was placed under 405 nm laser (0.3 W/cm^2) and the solution was constantly stirred for next 1 h. The final product was centrifuged and washed stepwise with 1,4-dioxane/mesitylene (4/1, v/v) and anhy. ethanol. Finally, the product was redispersed in ethanol and prepared for TEM analysis.

Experimental setup-2

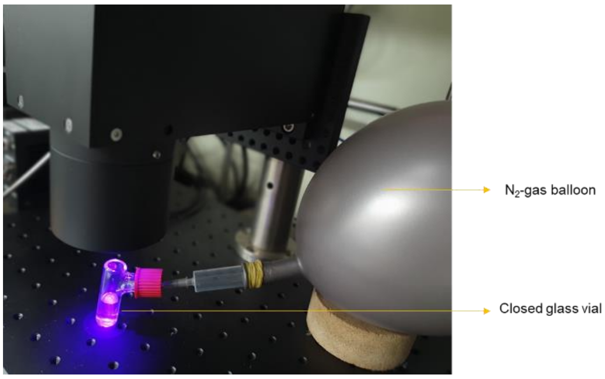

2.13. Synthesis of control catalyst TAE-Pd/AuNC@*h*-SiO_2. At first, 8.8 mg of TAE (0.025 mmol) was dissolved in a 5 mL solution of 1,4-dioxane/mesitylene (4/1, v/v) under sonication and transferred the solution into a 8 mL glass vial. In another vial, 5 mg of AgNC@*h*-SiO_2 was also completely dispersed in 1 mL of 1,4-dioxane/mesitylene (4/1, v/v) under sonication and injected quickly into the first vial under vigorous stirring using small (10 x 3 mm) magnetic stirring bar. Then, the whole solution was placed under 405 nm (0.3 W/cm^2) for 1 h with constant stirring at 500 rpm. After completing the reaction, the product, TAE-AuNC@*h*-SiO_2 was centrifuged and washed with 1,4-dioxane/mesitylene (4/1, v/v) and anhy. ethanol (99.9 %). In next step, the as synthesized TAE-AuNC@*h*-SiO_2 nanoparticles were dispersed in 5 mL of ethylene glycol and placed under 532 nm laser (0.3 W/cm^2), followed by a quick injection of 20 µL Na_2PdCl_4 solution (5 mM in ethylene glycol). The whole solution was continuously stirred with 500 rpm for 30 min under laser and then centrifuged and washed with ethanol (2 mL, 2x).

2.14. Photochemical polymerization of methyl methacrylate on AgNC@*h*-SiO_2. At first, 100 µL of methyl methacrylate was dissolved in 2-propanol (0.5 mL) and poured into an 8 mL glass vial. Then, as prepared 0.5 mL AgNC@*h*-SiO_2 (5 mg/mL) dissolved in 2-propanol added into the mixture and placed under the 405 nm laser (0.3 W/cm^2) with constant stirring (500 rpm) using a small (10 x 3 mm) magnetic stirring bar for 1 h. The product was centrifuged, washed and dispersed in 2-propanol (1 mL). Another controlled experiment was performed at inert atmosphere in a perfectly sealed glass vial attached with N_2 gas balloon.

2.15. Synthesis of thicker pCOL on AuNP coated on SiO_2 (pCOL-Pd/AuNP@SiO_2).

Step 1: Synthesis of SiO_2 NPs: Aqueous ammonia (28–30%, 200 μL) was added into a mixture of IGEPAL 520 (1.2 mL) and IGEPAL 720 (1.2 mL) in cyclohexane (20 mL) under vigorous magnetic stirring. Then the solution of TEOS (4.51 M, 100 μL) and TESD (3.56 M, 150 μL) was injected twice at first, followed by five times addition of TMSD (4.62 M, 20 μL) into the suspension at every half-hour interval, consecutively. Then, After 24 h, TEOS (4.51 M, 100 μL) was injected to the solution, followed by constant stirring at 25 °C for 48 h. The resulting white colored SiO_2 NPs were collected by centrifugation and washed with ethanol (three times) and deionized (DI) water (one time).

Step 2: Synthesis of Au-shell around SiO_2 NPs: An aqueous solution of $HAuCl_4$ (1 mL, 15 mM) was mixed with the aqueous suspension of SiO_2 (300 μL, 5 mg/mL) and vortexed for 2 h at room temperature. Then, the mixture centrifuged (10000 rpm, 1 min) and washed off the excess $HAuCl_4$ with DI water (1 mL, 2 times). A solution of $NaBH_4$ (200 μL, 100 mM) was rapidly injected. The brown coloured solution of Au-seed@SiO_2 was centrifuged again and washed with DI-water. In next step, the aqueous suspension of Au-seed@SiO_2 was mixed with iron(III) chloride (50 μL, 10 mM) and tannic acid (50 μL, 10 mM) followed by addition of NaOH (50 μL, 1 M) and vortex for 30 s and washed with DI-water. Finally, the product from previous steps were mixed in 2% PVP solution (200 μL) followed by rapid injection of $HAuCl_4$ (200 μL, 5 mM) and hydroquinone (200 μL, 100 mM), resulting an immediate bluish brown coloured solution and left for vortex till 15 min at R.T. The final product of AuNP@SiO_2 was washed and dispersed in DI-water and stored in refrigerator for further use.

Step 3: Deposition of Pd: As prepared aqueous suspension AuNP@SiO_2 in step 2, was centrifuged to remove the DI-water completely and dispersed again in ethylene glycol followed by sonication for complete dispersion. Then, a solution of Na_2PdCl_4 (50 μL, 5 mM) in ethylene glycol was quickly injected and placed under 808 nm laser (0.4 W/cm^2) with constant stirring for 30 min. The product was washed with DI-water (1 mL, 2 times) and stored in refrigerator for further use.

Step 4: Formation of pCOL: To synthesize pCOL layer on plasmonically active Pd/AuNP@SiO_2 (from step 2) at first, TAE (0.01 mmol) and DAE (0.2 mmol) were dissolved in a 5 mL solution of 1,4-dioxane/mesitylene (4/1, v/v) under sonication and transferred into a 8 mL glass vial. In another vial, Pd/Au@SiO_2 (5 mg) was completely dispersed in 1 mL of 1,4-dioxane/mesitylene (4/1, v/v) under sonication and injected quickly into the first vial and sonicated for complete dispersion. Next, the whole solution was placed under a NIR laser (808 nm; 0.4 W/cm^2) for 1.5 h with constant stirring at 500 rpm. After completing the reaction, the product was centrifuged and washed with 1,4-dioxane/mesitylene (4/1, v/v) and anhy. ethanol (99.9 %). After washing, the final product (pCOL-Pd/AuNP@SiO_2) was used for XRD and BET characterizations.

2.16. Synthesis of control catalyst having PdNPs on SiO_2 encapsulated inside pCOL (pCOL-

PdNP@SiO_2**).**

Step 1: Synthesis of PdNPs on SiO_2: An aqueous suspension of SiO_2 (300 µL, 5 mg/mL) was mixed with Na_2PdCl_4 (1 mL, 15 mM) and vortexed for 2 h at 25 °C and washed off the excess Na_2PdCl_4 with DI water (1 mL, 2 times). A solution of $NaBH_4$ (200 µL, 100 mM) was rapidly injected. Further, the aqueous suspension of Pd-seed@SiO_2 was mixed with iron(III) chloride (50 µL, 10 mM) and tannic acid (50 µL, 10 mM) followed by addition of NaOH (50 µL, 1 M) and vortex for 30 s and washed with DI-water. Finally, the product from previous steps were mixed in 2% PVP solution (200 µL) followed by rapid injection of Na_2PdCl_4 (200 µL, 5 mM) and hydroquinone (200 µL, 100 mM), resulting an immediate black coloured solution and left for vortex till 15 min at R.T. The final product of PdNPs@SiO_2 was washed and dispersed in DI-water and stored in refrigerator for further use.

Step 2: Formation of pCOL: To prepare pCOL-PdNP@SiO_2, the following synthesis method was carried out. In a 20 mL glass vial, 10.5 mg of TAE (0.03 mmol) and DAE (0.045 mmol) were dissolved in 4 mL of 1,4-dioxane:butanol (v/v, 1:1) solution. Then, 1 mL of as prepared PdNPs@SiO_2 (1 mg/mL in 1,4-dioxane:butanol) solution was added into the mixture followed by addition of aqueous acetic acid (0.05 mL, 12 M) and placed the whole solution in an oil bath (70 °C) for 24 h with constant stirring. After cooling to room temperature, the product was collected by centrifugation, washed with 1,4-dioxane:butanol (1 mL) and acetone (1 mL). Finally the product was vacuum dried at room temperature overnight and prepared for further analysis.

2.17. Semihydrogenation catalysis under dark-condition at 30 °C. At first, in a glass vial 10 mg diphenylacetylene (0.056 mmol, 1 equiv.) was dissolved in 1 mL of methanol. Then, the catalyst pCOL-Pd/AuNC@*h*-SiO_2 (Pd 0.05 mol %) was transferred into that solution and dispersed fully with stirring for 5 min. Then, 8.6 mg $NH_3.BH_3$ (0.280 mmol, 5 equiv.) was added into the reaction mixture. Immediately, the glass vial was completely wrapped with Al-foil and placed in an oil bath (30°C) and kept on stirring for >24 h. During the reaction, all lights of the fume hood was turned off to maintain the dark environment. Then, the catalyst was removed by centrifugation (10,000 rpm) and the solution was concentrated under reduced pressure. After that, DI-water (1 ml) was added and the product was extracted in ethyl acetate (1 mL, 2x), combined ethyl acetate layers, dried over sodium sulfate, concentrated under reduced pressure and recorded the ^1H NMR spectra.

2.18. Semihydrogenation catalysis under dark and external heating condition. In a glass vial 10 mg diphenylacetylene (0.056 mmol, 1 equiv.) was dissolved in 1 mL of methanol. Then, pCOL-Pd/AuNC@*h*-SiO_2 (Pd 0.05 mol %) catalyst was transferred into that solution and dispersed fully with stirring for 5 min. Then, 8.6 mg $NH_3.BH_3$ (0.280 mmol, 5 equiv.) was added into the reaction mixture and the whole solution was placed in oil bath (connected to a temperature controller) to perform the reactions at different temperatures (such as, 30, 40, 50, 60 and 80 °C) with constant stirring (500 rpm)

for 1 h using a small (10 x 3 mm) magnetic stirring bar . Only, in case of the reaction at 80 °C, 2-propanol was used as solvent due to the lower boiling point of methanol (<65 °C). During the reaction, all lights of the fume hood was turned off. After the reaction, catalyst was removed by centrifugation (10,000 rpm) and the solution was concentrated under reduced pressure. After that, DI-water (1 ml) was added and the product was extracted in ethyl acetate (1 mL, 2x), combined ethyl acetate layers, dried over sodium sulfate, concentrated under reduced pressure and recorded the ^{1}H NMR spectra.

2.19. Semihydrogenation catalysis under external heating during laser irradiation. 10 mg diphenylacetylene (0.056 mmol, 1 equiv.) was dissolved in 1 mL of methanol. Then, pCOL-Pd/AuNC@*h*-SiO_2 (Pd 0.05 mol %) catalyst was transferred into that solution and dispersed fully with stirring for 5 min. Next, 8.6 mg NH_3BH_3 (0.280 mmol, 5 equiv.) was added into the reaction mixture and the whole solution was irradiated under 405 nm laser (0.3 W/cm^2) with constant stirring (500 rpm) using a small (10 x 3 mm) magnetic stirring bar. After 30 min of continuous laser irradiation, the glass vial was quickly dipped into a preheated oil-bath (60 °C) attached under the laser set-up and stirred for next 15 min while continuing the laser irradiation simultaneously from the top. After completing the reaction, catalyst was removed by centrifugation (10,000 rpm) and the solution was concentrated under reduced pressure. Then DI-water (1 ml) was added and the product was extracted in ethyl acetate (1 mL, 2x), combined ethyl acetate layers, dried over sodium sulfate, concentrated under reduced pressure and recorded the ^{1}H NMR spectra.

2.20. Real-time SERS study using thiolated-diphenylacetylene modified pCOL-Pd/AuNC@*h*-SiO_2. The as synthesized thiolated-diphenylacetylene (described in the section-4) was tethered on NP surface through chemical reaction. For that, 0.5 mL of concentrated methanol solution of thiolated-diphenylacetylene (100 mM) was mixed with 0.5 mL of pCOL-Pd/AuNC@*h*-SiO_2 (5 mg/mL) via sonication and placed into a shaker (500 rpm) for 2 h. The product was washed with absolute methanol (1ml; 2x) and used for real time SERS study.

Diphenylacetylene-modified pCOL-Pd/AuNC@*h*-SiO_2 (Pd 0.05 mol %) was completely dispersed in 1 ml methanol solution. Then, 8.6 mg $NH_3.BH_3$ (0.280 mmol, 5 equiv.) was quickly added into the solution and mixed thoroughly. Immediately, the reaction mixture was pipetted out and poured into the micro-holes of the microscopic glass slide and fully covered the hole with an ultrathin round glass slide. Then, it was placed under the 532 nm laser attached with Raman spectrometer and the corresponding time dependent spectra were recorded (as shown in **Experimental setup 3**).

Experimental setup-3

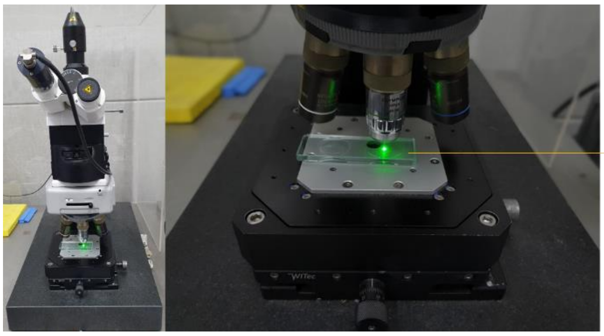

2.21. Real-time SERS study for hydrogen desorption. In two separate experiments, pCOL-Pd/AuNC@*h*-SiO_2 (0.5 mL, 3 mg/mL) and Pd/AuNC@*h*-SiO_2 (0.5 mL, 3 mg/mL) were completely dispersed in methanol via sonication. Then, 200 μL $NH_3.BH_3$ (200 mM) solution in methanol was quickly added into that mixture and shake well. Immediately after that, solution was pipetted out and poured into the micro-holes of the microscopic glass slide and fully covered the hole with an ultrathin round glass slide and the real time SERS signals were recorded for each sample under 532 nm laser attached with Raman spectrometer.

2.22. Sample preparation and method for Raman thermometry. In Raman thermometry experiment, 4-Aminothiophenol was chosen as Raman reporter molecule. Firstly, 5 mM concentration of 4-Aminothiophenol was dissolved in ethanol (100 μL) and then mixed with the catalyst pCOL-Pd/AuNC@*h*-SiO_2 (100 μL, 3 mg/mL in ethanol). The mixture was sonicated for few seconds followed by shaking (500 rpm) at room temperature for 5 h. After that, sample solution was dropped onto a glass slide and dried completely at room temperature.

In quantum mechanical description of Raman scattering, the ratio between the Stokes and anti-Stokes intensity for a given Raman mode can be expressed as follows,[4]

$$\frac{I_{aS}}{I_S} = \left(\frac{\omega + \omega_k}{\omega - \omega_k}\right)^4 \exp\left(-\frac{\hbar\omega_k}{k_B T}\right) = Cexp\left(-\frac{\hbar\omega_k}{k_B T}\right)$$

Where ω is the angular frequency of the excitation beam, $\hbar\omega_k$ is the energy of the Raman mode, k_B is Boltzmann constant, and T is the temperature.

Therefore, the temperature can be determined by the following equation.

$$T = -\frac{\hbar\omega_k}{k_B \ln\left(\frac{I_{as}}{I_s C}\right)}$$

To minimize the sample inhomogeneity, more than three measurements were performed for each laser intensity, and one of the calculated results is shown in the table below-

Power	I_{as}	I_s	I_{as}/I_s	ω_k	C	Temp. (K)	Temp. (°C)
0.3 mW	2197	203564	0.01079	2..031*10^{14}	1.727	305.6	32.4

2.23. ^{1}H NMR based isotope labeling experiments: $NH_3.BD_3$ and $ND_3.BH_3$ were synthesized according to the previously reported literatures.[5,6]

2.23.1. Synthesis of $NH_3.BD_3$. Under the Ar atmosphere, 2 mmol of $NaBD_4$ and 3 mmol of $(NH_4)_2SO_4$ were added to a 100 mL three-neck round bottom flask with a stirrer, a stopper, and a condenser with a connecting tube. The connecting tube was vented via an oil bubbler to the hood exhaust. 20 ml THF was added into the flask via a dropping funnel, and the reaction was stirred at 40 ºC for 3 h. After that, the reaction mixture was cooled to room temperature and filtered. The residue was washed with THF (10 mL). The filtrate was concentrated under reduced pressure to obtain $NH_3.BD_3$ as a white solid. Yield = 60%.

2.23.2. Synthesis of $ND_3.BH_3$. An oven-dried Schlenk flask was charged with $NH_3.BH_3$ (30.8 mg, 1 mmol) under an Ar atmosphere, and then 1 mL of D_2O was added to it. The mixture was stirred until $NH_3.BH_3$ was completely dissolved in D_2O to furnish a colorless solution. After that, the solvent was distilled under vacuum, resulting in a white crystalline solid. This process was repeated three times to get the $ND_3.BH_3$ with 99% incorporation of deuterium.

2.23.3. Kinetic isotope effect. The rate of the reaction was determined according to the initial rate law method. ^{1}H NMR was used to collect multiple data points up to 1 h. The least-square fit method determined the reaction rate with the product yield versus the time graph.

In a glass vial 10 mg diphenyl acetylene (0.056 mmol, 1 equiv.) was dissolved in of methanol. Then, as synthesized pCOL-Pd/AuNC@*h*-SiO_2 (Pd 0.05 mol %) catalyst was transferred into that solution and dispersed fully with stirring for 5 min. After that, 8.6 mg borane-ammonia complex (NH_3BH_3; 0.280 mmol, 5 equiv.) was added into the reaction mixture, and the whole solution was irradiated under 405 nm laser (0.3 W/cm^2) with constant stirring for 1 h. the aliquots were collected at every 10 min intervals and the yields were estimated by ^{1}H NMR. The initial rate was determined from the plot of the yield of alkene (cis-stilbene) vs. time. The same procedure was followed for NH_3BD_3, CD_3OD and ND_3BH_3.

2.24. GC-MS analysis. The reaction kinetics for semihydrogenation of diphenylacetylene was carried out by taking aliquots at different reaction times for GC analysis (YL6500) with a CP7502-Chirasil column (25 m × 0.25 mm × 0.25 µm). All response factor were calculated following the literature procedure. For this purpose, mesitylene was used as an internal standard and stock solutions of the corresponding components at different concentrations were prepared and analyzed by GCMS. Firstly,

the response factor (R_f) of each component was determined following the equation below-

(Area of analyte) / (Conc. of analyte) = R_f (Area of internal standard) / (Conc. of internal standard)

Now, we can calculate the concentration of each analyte using the response factor with respect to the same internal standard. For a reaction type, $A \rightarrow B$

- % GC yield of B = [Conc. of B / (Conc. of A + Conc. of B)] x 100

2.25. Transient absorption (TA) measurements. A home-built femtosecond TA setup based on 1 kHz Ti:sapphire regenerative amplifier (50 fs, 803 nm, Libra-USP-HE, Coherent) was utilized for the transient absorption measurements.[7] The actinic pulses at 403 and 550 nm were generated by sum-harmonic generation in a BBO crystal (θ = 29.2°, 100 μm thick) and by an optical parametric amplifier (TOPAS-Prime, Light-Conversion). The super-continuum probe pulses were generated in a 3 mm thick sapphire crystal, and the transient spectra in 420-750 nm range were obtained with a <50 fs temporal resolution. The polarization of the actinic pulses were set at a magic angle (54.7°) to that of the probe pulse not to include the reorientational dynamics of the chromophores in solutions. The samples were recirculated during the measurements to minimize the photodamage from the actinic pulses.

2.26. Density-functional theory (DFT) calculations. All DFT calculations were performed using the Vienna ab initio simulation package (VASP). The exchange-correlation effects were considered using the Perdew-Burke-Ernzerhof functional and projector augmented-wave pseudopotentials were used. The kinetic energy cutoff was set to be 400 eV for the expansion of plane wave. All atoms were relaxed until the force acting on each atom was less than 0.03 eV·Å^{-1}. The van der Waals interactions were considered using the DFT-D3 method of Grimme with zero-damping function. A Brillouin zone was sampled using 1x1x1 Monkhorst-Pack k-point grids for SU-pCOL@Pd slab [a single unit of pCOL (SU-pCOL) was considered as a simplified model] model calculations and all gas molecules. Pd (111) slab model consists of three layers and bottom two layers were fixed. The slabs were separated by vacuum layer more than 10 Å. The gas-phase molecules were calculated in a cubic unit cell with each side of 15 Å. The converge criterion of energy was 1 x 10^{-5} eV for the self-consistent-field (SCF) iterations. The adsorption energy (E_{ads}) was calculated as follows;

$$E_{ads}=E_{mol/slab} - E_{slab} - E_{mol}$$

Where, $E_{mol/slab}$ corresponds to the total energy of the molecule-adsorbed slab, E_{slab} corresponds to the total energy of the slab model, and E_{mol} corresponds to the total energy of the gas-phase molecule.

Bader charge analysis was performed on a Pd (111) slab and a SU-pCOL@Pd slab. The net charge was calculated as the difference in before and after the SU-pCOL was adsorbed on Pd surface. The atom color indicating relative comparison based on the maximum change in atomic charge.

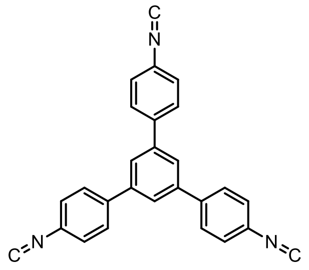

SU-pCOL

3. Supplementary Figures

a

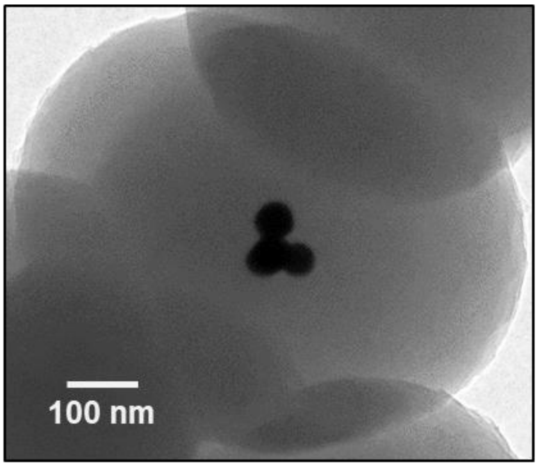

b

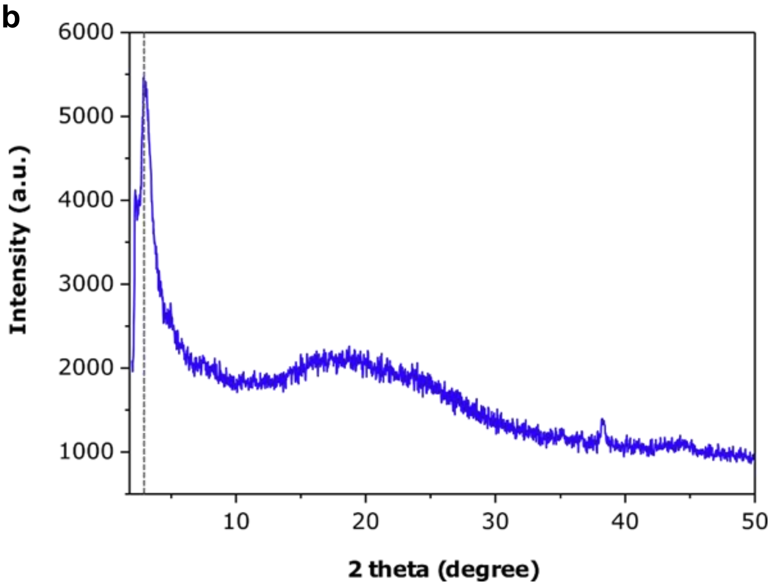

Supplementary Figure 1. Synthesis of bulk COF-shell on AgNCs. (a) TEM image of thick COF layer on aggregated AgNCs through condensation-crosslinking-assembly of DAE and TAE. (b) Powder-XRD data verifying the presence of thick COF-shell on AgNCs.

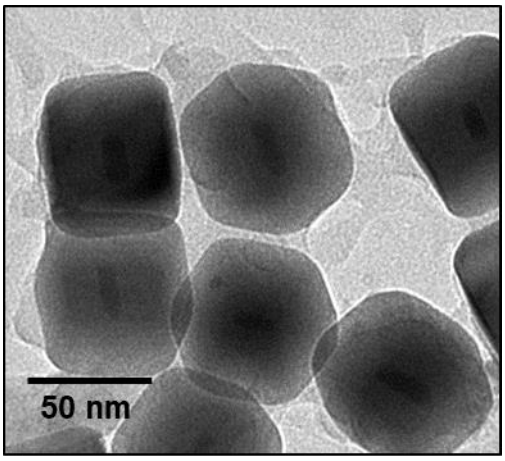

Supplementary Figure 2. COF-shell on aggregated AgNCs. TEM image of AgNCs showing aggregation under ligand-deficient condition upon formation of bulk COF.

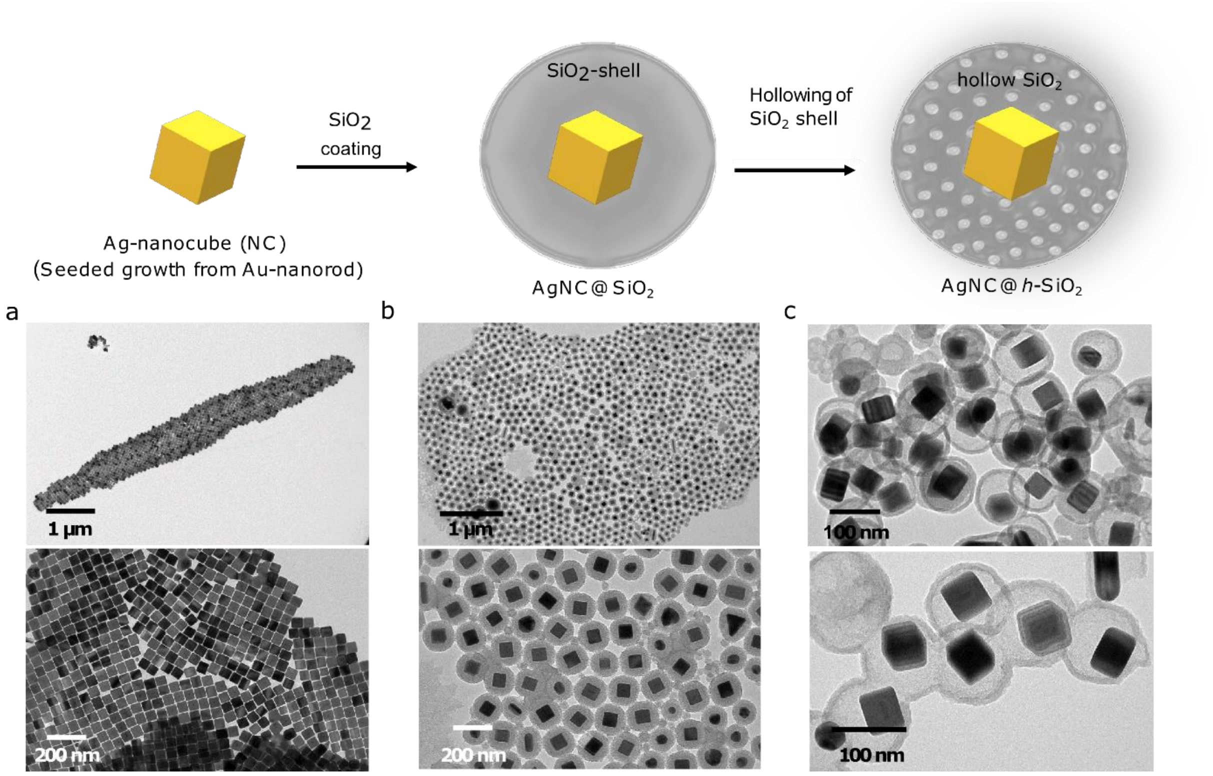

Supplementary Figure 3. Step-wise synthesis of ligand-free plasmonic AgNC inside *h*-SiO_2 (AgNC@*h*-SiO_2). Low and high-mag TEM images of (a) AgNCs, (b) SiO_2 coating on AgNCs (AgNC@SiO_2), and (c) AgNC inside hollowed SiO_2-shell (AgNC@*h*-SiO_2).

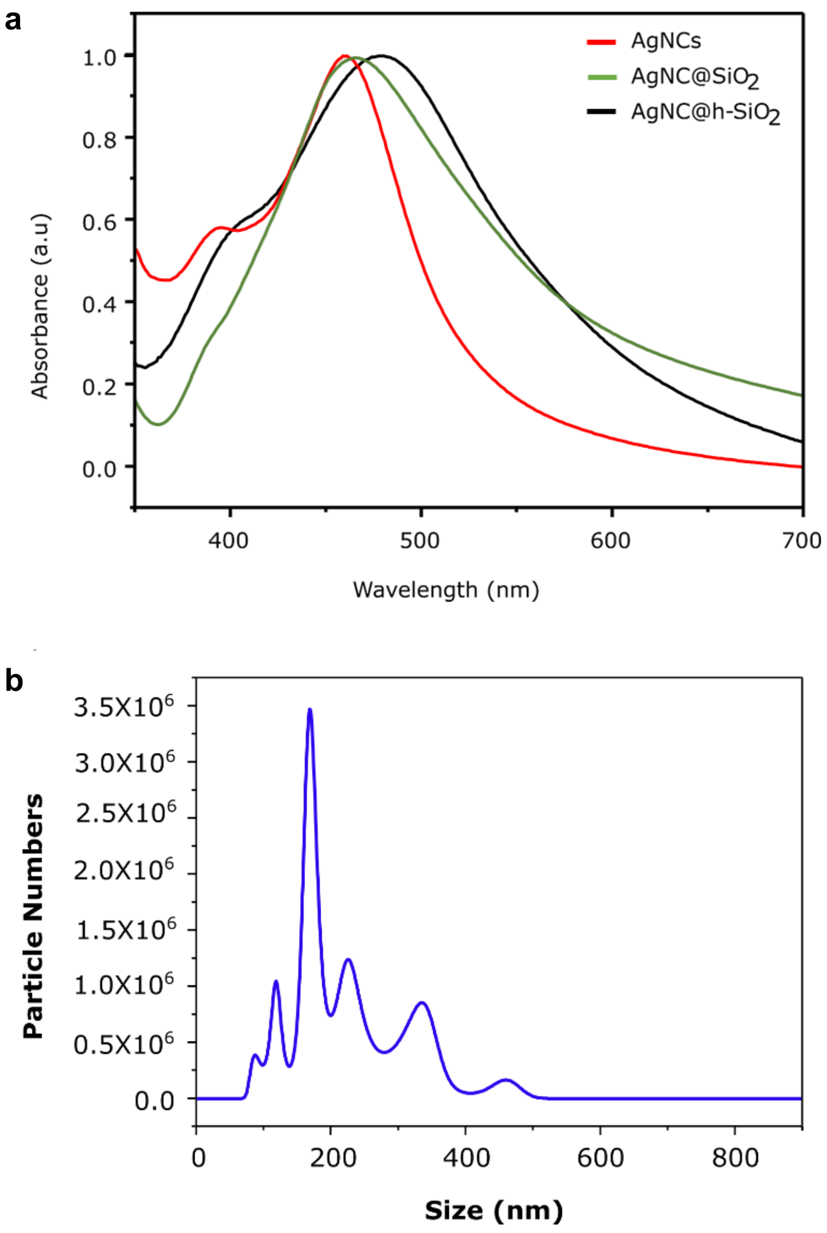

Supplementary Figure 4. Characterizations of AgNC@*h*-SiO_2. (a) UV-vis spectra of AgNCs (black), AgNC@SiO_2 (green), and AgNC@*h*-SiO_2 (Red). (b) nanoparticle size distribution (mean: 224.5 nm) by nanoparticle tracking analyzer (NTA) showing the colloidal stability of AgNC@*h*-SiO_2 nanoparticles.

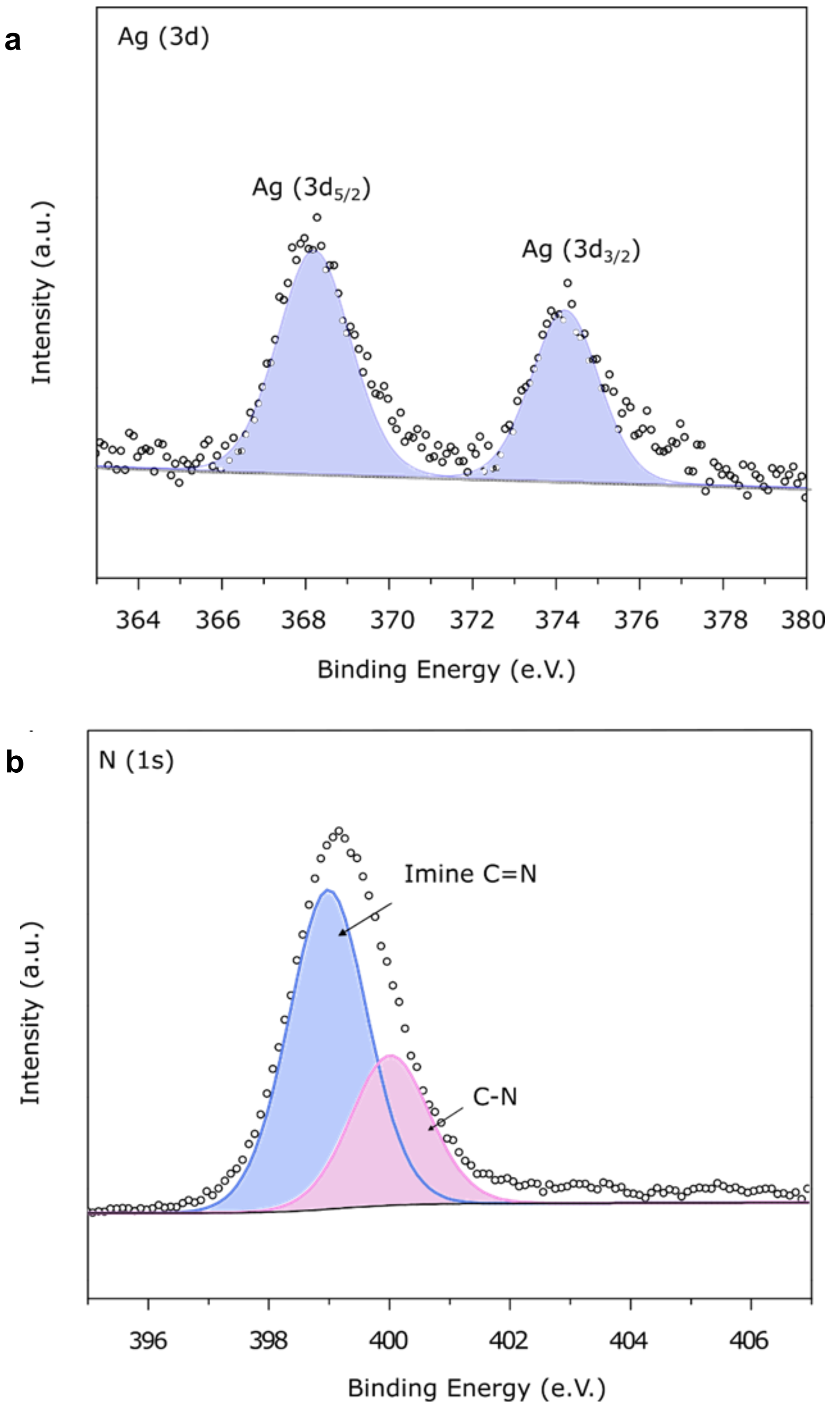

Supplementary Figure 5. X-ray photoelectron spectroscopy (XPS) characterizations of pCOL-AgNC@*h*-SiO$_2$. XPS data of pCOL-AgNC@*h*-SiO$_2$ showing (a) Ag (3d) peaks of the plasmonic core and (b) N (1s) peaks of the p-COL layer.

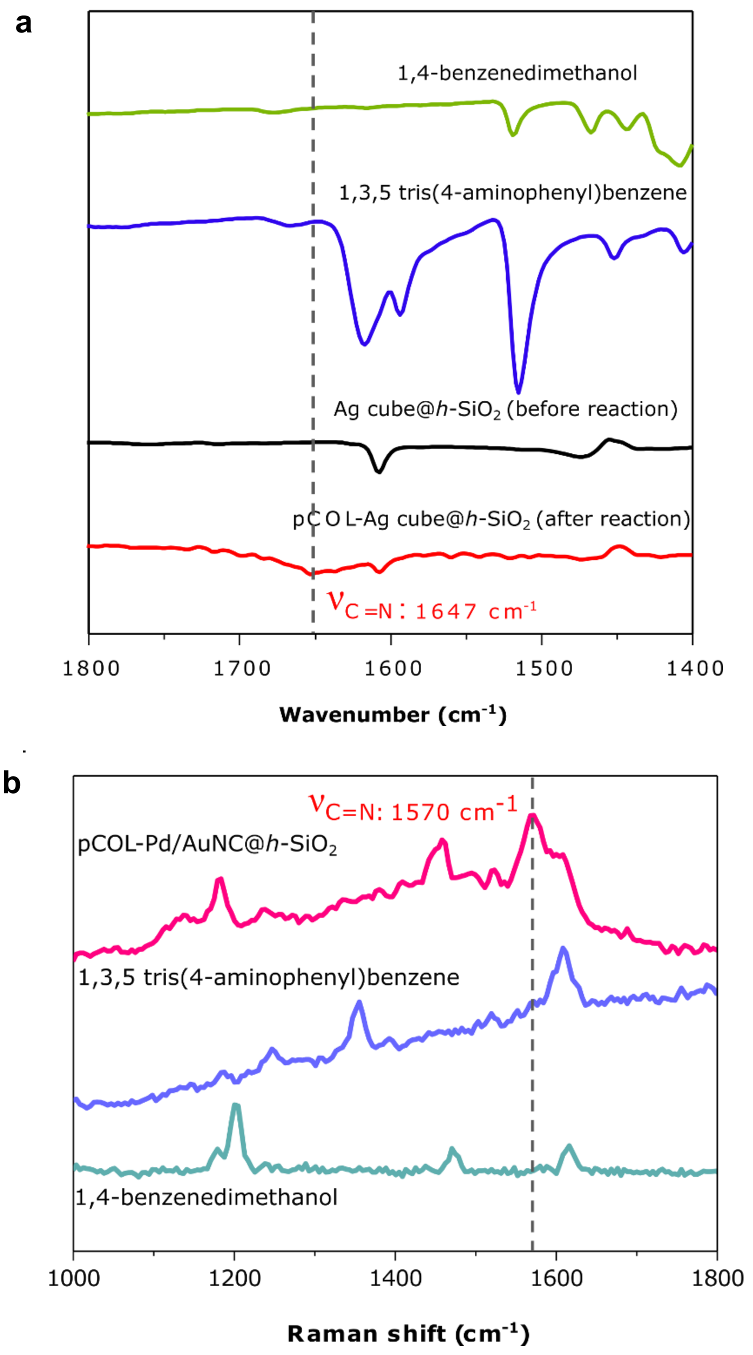

Supplementary Figure 6. Characterizations of pCOL-AgNC@*h*-SiO_2. (a) Fourier transform infrared (FTIR) spectra of pCOL-AgNC@*h*-SiO_2 showing the appearance of imine (C=N peak; 1647 cm^{-1}) functional group after the successful reaction between DAL and TAE. (b) Raman spectra of pCOL-AgNC@*h*-SiO_2 also showing the newly appeared C=N peak at 1570 cm^{-1}. Comparative stacked spectra with the precursors confirmed the successful reaction.

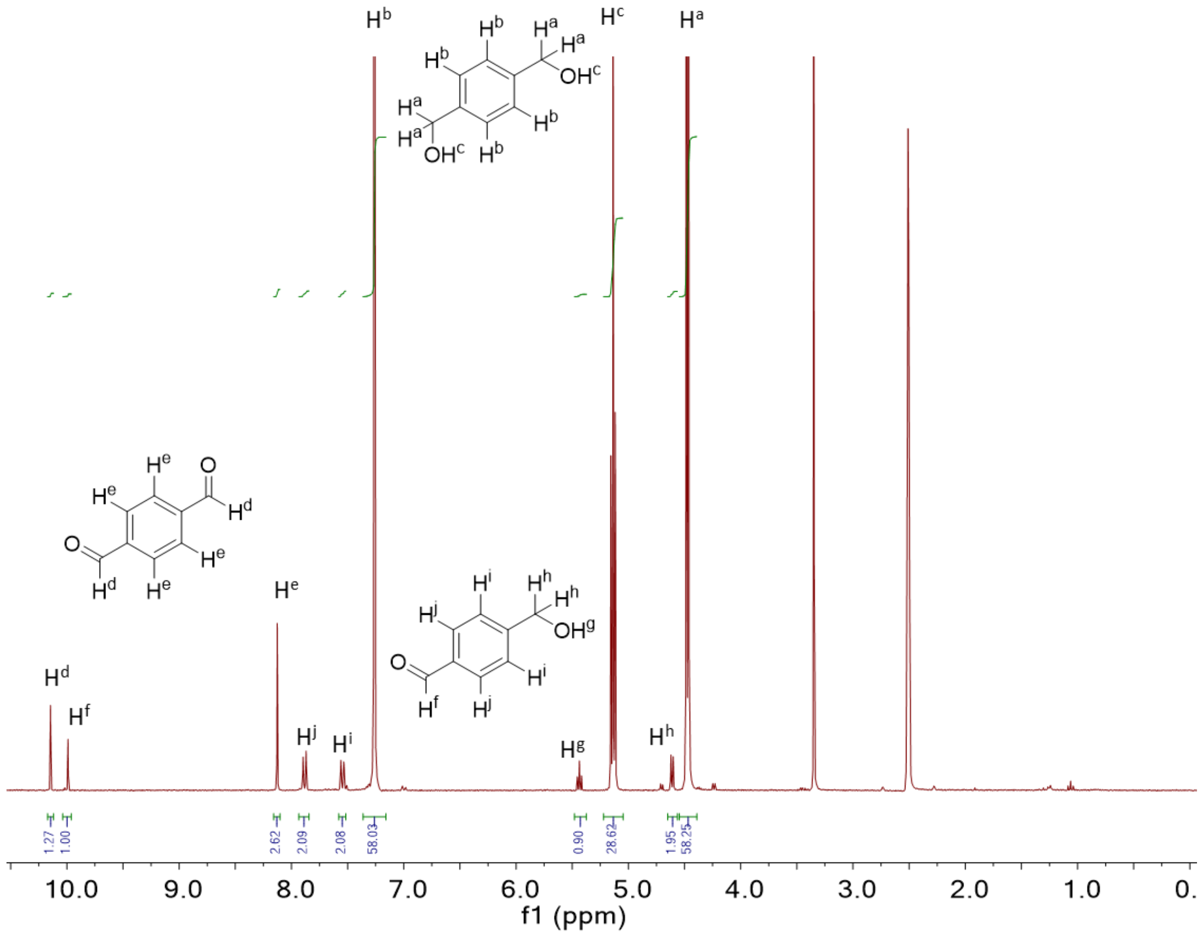

Supplementary Figure 7. AgNC-mediated photochemical oxidation of 1,4-benzenedimethanol (DAL). ^{1}H NMR spectrum of crude reaction mixture showing the oxidized aldehyde products from 1,4-benzenedimethanol under laser irradiation in presence of AgNC@*h*-SiO_2

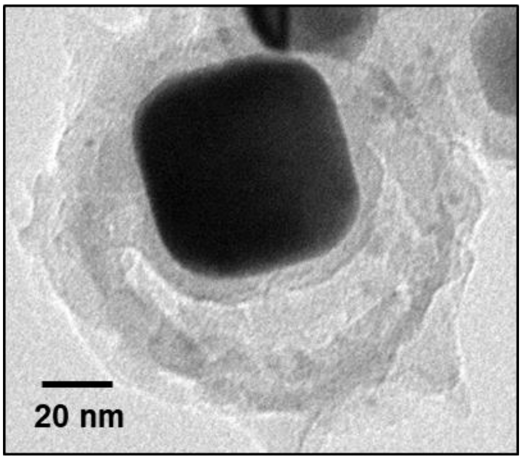

Supplementary Figure 8. Direct condensation of DAE and TAE on AgNC@*h*-SiO_2. TEM image showing non-specific bulk deposition of the cross linked COF product of DAE and TAE on the AgNC-surface as well as on SiO_2 shell.

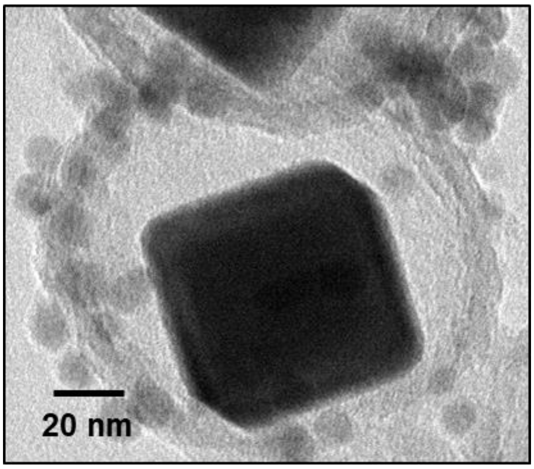

Supplementary Figure 9. Attempt to synthesize pCOL on AgNC@*h*-SiO_2 under external heating. TEM image of the deposited polymeric particulates (*ca*. 10 nm) on AgNC@*h*-SiO_2 under heating condition (100 °C) indicating the uncontrolled oxidation-condensation of DAL and TAE.

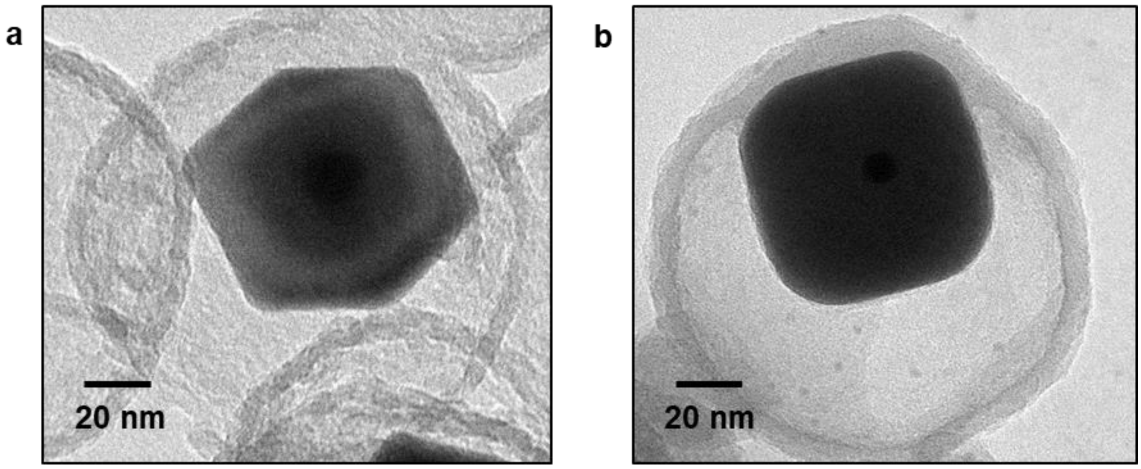

Supplementary Figure 10. Attempt to synthesize pCOL on AgNC@*h*-SiO_2 under different conditions. TEM images of AgNC@*h*-SiO_2 showing no deposition of covalent-organic layers using (a) NIR laser (808 nm, 0.4 W/cm^2) irradiation, and (b) blue laser (405 nm, 0.3 W/cm^2) under inert atmosphere.

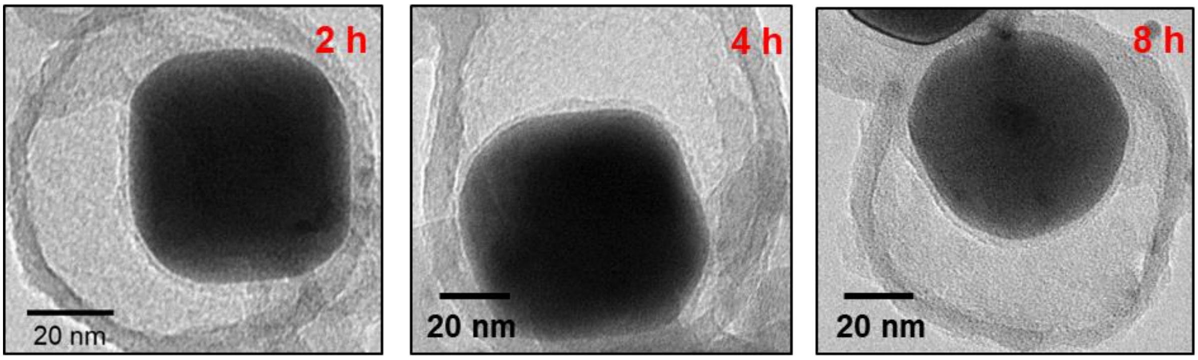

Supplementary Figure 11. Time-dependent pCOL growth on AgNC@*h*-SiO_2. The corresponding time dependent TEM images of pCOL-AgNC@*h*-SiO_2 at the duration of 2h, 4h, and 8h showing consistent thickness (~ 2.5 nm) of p-COL layer on AgNC under the continuous exposure of 405 nm (0.3 W/cm^2) laser.

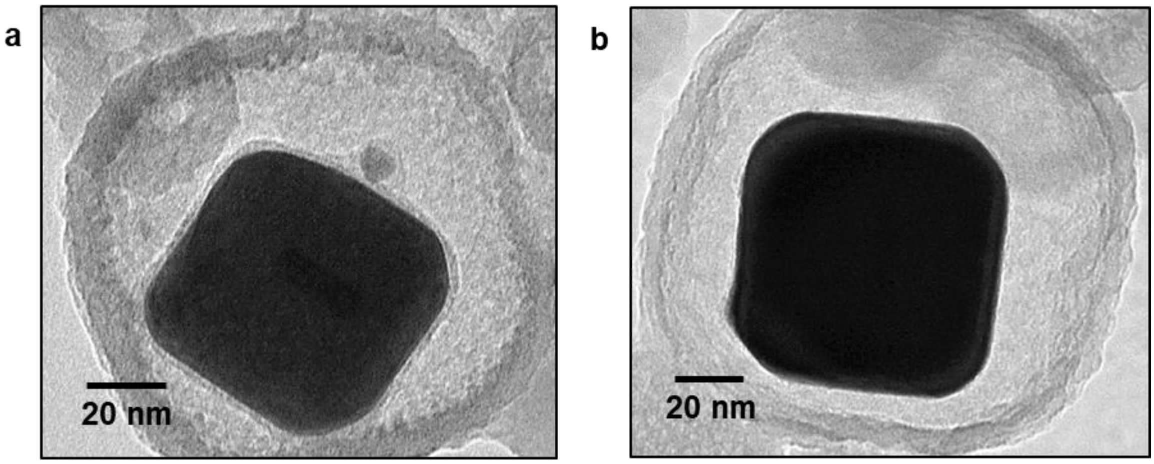

Supplementary Figure 12. Deposition of methyl methacrylate (MMA) polymer on AgNC@*h*-SiO_2. Corresponding TEM images of AgNC@*h*-SiO_2 after MMA deposition under (a) oxygenated environment showing thin polymer layer on AgNC surface, and (b) inert atmosphere showing no polymer deposition on AgNC.

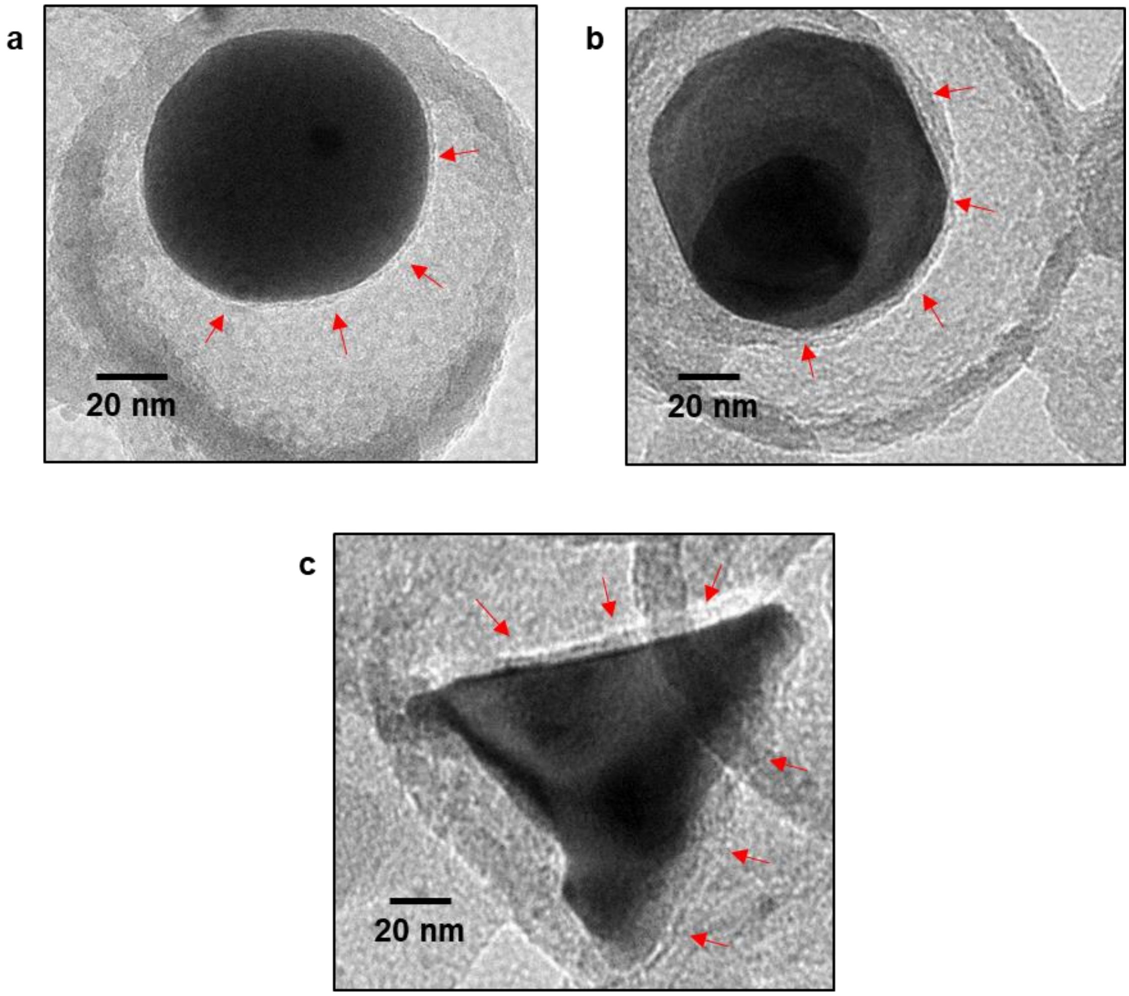

Supplementary Figure 13. pCOL deposition on different plasmonic NC shapes. TEM images of pCOL-AgNC@*h*-SiO_2 having conformal p-COL deposition (red arrows) on (a) spherical (b) dodecahedral and (c) pyramidal AgNC surface.

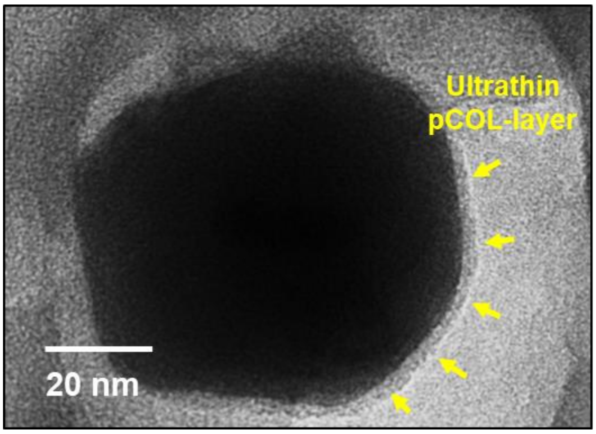

Supplementary Figure 14. pCOL deposition on AuNC@*h*-SiO_2 under irradiation of 532 nm laser. TEM image of pCOL-AuNC@*h*-SiO_2 having conformal p-COL deposition on AuNCs under the irradiation of 532 nm laser.

a

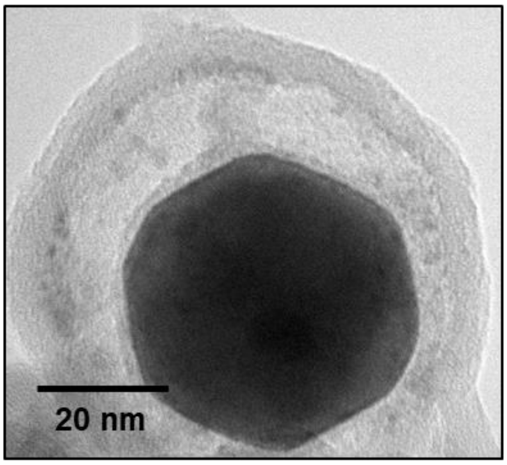

Element	Weight %	Atomic %
Pd	4.09	7.32
Au	95.91	92.68

b

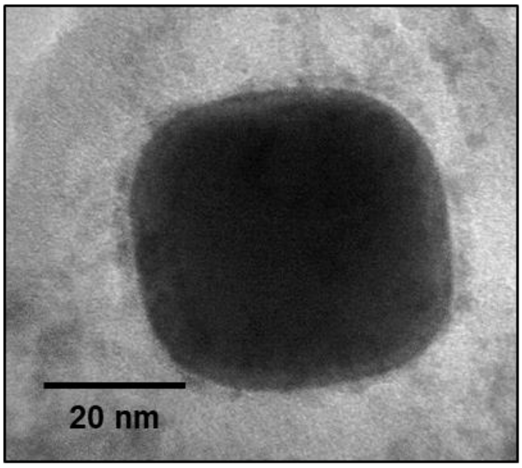

Element	Weight %	Atomic %
Pd	4.44	7.92
Au	95.56	92.08

c

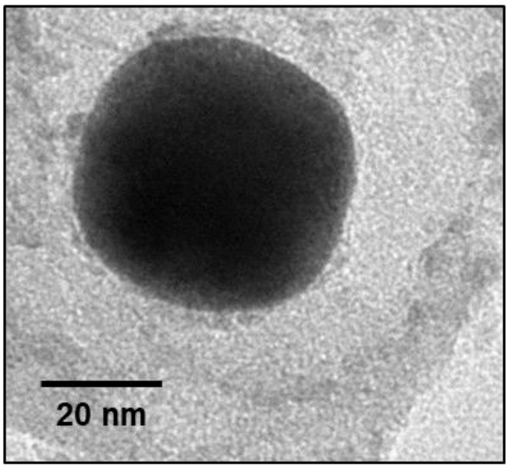

Element	Weight %	Atomic %
Pd	3.93	7.03
Au	96.07	92.97

d

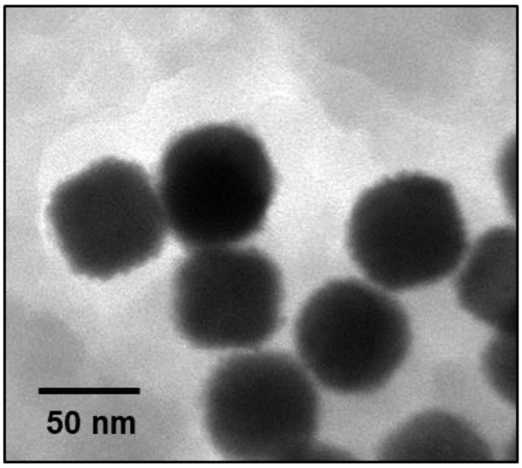

Element	Weight %	Atomic %
Pd	5.76	10.16
Au	94.24	89.84

Supplementary Figure 15. Characterization of control catalysts. TEM images of (a) pCOL-Pd/AuNC@*h*-SiO_2, (b) TAE-Pd/AuNC@*h*-SiO_2, (c) Pd/AuNC@*h*-SiO_2, and (d) Bulk COF-Pd/AuNC with their corresponding EDS-based weight and atomic% of Pd and Au respectively.

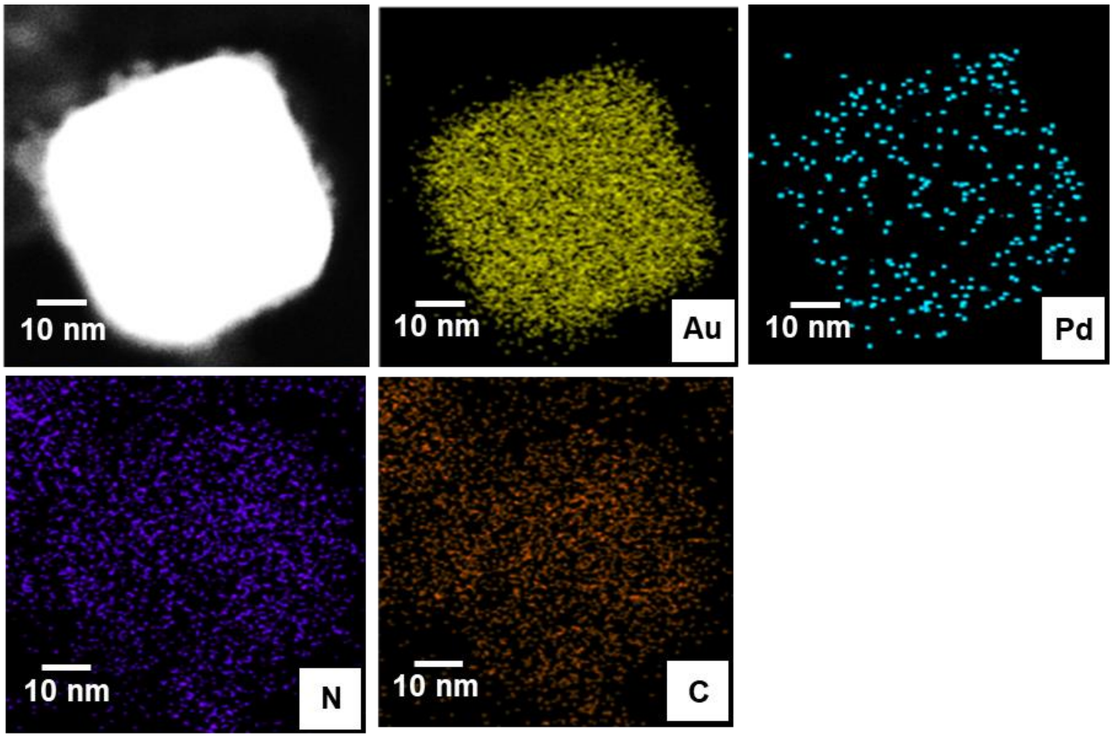

Supplementary Figure 16. Characterization of pCOL-Pd/AuNC (SiO_2 removed)**.** HAADF-STEM image and EDS-based elemental mapping of pCOL-Pd/AuNC (SiO_2 removed)

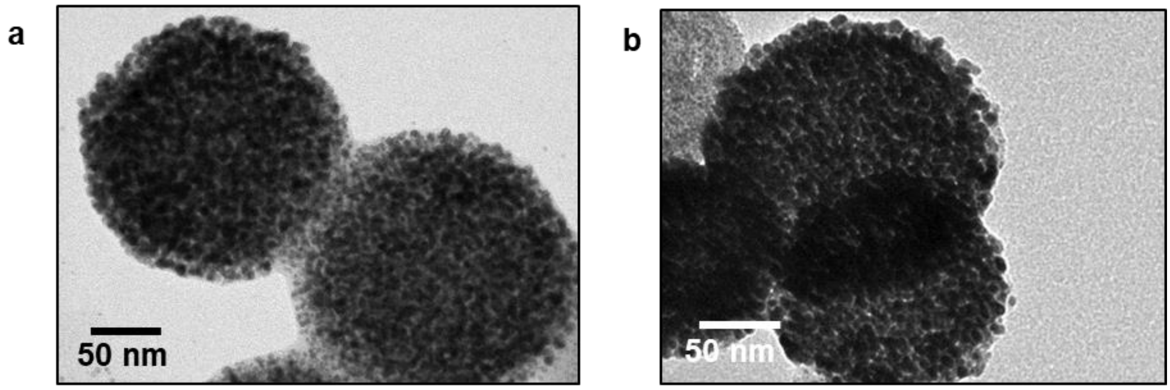

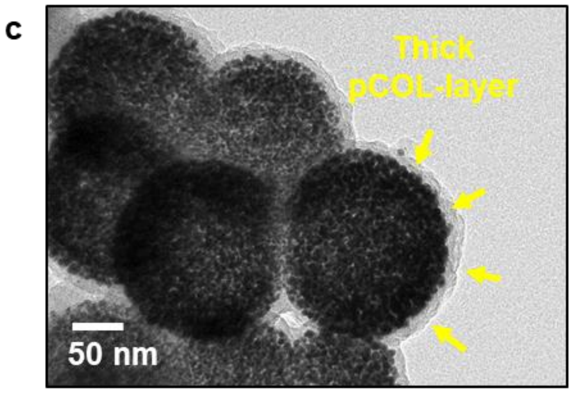

Element	Weight%	Atomic%
N K	6.18	23.64
C K	25.16	47.98
Au L	41.94	21.11
Pd L	26.71	7.26

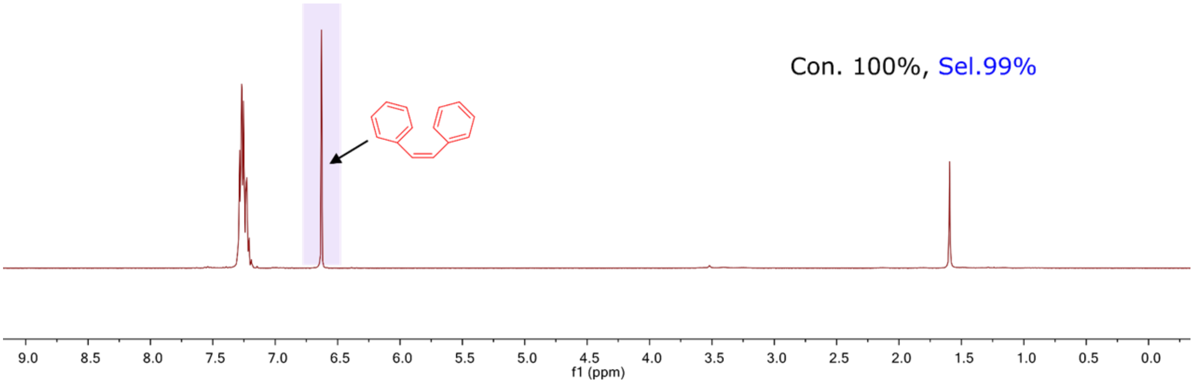

Supplementary Figure 17. Step-wise synthesis and characterization of pCOL-Pd/AuNP@SiO$_2$. TEM image of (a) AuNP@SiO$_2$ (b) Pd/AuNP@SiO$_2$ and (c) pCOL-Pd/AuNP@SiO$_2$ and their corresponding EDS-based weight and atomic% of N, C, Au and Pd in the table. ^{1}H NMR data of the catalytic semihydrogenation of diphenylacetylene using pCOL-Pd/AuNP@SiO$_2$ under laser irradiation resulting to the alkene as major product with 99% selectivity.

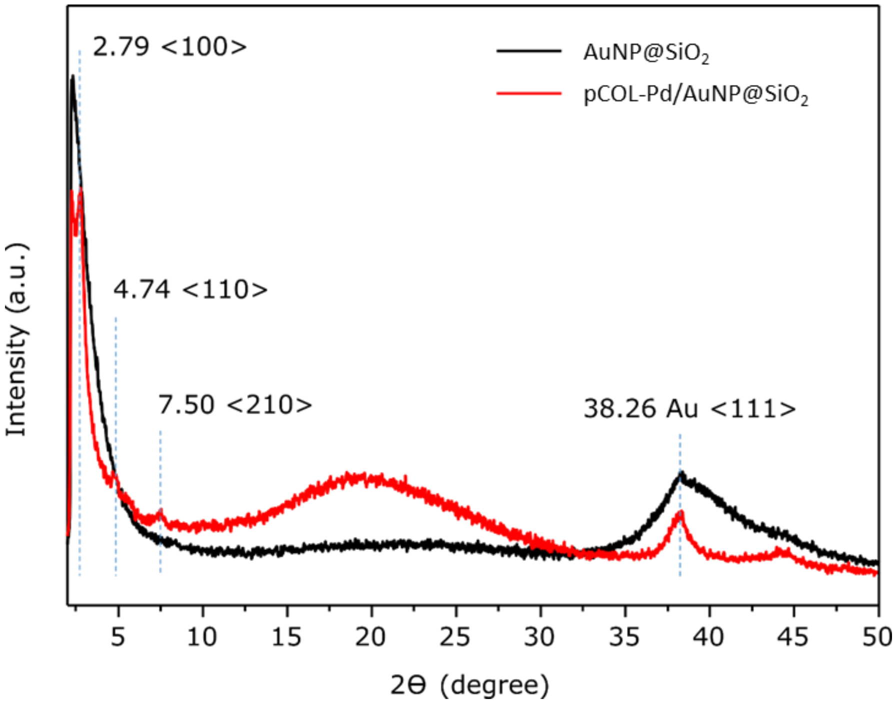

Supplementary Figure 18. XRD data of pCOL-Pd/AuNP@SiO_2. The XRD data of pCOL-Pd/AuNP@SiO_2 before and after the pCOL modification.

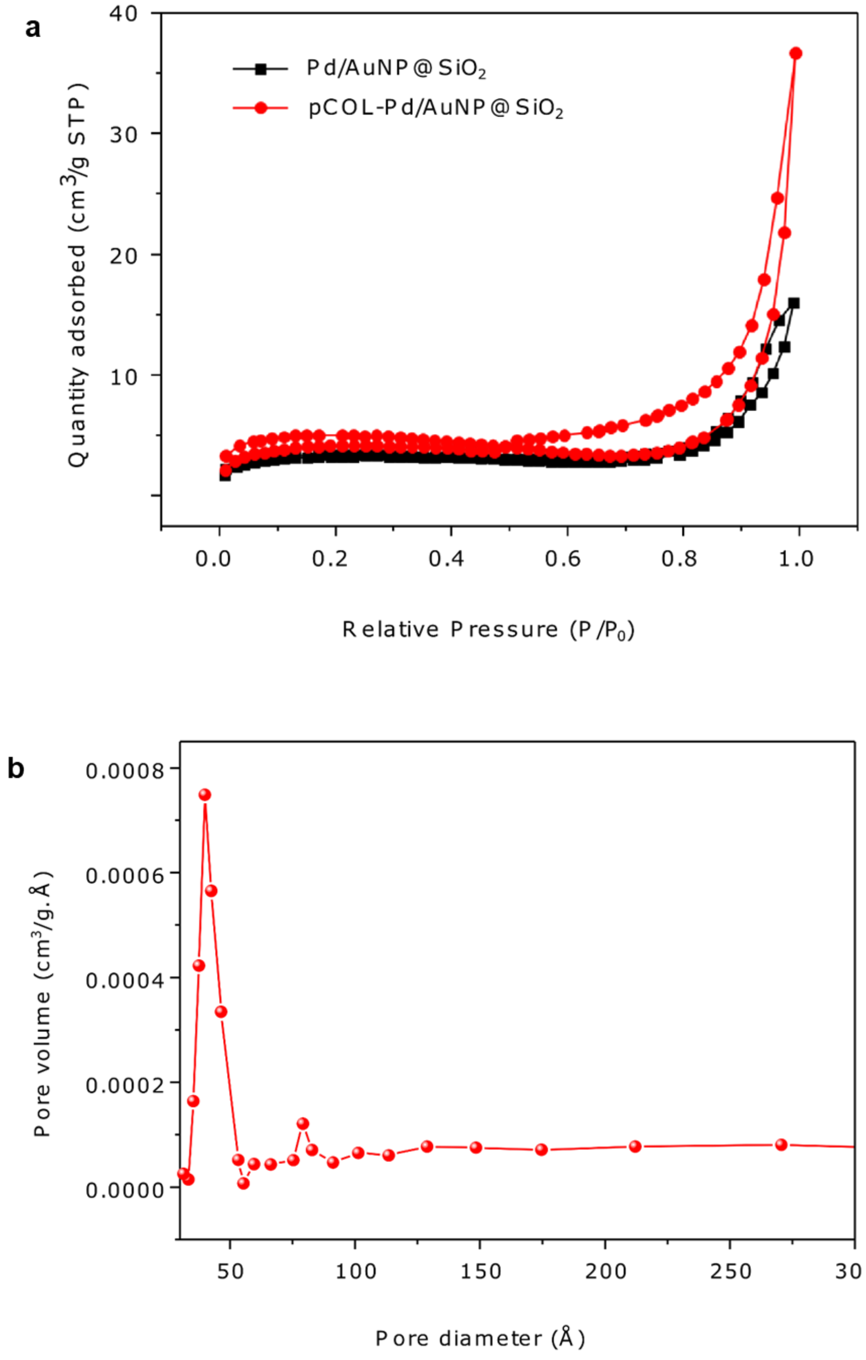

Supplementary Figure 19. Porous structure analysis of pCOL-Pd/AuNP@SiO_2 (a) N_2 adsorption-desorption isotherm profile of Pd/AuNP@SiO_2 (BET surface area: 10 m^2/g) and pCOL-Pd/AuNP@SiO_2 (BET surface area: 19 m^2/g) before and after pCOL deposition respectively. (b) Pore size distribution (average pore size 4 nm) of pCOL layer in pCOL-Pd/AuNP@SiO_2.

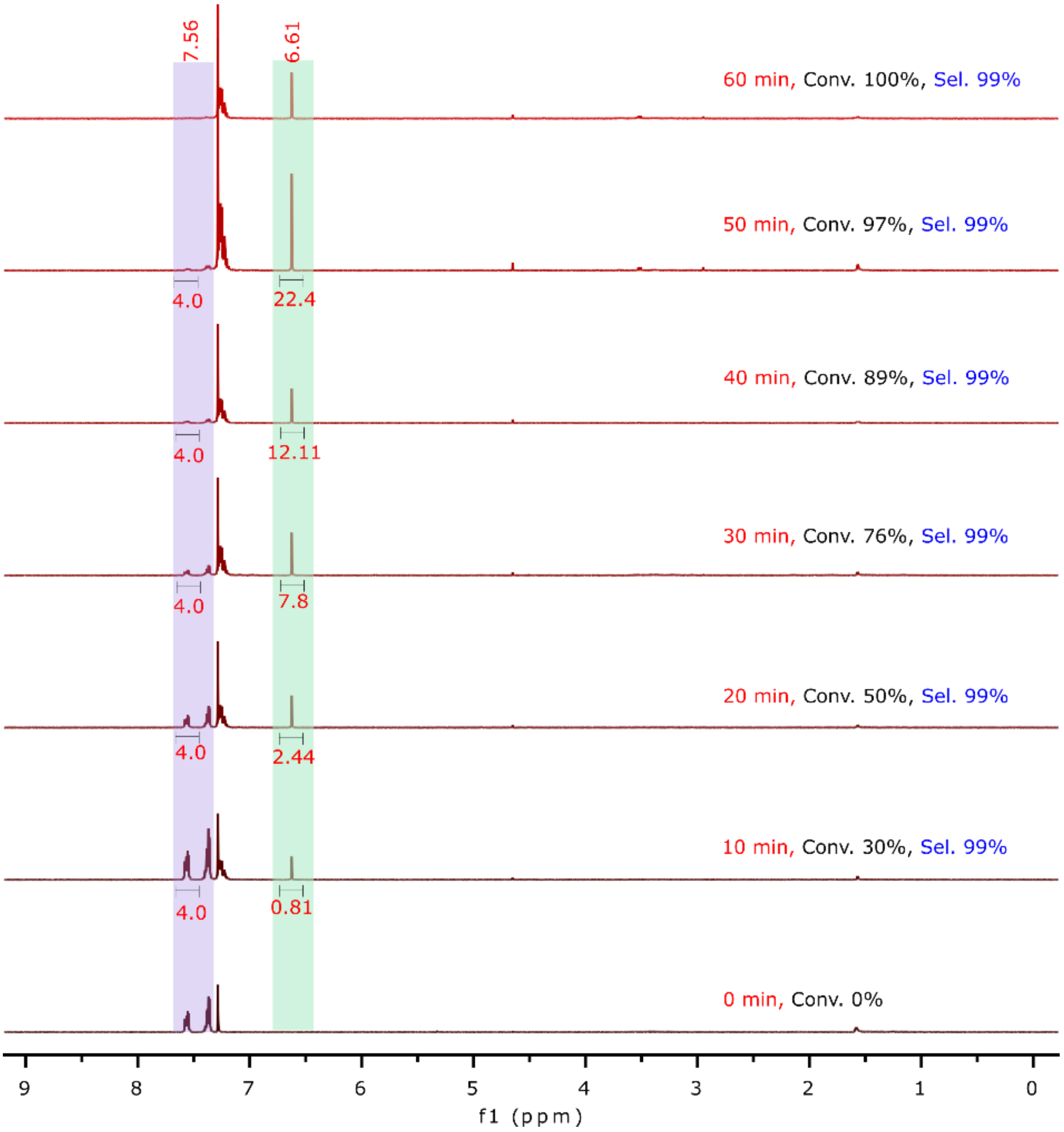

Supplementary Figure 20. Catalytic performance of pCOL-Pd/AuNC@*h*-SiO_2 during the semi-hydrogenation of diphenylacetylene. Time dependent stacked ^{1}H NMR data for the stepwise evolution of semi-hydrogenated products from alkyne (diphenylacetylene) with consistent high selectivity towards forming alkene (stilbene) as a main product using pCOL-Pd/AuNC@*h*-SiO_2 under the exposure of 405 nm laser (0.3 W/cm^2).

No	Conc. of Mesitylene (c_i) (M)	Area of Mesitylene (a_i)	Conc. of Diphenylacetylene (c_x) (M)	Area of Diphenylacetylene (a_x)	$R_f = (a_x/a_i)/(c_x/c_i)$
1	0.03 M	148580387	0.1 M	358165608	0.58664
2	0.03 M	169658485	0.07 M	296190770	
3	0.03 M	169447354	0.05 M	296405612	
4	0.03 M	169530429	0.03 M	179819747	
5	0.03 M	158194984	0.01 M	96209733	

No	Conc. of Mesitylene (c_i) (M)	Area of Mesitylene (a_i)	Conc. of Cis-stilbene (c_x) (M)	Area of Cis-stilbene (a_x)	$R_f = (a_x/a_i)/(c_x/c_i)$
1	0.03 M	159236050	0.1 M	382695734	0.58846
2	0.03 M	152214857	0.07 M	327690963	
3	0.03 M	161956883	0.05 M	268247803	
4	0.03 M	156543616	0.03 M	211318228	
5	0.03 M	167249591	0.01 M	106068433	

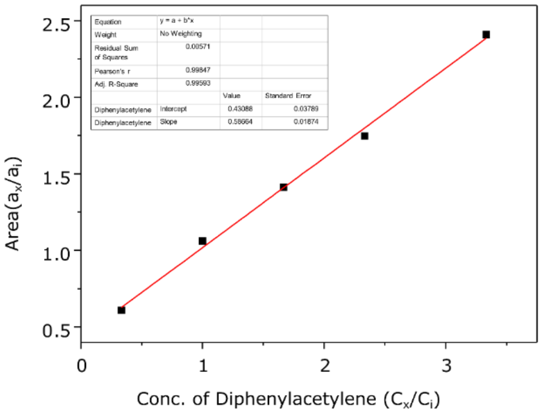

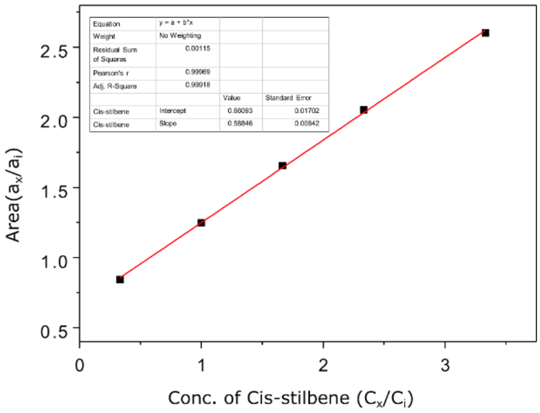

Supplementary Figure 21. Response factor calculation for GC-MS analysis. Determination of response factor (R_f) for diphenylacetylene and cis-stilbene.

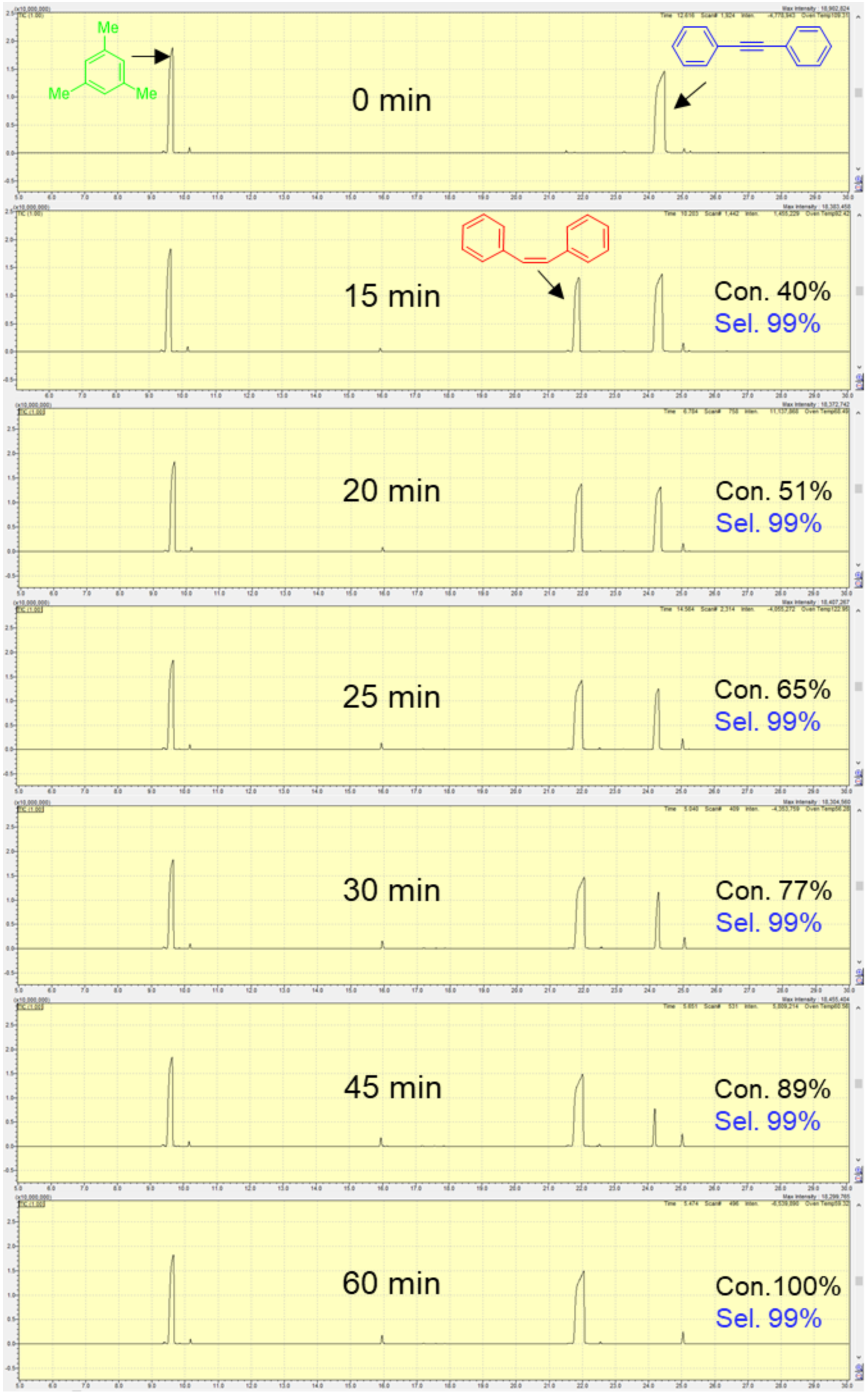

Supplementary Figure 22. GC analysis for the catalytic semihydrogenation of diphenylacetylene using pCOL-Pd/AuNC@*h*-SiO$_2$. Time dependent stacked GC chromatographs showing the stepwise evolution of semi-hydrogenated products from alkyne (diphenylacetylene) in presence of mesitylene as internal standard, with consistent high selectivity towards forming alkene (stilbene) as a main product using pCOL-Pd/AuNC@*h*-SiO$_2$ under the exposure of 405 nm laser (0.3 W/cm^2).

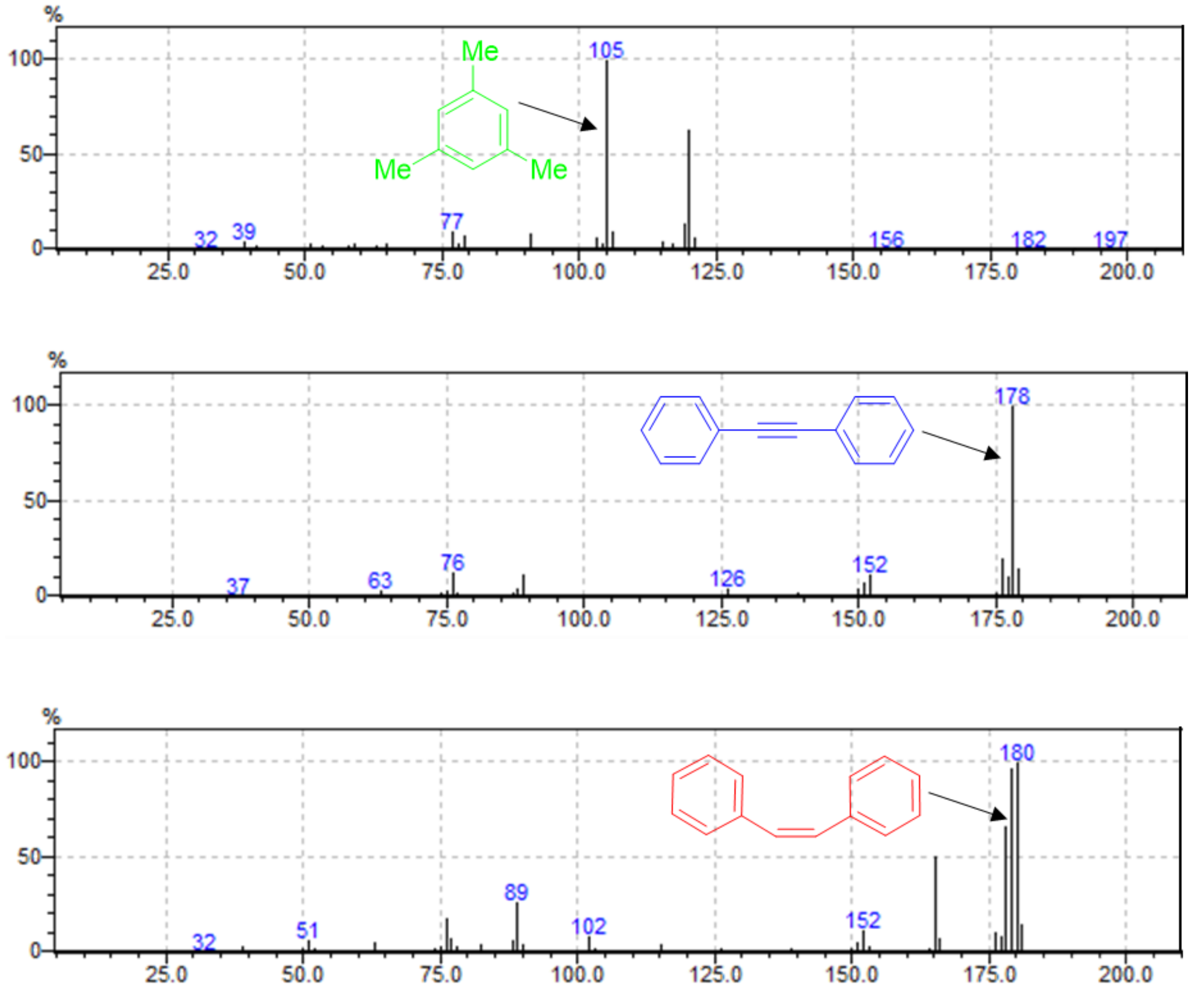

Supplementary Figure 23. Mass-spectrometry results. Mass spectrometry data of the chromatographic peaks acquired during the semihydrogenation reaction of alkyne (diphenylacetylene) in presence of mesitylene as internal standard, with consistent high selectivity towards forming alkene (stilbene) as a main product using pCOL-Pd/AuNC@*h*-SiO_2 under the exposure of 405 nm laser (0.3 W/cm^2).

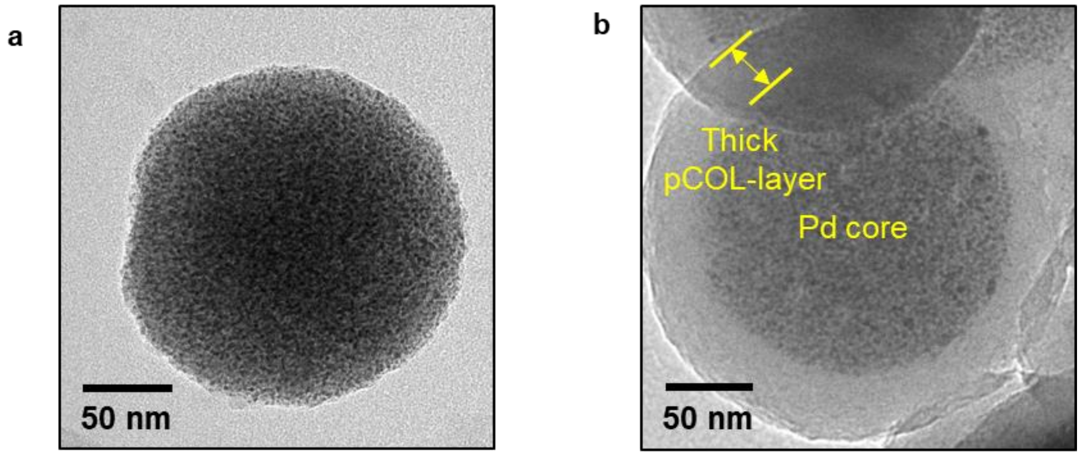

Supplementary Figure 24. Characterization of thick pCOL-PdNP@SiO_2. TEM image showing (a) PdNP@SiO_2 and (b) after the deposition of thick pCOL layer as PdNP@SiO_2

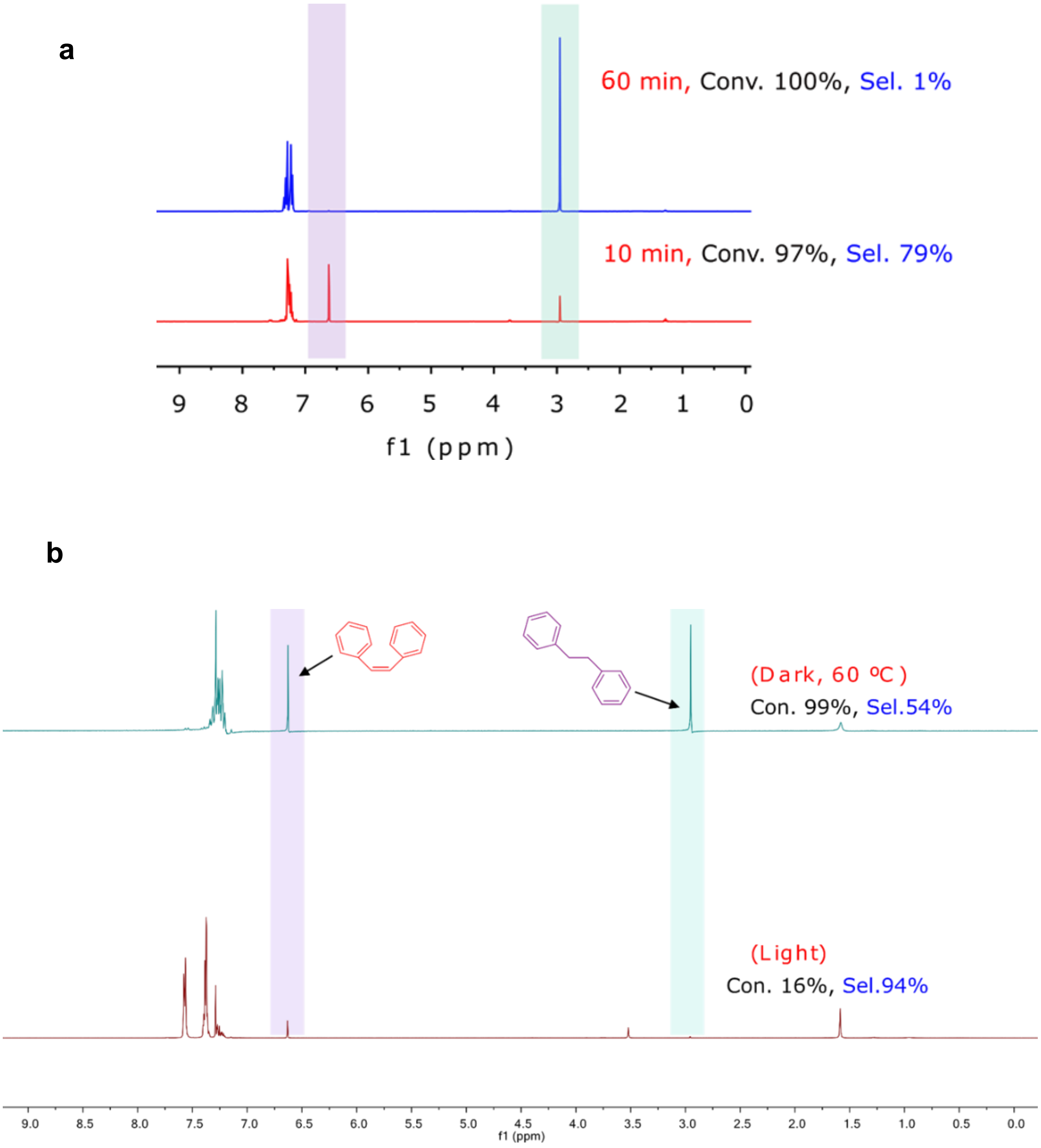

Supplementary Figure 25. Controlled catalytic reaction with commercial Pd/C and thick pCOL--PdNP@SiO$_2$. Time-dependent stacked ^{1}H NMR data showing the over-hydrogenation of diphenylacetylene using the (a) commercial Pd/C under 60 ˚C temperature and (b) thick pCOL-PdNP@SiO$_2$ under light and dark (60 ˚C, 90 min) condition.

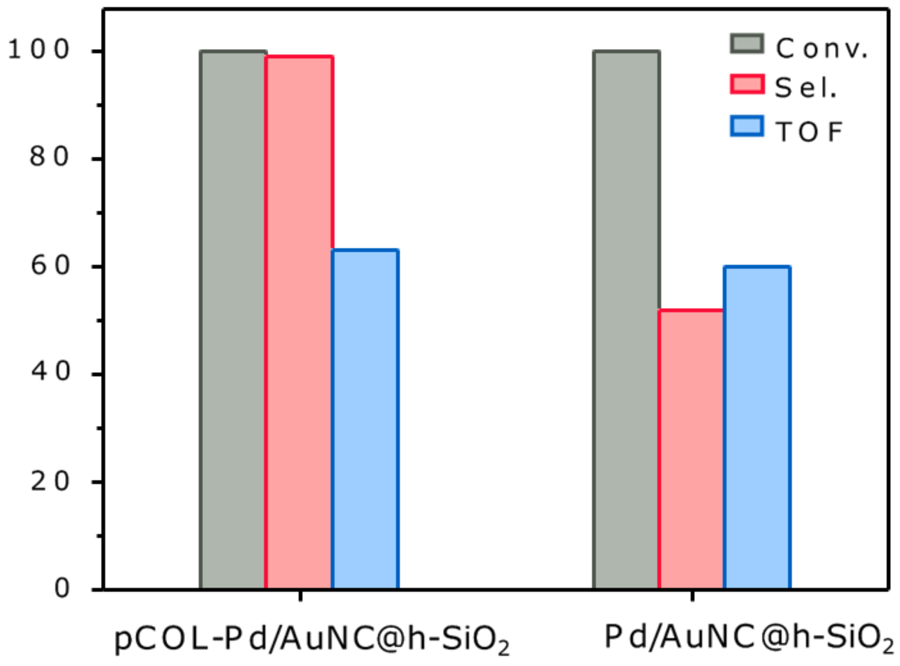

Supplementary Figure 26. Turn over frequency (TOF) calculation. TOF (min^{-1}) data and corresponding conversion and selectivity (%) calculated for the semihydrogenation reaction from alkyne (diphenylacetylene) to alkene (stilbene) using pCOL-Pd/AuNC@*h*-SiO_2 and Pd/AuNC@*h*-SiO_2 under the exposure of 405 nm laser (0.3 W/cm^2).

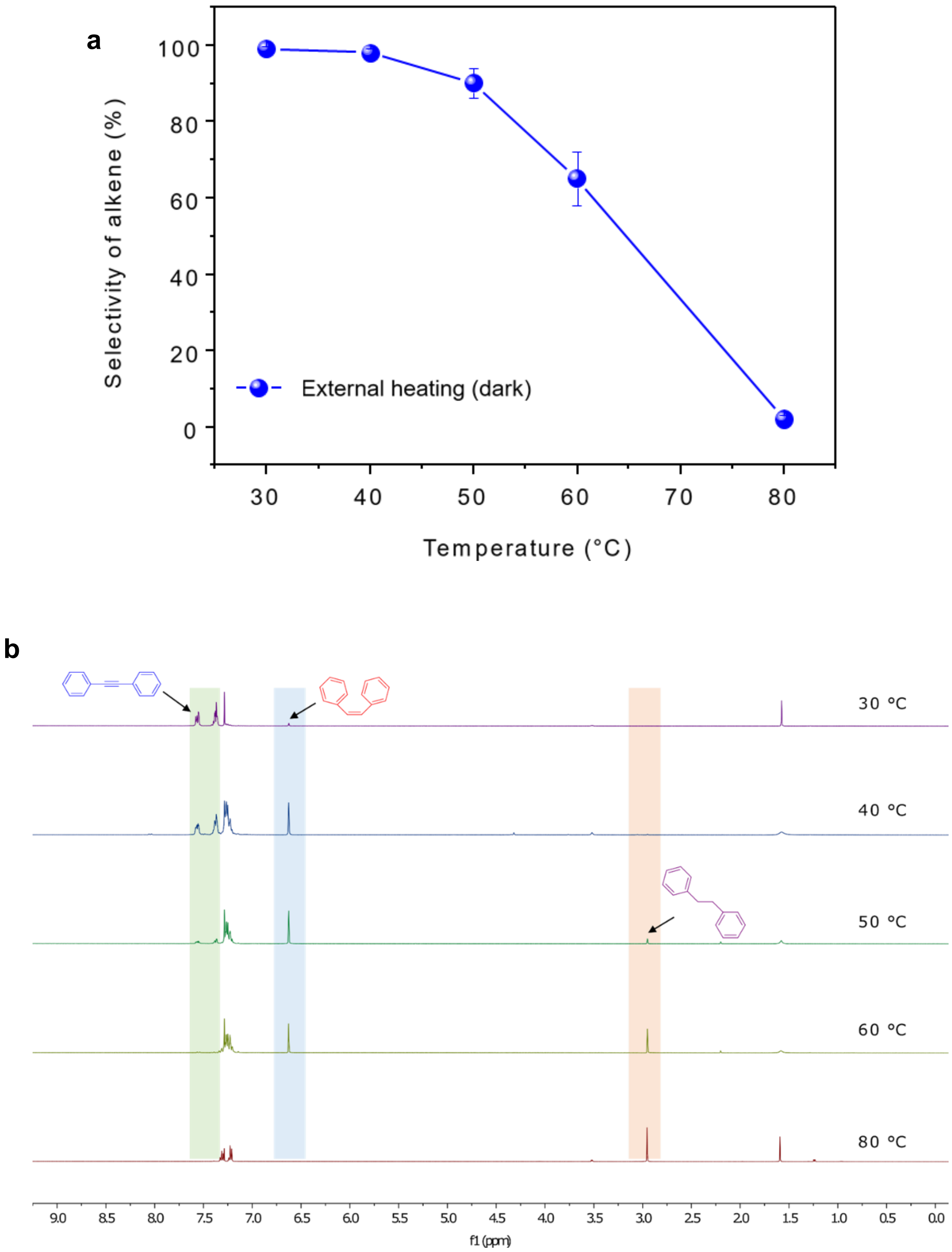

Supplementary Figure 27. Catalysis under external heating condition. (a) Alkene selectivity plot and (b) the corresponding ^{1}H NMR data of variable selectivities of the alkene product from alkyne (diphenylacetylene) using pCOL-Pd/AuNC@*h*-SiO_2 by raising the reaction temperatures from 30 to 80 °C.

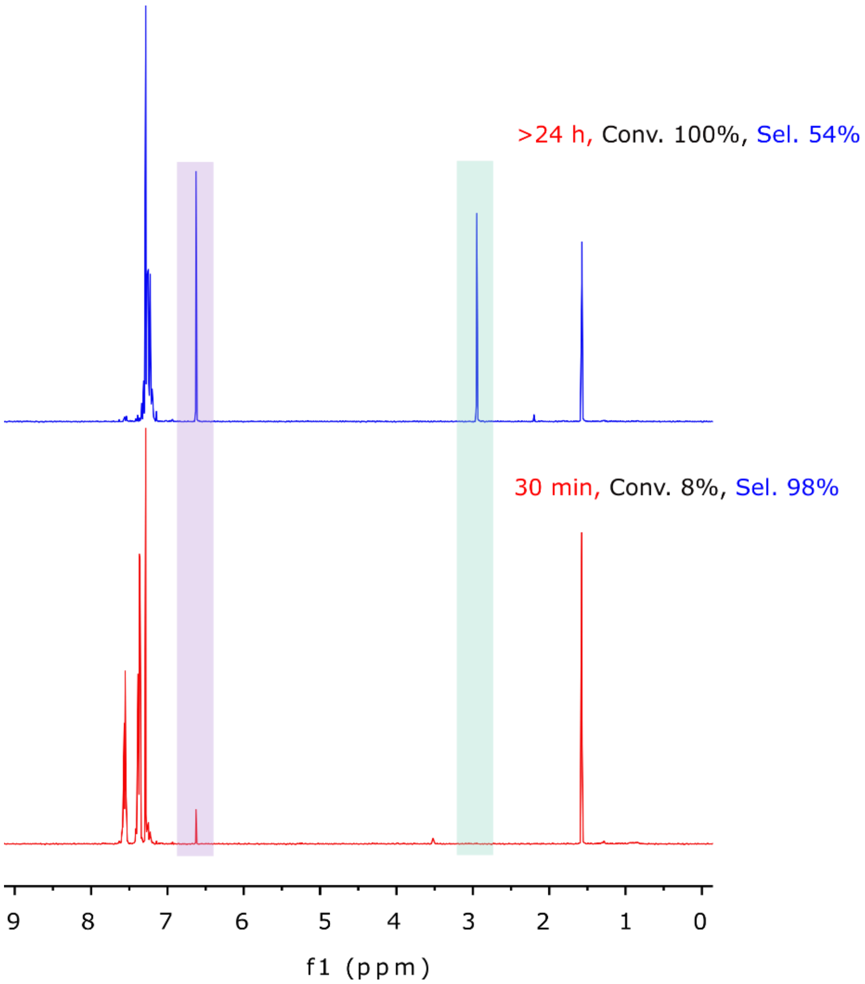

Supplementary Figure 28. Controlled catalytic reactions at dark condition. Time dependent ^{1}H NMR spectra after continuing the hydrogenation of diphenylacetylene under dark condition at 30 min and 24 h using catalyst pCOL-Pd/AuNC@*h*-SiO_2.

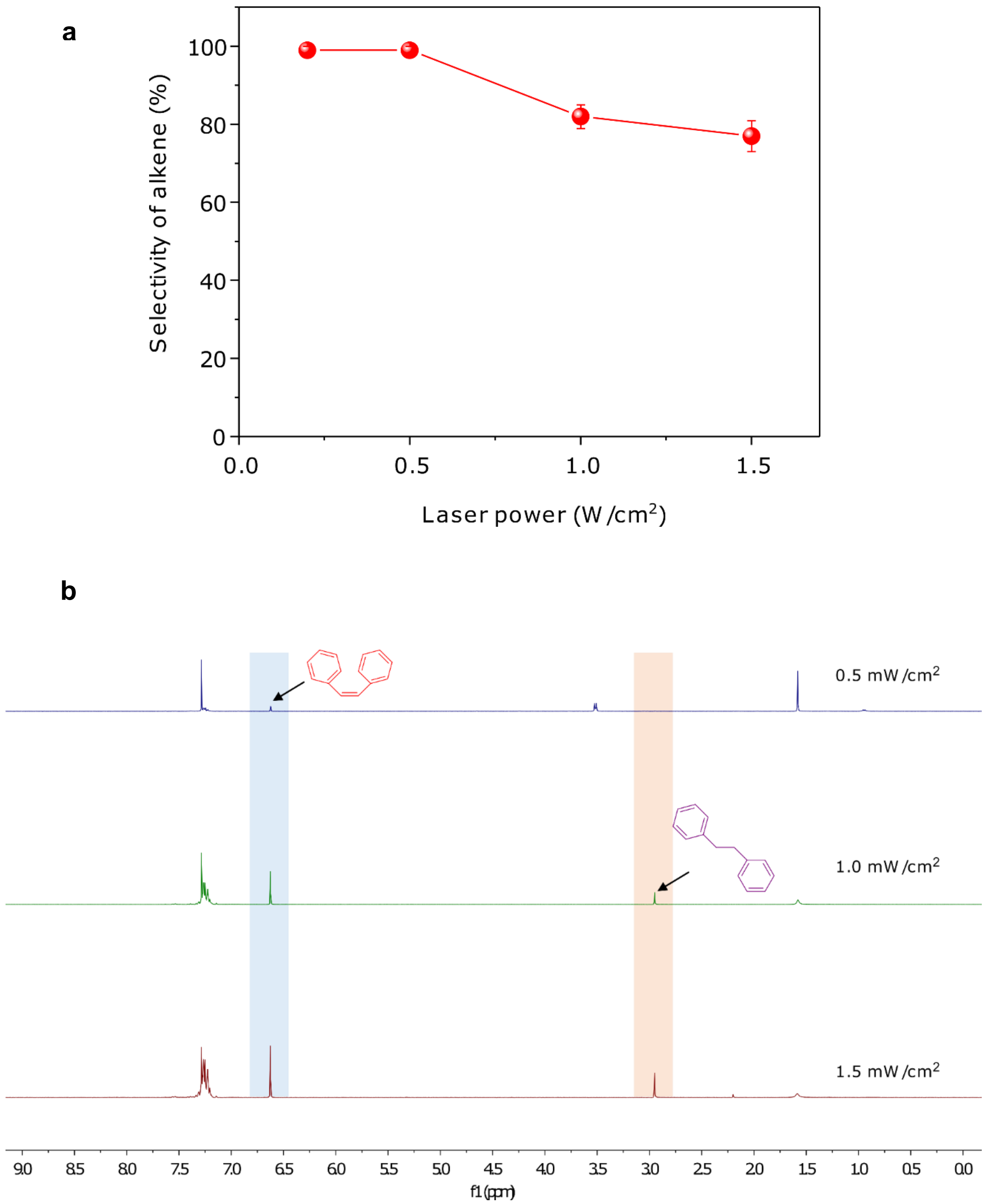

Supplementary Figure 29. Catalysis under variable laser flux. (a) Selectivity plot of the alkene product from alkyne (diphenylacetylene) using pCOL-Pd/AuNC@*h*-SiO_2 sequentially increasing the 405 nm laser flux. (b) Corresponding ^{1}H NMR data with sequential increase in the laser flux.

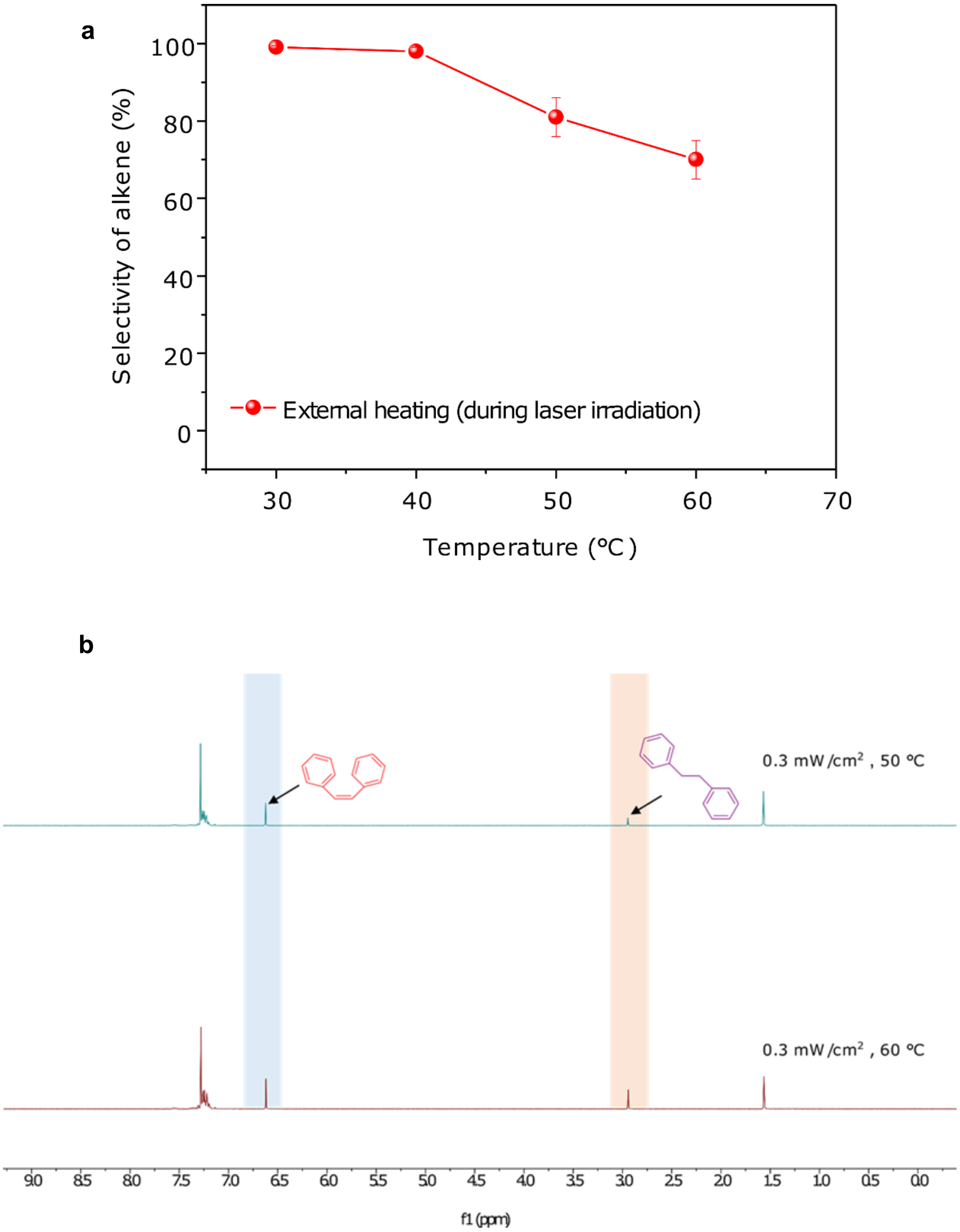

Supplementary Figure 30. Catalysis external heating during the laser irradiation. (a) Alkene selectivity plot and (b) the corresponding ^{1}H NMR data of variable selectivities of the alkene product from alkyne (diphenylacetylene) using pCOL-Pd/AuNC@*h*-SiO_2 with sequential increase in external heating external heat up to 60 °C during the laser irradiation (405 nm; 0.3 W/cm^2).

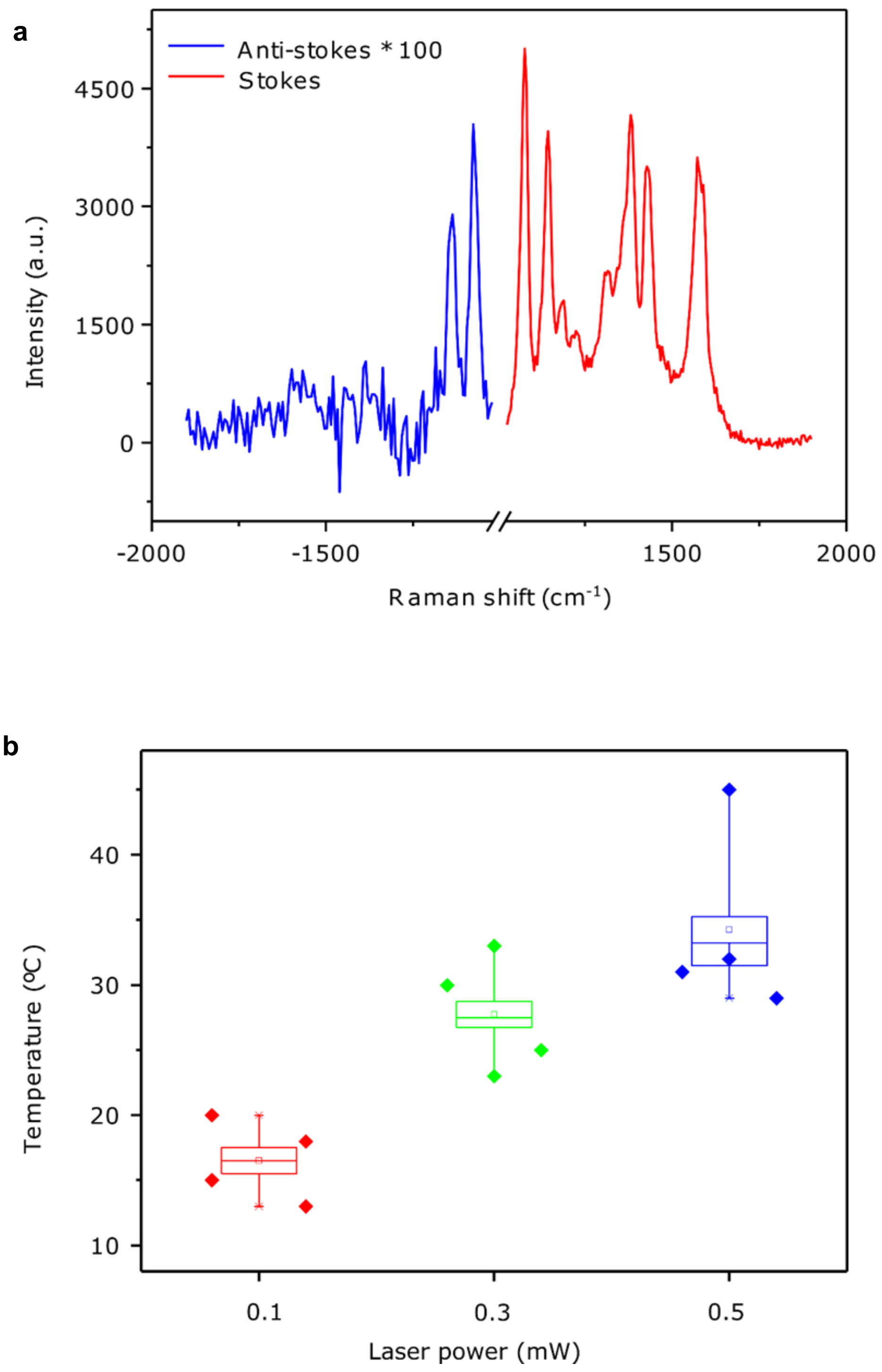

Supplementary Figure 31. Raman thermometry analysis. (a) Representative SERS signals (stokes and antistokes) of 4-aminothiphenol tethered on pCOL-Pd/AuNCs surface. (b) Localized temperatures on plasmonic catalyst's (pCOL-Pd/AuNC) surface under the irradiation of continuous wave laser.

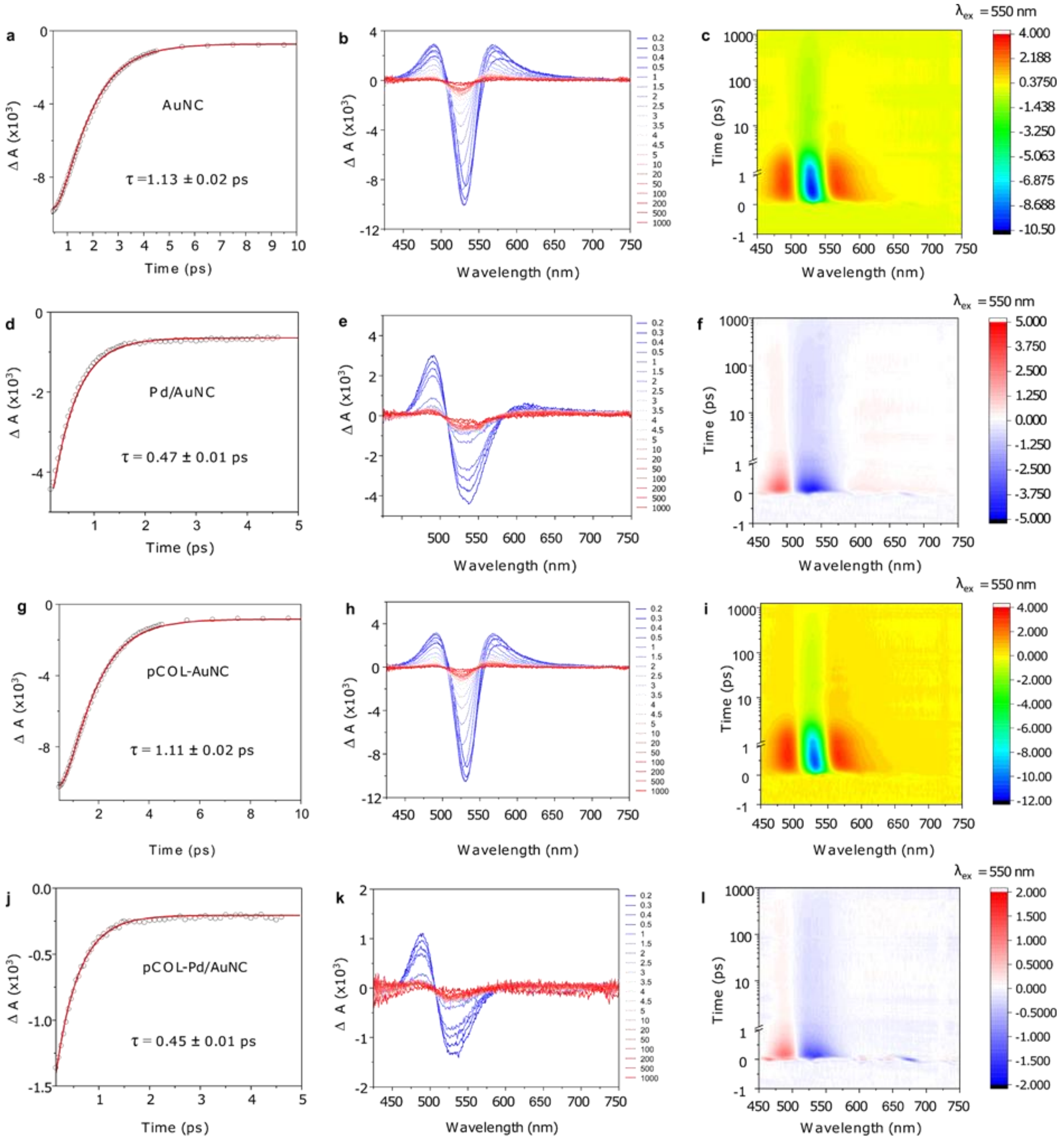

Supplementary Figure 32. Transient absorption (TA) analysis. Excited charge carrier dynamics data showing the life time, TA-data plot corresponds to charge carrier generation efficiencies and TA-contour plot for (a,b and c) AuNC, (d,e and f) Pd/AuNC, (g,h and i) pCOL-AuNC, (j,k and l) pCOL-Pd/AuNC respectively.

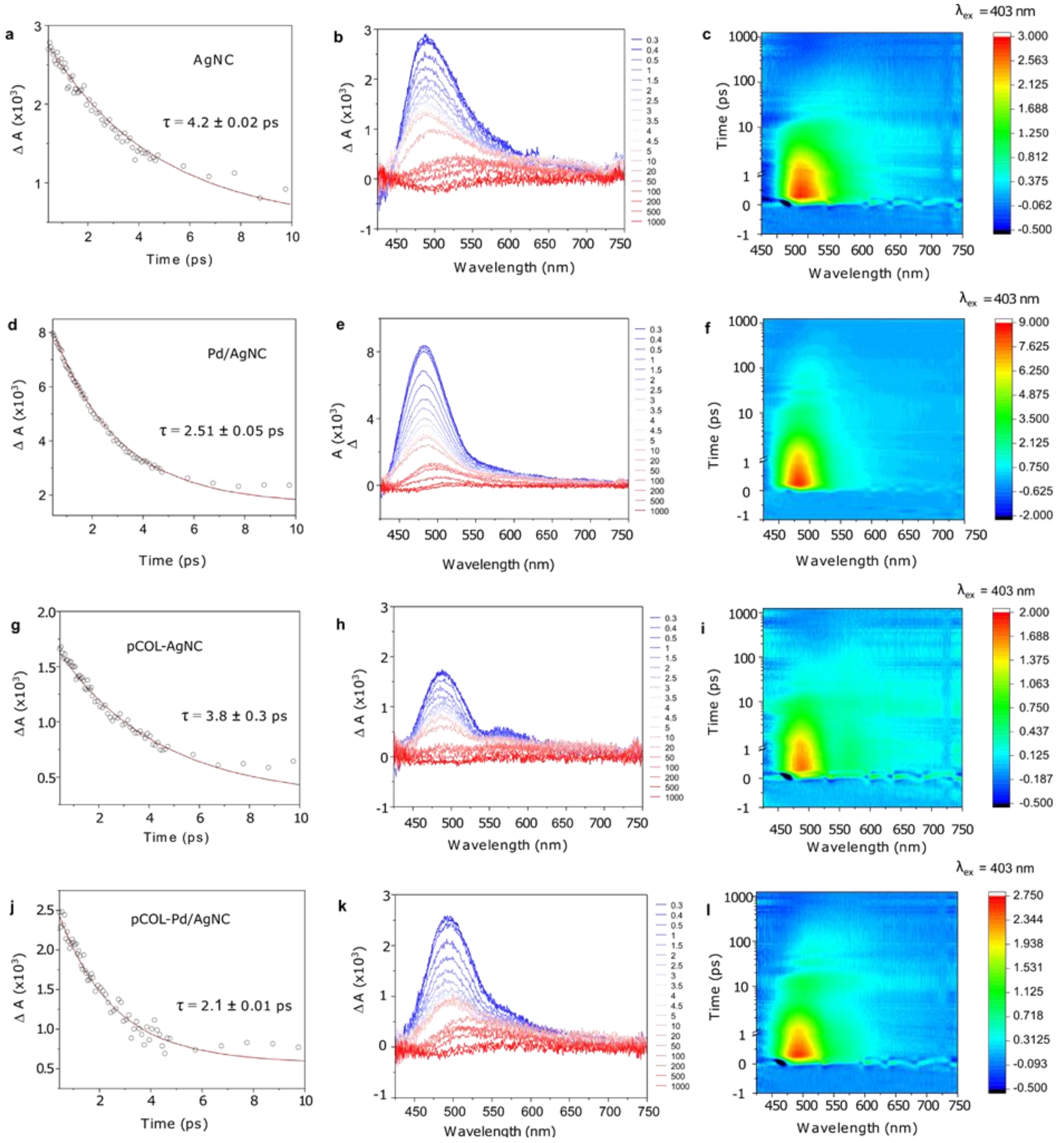

Supplementary Figure 33. Transient absorption (TA) analysis. Excited charge carrier dynamics data showing the life time, TA-data plot corresponds to charge carrier generation efficiencies and TA-contour plot for (a,b and c) AgNC, (d,e and f) Pd/AgNC, (g,h and i) pCOL-AgNC, (j,k and l) pCOL-Pd/AgNC respectively.

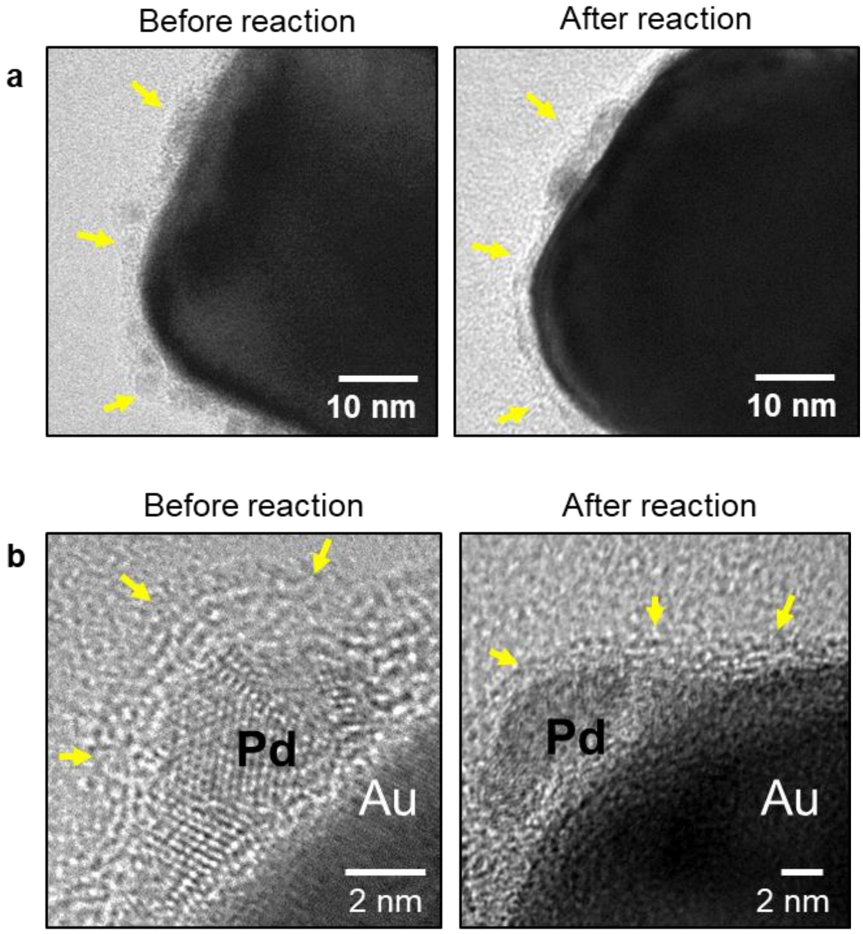

c

No	Name	Au content (Wt %)	Pd content (Wt %)
1	*p*COL-Pd/AuNC (Before reaction)	97.01	2.99
2	*p*COL-Pd/AuNC (After reaction)	97.43	2.56

Supplementary Figure 34. Stability of catalyst. (a) TEM and (b) HRTEM images of pCOL-Pd/AuNC before and after catalysis. (c) ICP-AES data of Au and Pd weight % before and after the reaction.

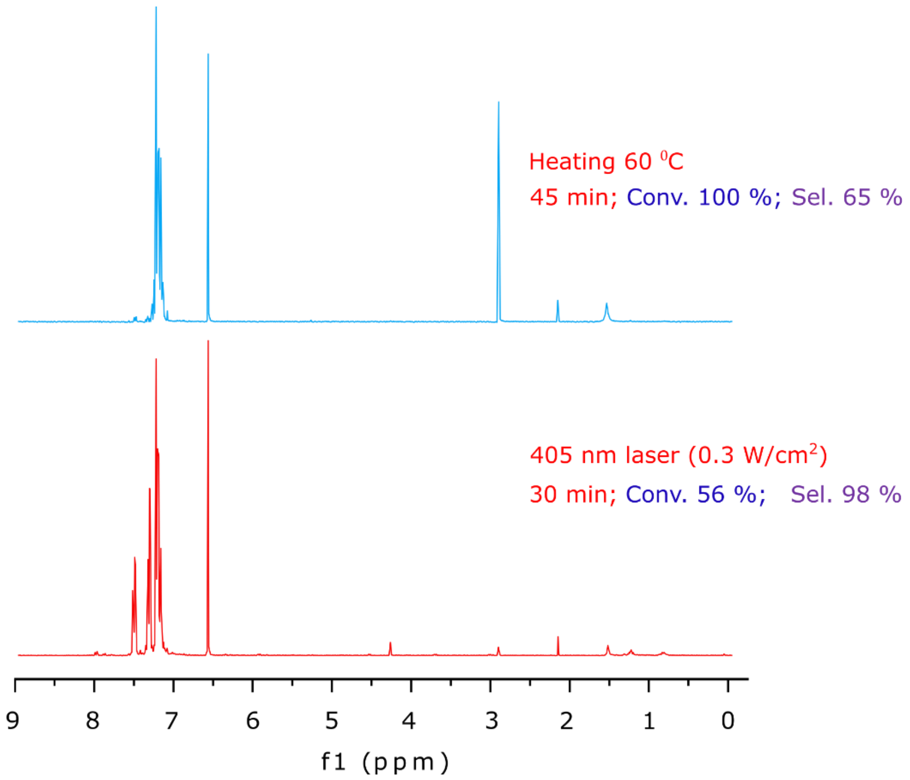

Supplementary Figure 35. Controlled experiments for hydrogenation of diphenylacetylene. ^{1}H NMR data for the hydrogenation of diphenylacetylene after externally elevating the temperature (60 °C) during laser-irradiation (405 nm; 0.3 W/cm^2) using pCOL-Pd/AuNC@*h*-SiO_2.

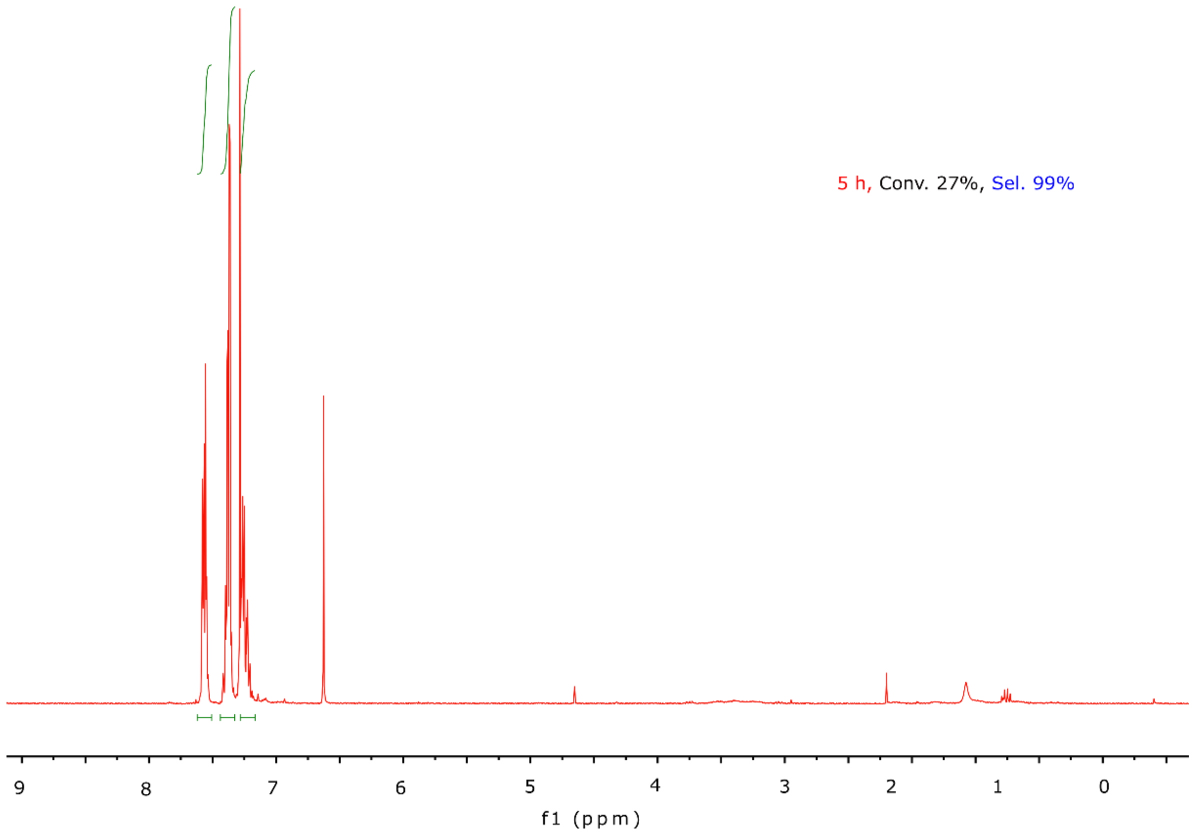

Supplementary Figure 36. Controlled catalytic reaction with bulk-COF@Pd/AuNC. ^{1}H NMR data for the hydrogenation of diphenylacetylene using bulk-COF@Pd/AuNC under laser-irradiation (405 nm; 0.3 W/cm^{2}).

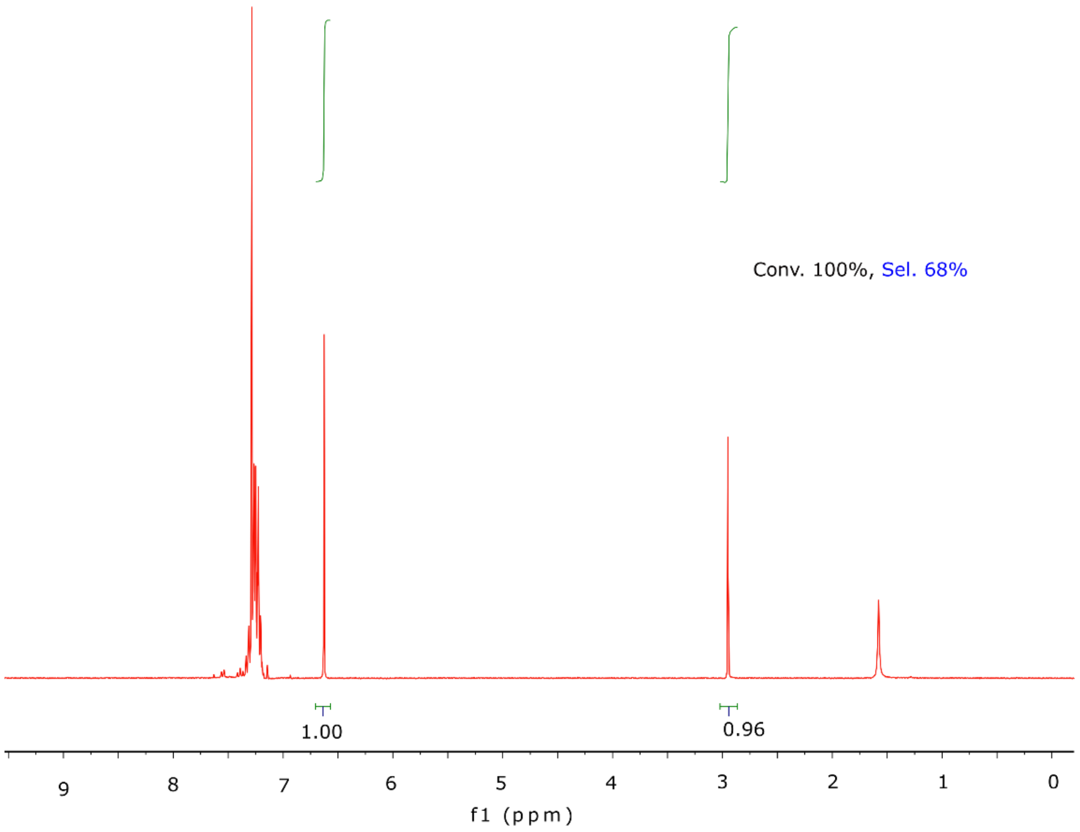

Supplementary Figure 37. Controlled catalytic reaction with TAE-Pd/AuNC@*h*-SiO_2. ^{1}H NMR data for the hydrogenation of diphenylacetylene using TAE-Pd/AuNC@*h*-SiO_2 under the exposure of 405 nm laser (0.3 W/cm^2).

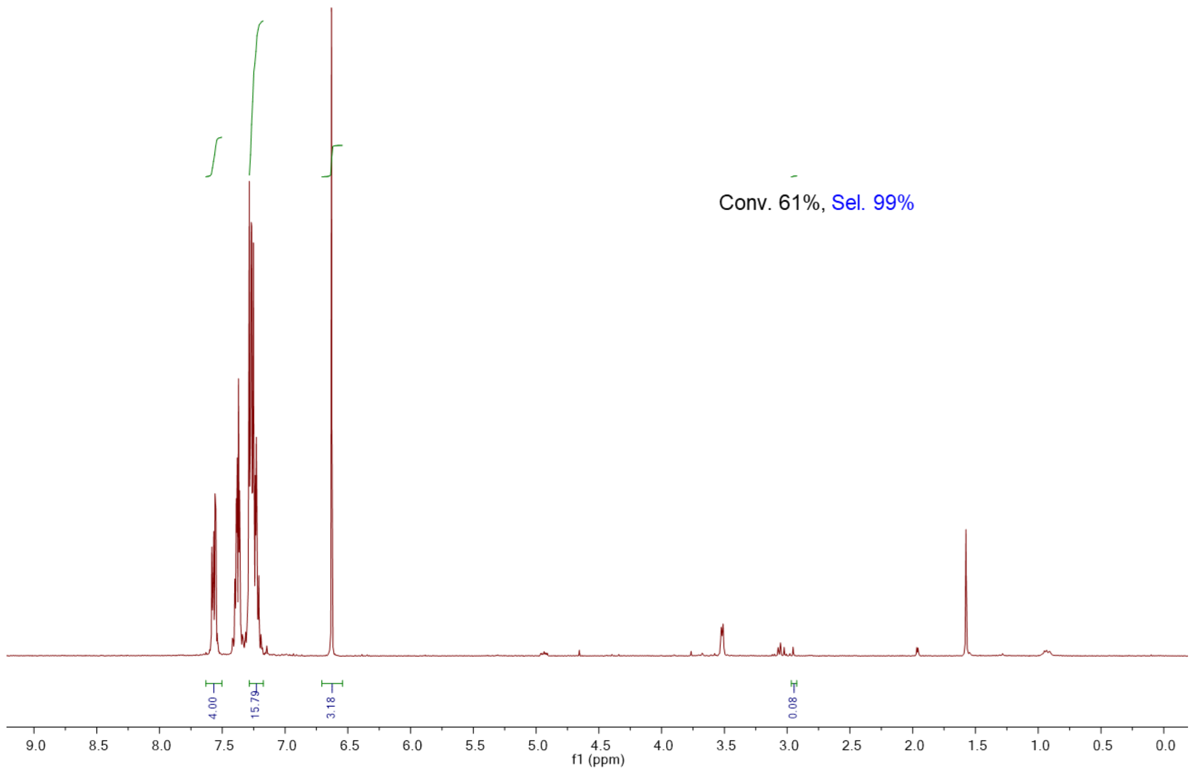

Supplementary Figure 38. Semi-hydrogenation of diphenylacetylene during the real-time SERS based catalysis. ^{1}H NMR spectrum of reaction solution showing the semi-hydrogenated product using pCOL-Pd/AuNC@*h*-SiO_2 during the real-time SERS study.

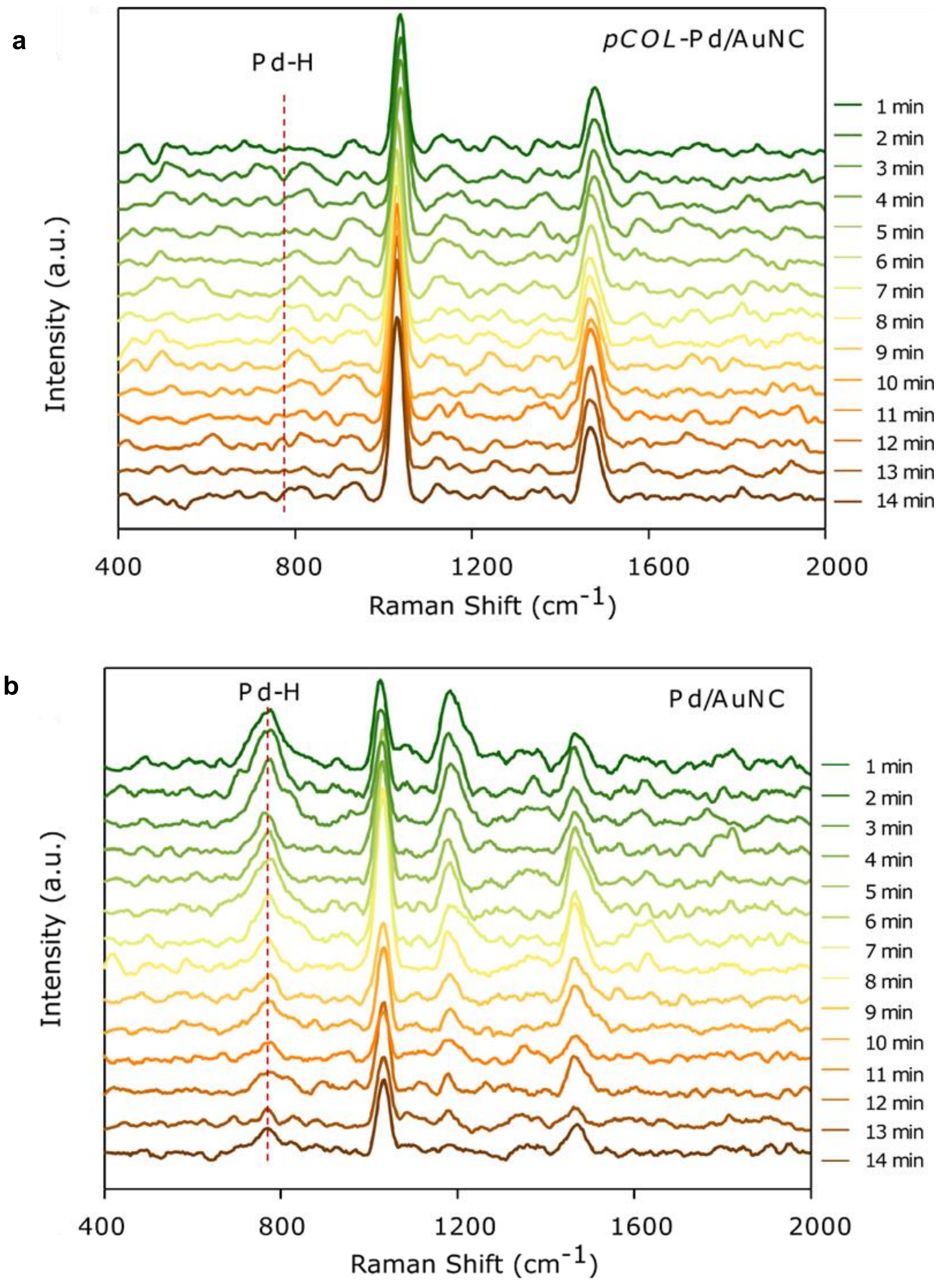

Supplementary Figure 39. In-situ Raman data of hydrogen desorption. Time dependent in-situ Raman signals corresponding to Pd-H bonding on (a) pCOL-Pd/AuNC (b) Pd/AuNC catalyst's surface.

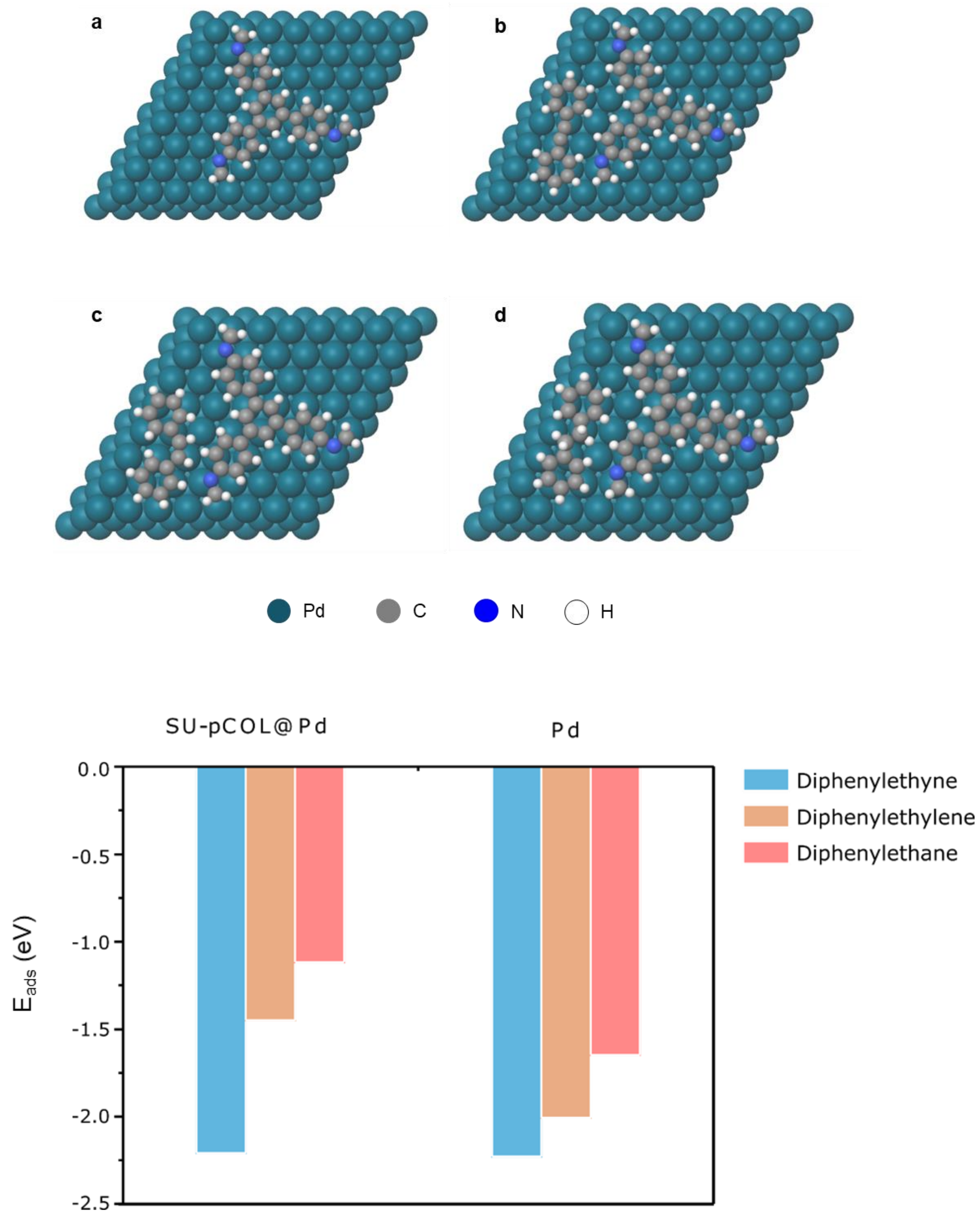

Supplementary Figure 40. Adsorption energy calculations and DFT models for interactions of reactants and products on catalytic surface. DFT optimized model structure of (a) Pd-surface capped with SU-pCOL. Adsorption of (b) alkyne (diphenylacetylene), (c) alkene (cis-stilbene), (d) alkane (diphenylethane) on SU-pCOL@Pd surface. And, the box below represents the corresponding adsorption energies of diphenylacetylene, cis-stilbene, and diphenylethane on SU-pCOL@Pd and Pd surface.

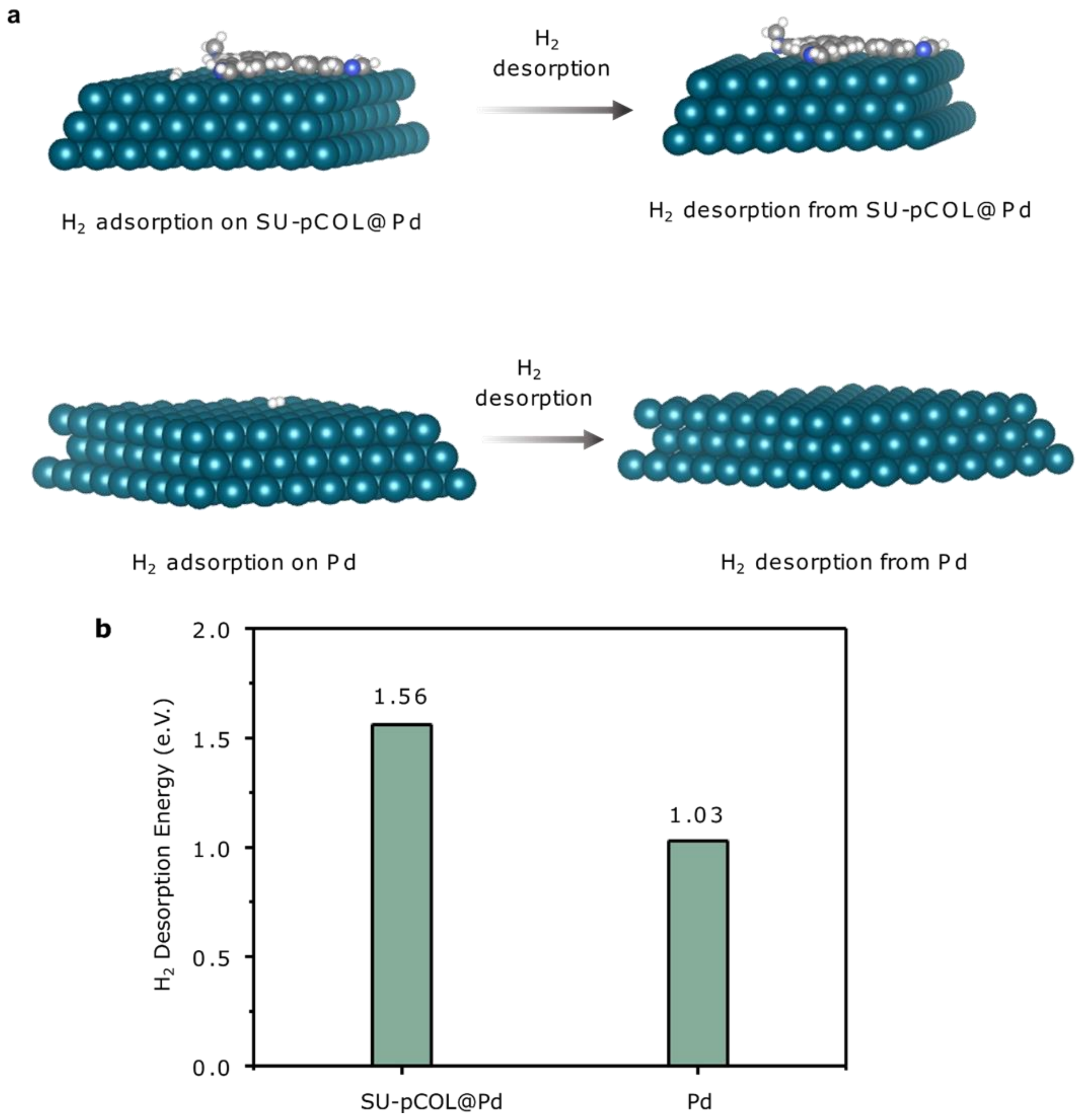

Supplementary Figure 41. Hydrogen desorption energy calculations and DFT models for interactions of hydrogen molecule on catalytic surface. DFT optimized model structure for hydrogen desorption phenomena from (a) SU-pCOL@Pd and Pd surface respectively. (b) Calculations for the desorption energy of hydrogen from SU-pCOL@Pd and Pd surface.

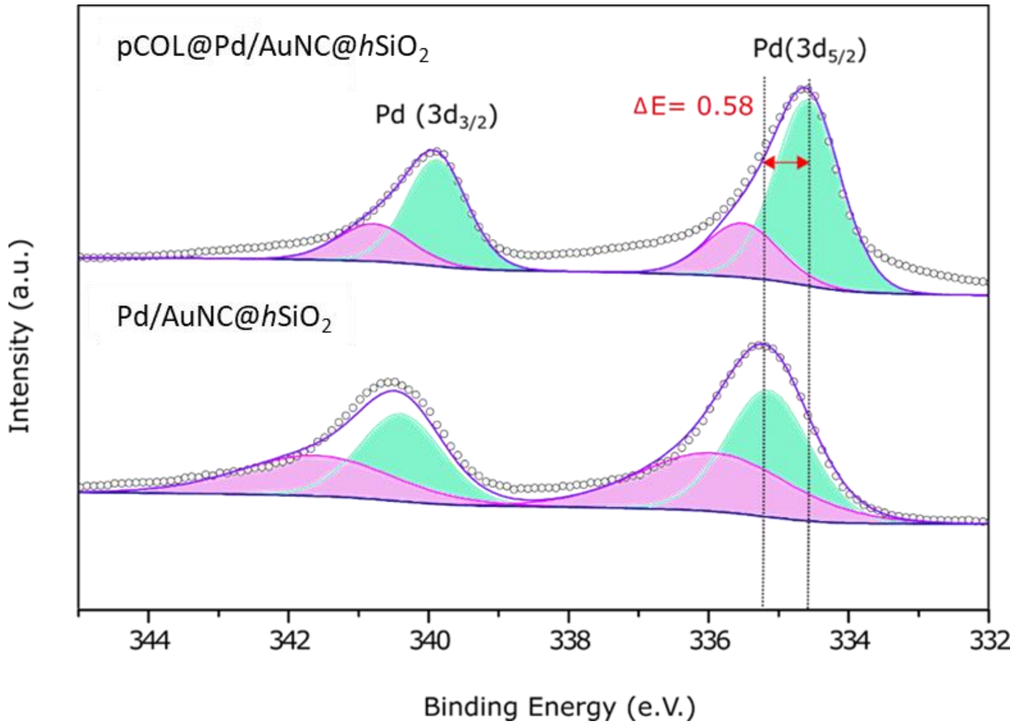

Supplementary Figure 42. XPS based characterization of Pd (3d) in pCOL-Pd/AuNC@*h*-SiO$_2$. Comparative XPS spectra of Pd (3d) in pCOL-Pd/AuNC@*h*-SiO$_2$ and Pd/AuNC@*h*-SiO$_2$

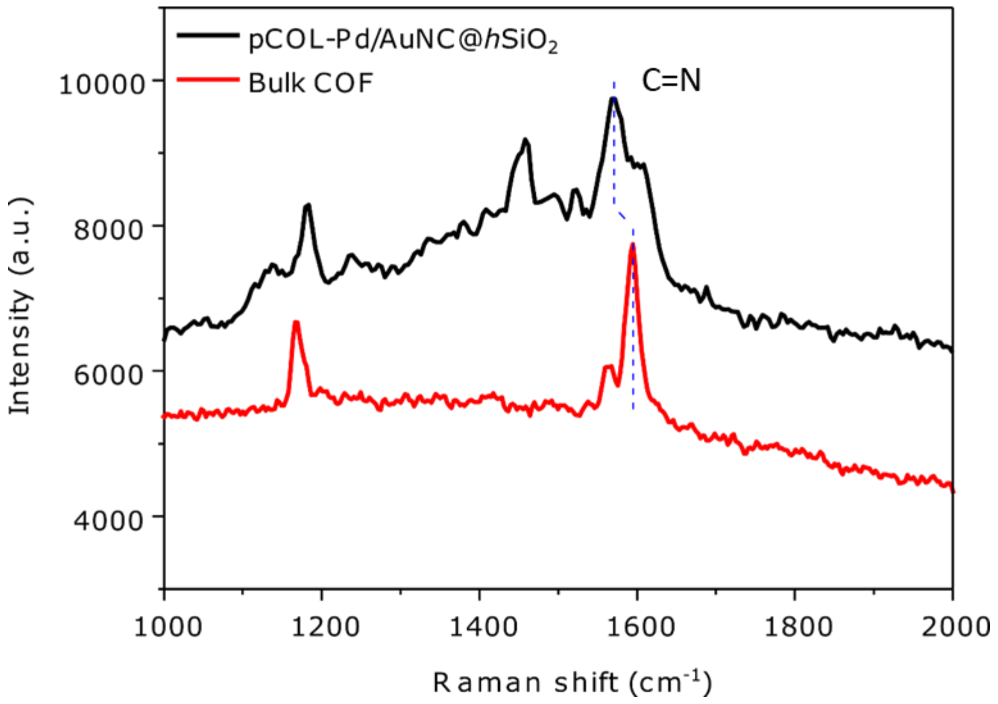

Supplementary Figure 43. Comparative Raman spectra of pCOL layer with bulk COF. Comparative Raman spectra of pCOL layer showing imine (C=N) peak shifted from bulk COF.

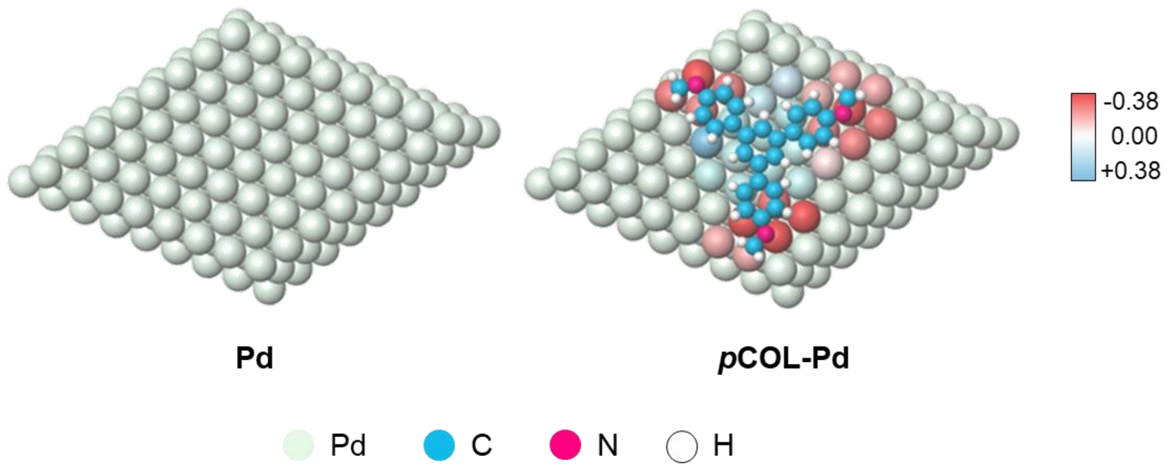

Supplementary Figure 44. Bader charge distribution models. Constructed models of Pd and SU-pCOL-Pd surfaces and their corresponding Bader charge analysis. The colour on Pd atoms indicate the Bader charge distribution on individual atoms.

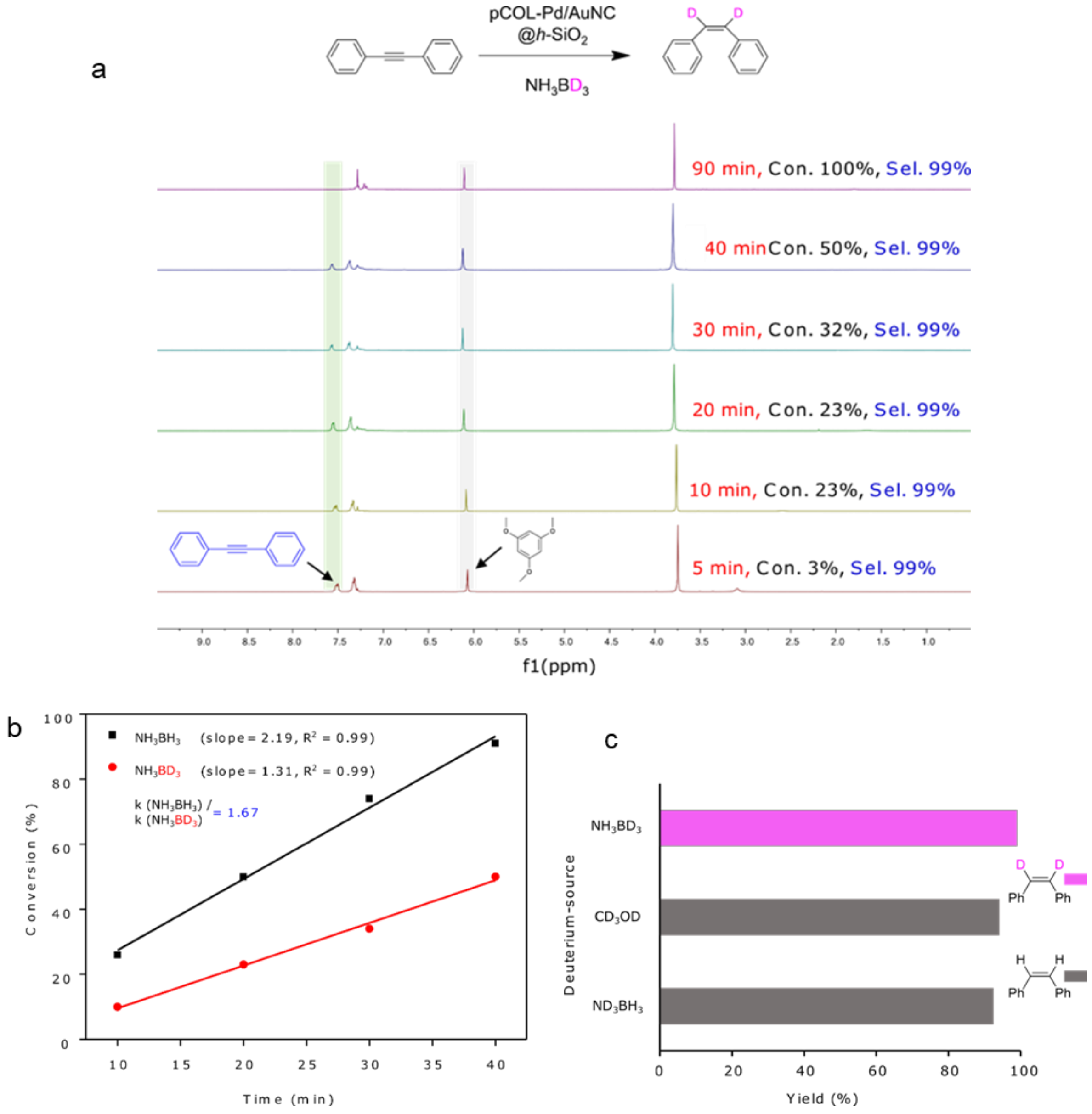

Supplementary Figure 45. Hydrogen/deuterium (H/D)-exchange study and kinetic isotope effects. (a) The time dependent stacked ^{1}H NMR data for semihydrogenation of diphenylacetylene using $NH_3.BD_3$ in presence of pCOL-Pd/AuNC @*h*-SiO_2 catalyst under 405 nm laser. (b) The linear data plot in the box shows typically moderate and comparable kinetic isotope effect: K_H/K_D = 1.67 using $NH_3.BD_3$. (c) Results of deuterium exchange reaction study using different deuterated reagent sources.

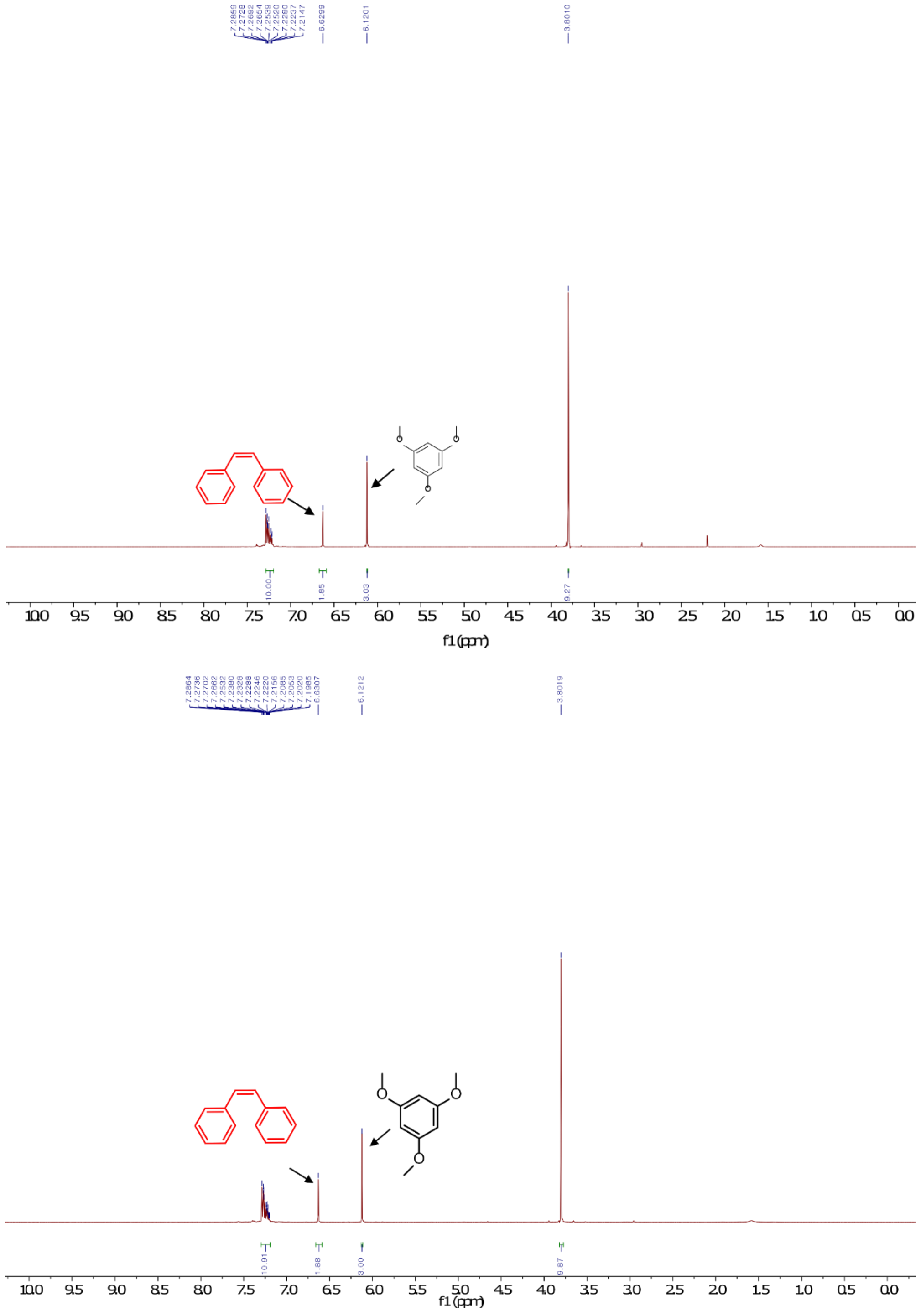

Supplementary Figure 46. Hydrogen/deuterium (H/D)-exchange study: Crude ^{1}H NMR data for semihydrogenation of diphenylacetylene with (a) ND_3BH_3 and (b) CD_3OD using pCOL-Pd/AuNC@*h*-SiO_2 as catalyst under 405 nm laser.

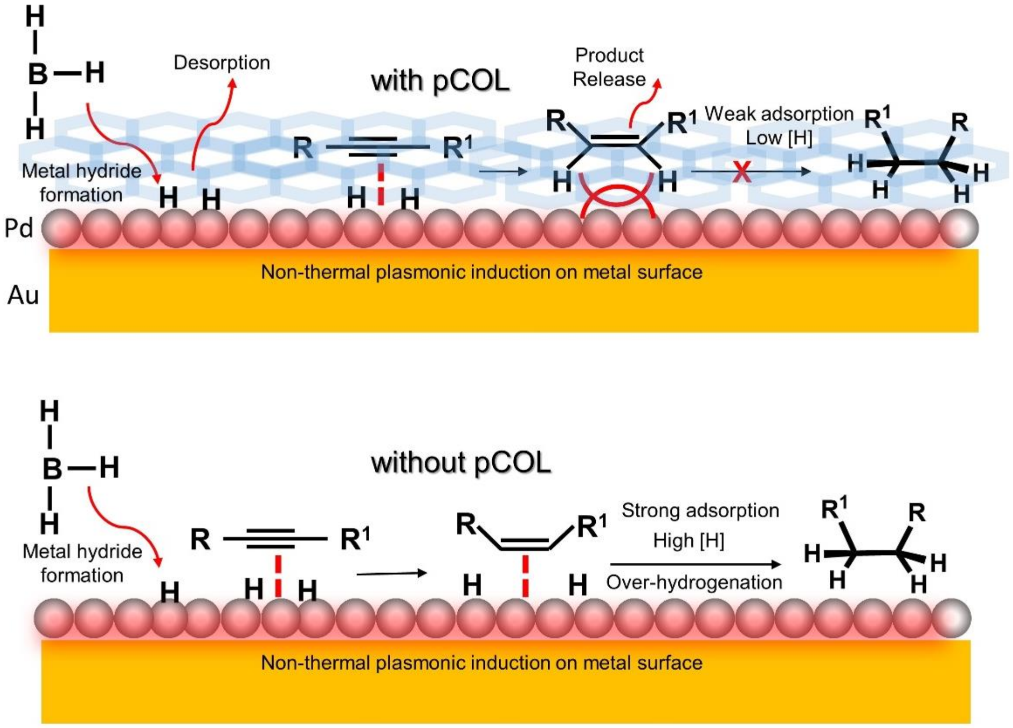

Supplementary Figure 47. Plausible mechanism for Semihydrogenation of alkyne. Schematic representations of the semihydrogenation mechanism involved on the (a) pCOL-Pd/AuNC and (b) Pd/AuNC surface.

4. Supplementary Synthesis Methods and Catalysis

4.1. (Z)-1,2-diphenylethene:

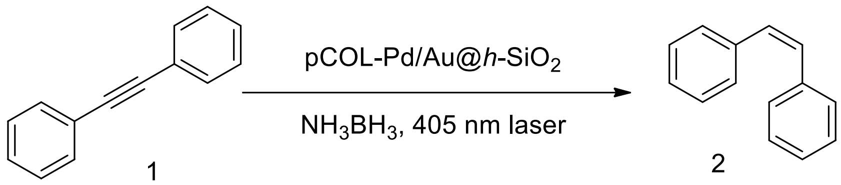

The conversion and selectivity was calculated by using the relative peak integration values.^{1}H NMR (300 MHz, Chloroform-*d*) δ 7.42 – 7.03 (m, 10H), 6.61 (s, 2H).

4.2. Synthesis of (4-(phenylethynyl)phenyl)methanethiol

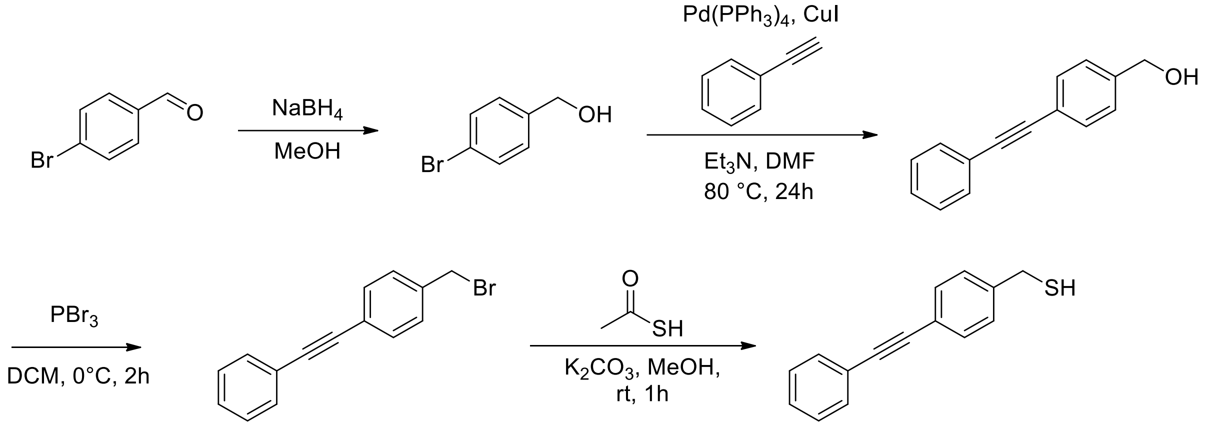

The stepwise synthesis of (4-(phenylethynyl)phenyl)methanethiol was adopted and modified from previously reported method.

4.3. 4-Bromobenzyl alcohol: 4-bromobenzaldehyde 1g (1 equiv.) dissolved in 10 mL methanol and cooled to 0 °C and added $NaBH_4$ (1 equiv.) in to it portion wise. After addition, the reaction mixture stirred for 30 min and concentrated under reduced pressure. The residue dissolved in ethyl acetate, washed with dilute HCl and then water. The organic layer dried over Na_2SO_4 and concentrated under reduced pressure to give the pure product as a white solid in a 99 % isolated yield.

4.4. (4-Phenylethynyl)benzyl alcohol : To a carefully degassed solution of 4-bromobenzyl alcohol 1 g (5.3 mmol), CuI 20.3 mg (0.11 mmol) and $Pd(PPh_3)_4$ 62 mg (0.053 mmol) in 10 mL of dry THF and 10 mL of dry triethylamine was added phenylacetylene 583 mmL (5.88 mmol). The reaction was stirred overnight at 60 °C under nitrogen atmosphere. After completion of the reaction, the mixture was added to 50 mL of iced water and the organic phase was recovered, dried over $MgSO_4$. The solvent was removed under reduced pressure to give the pure product as a white solid (1.07 g, 96 % yield).

4.5. (4-Phenylethynyl)benzyl bromide: (4-Phenylethynyl)benzyl alcohol 500 mg (2.4 mmol)

dissolved in 10 mL of dry DCM. The mixture then cooled to 0 °C and phosphorus tribromide added dropwise. Then the mixture stirred for 2 hours at 0 °C and the solvent removed under reduced pressure. The crude product purified by column chromatography to afford the product as a white solid (631 mg, 97 % yield).

4.6. (4-(phenylethynyl)phenyl)methanethiol: (4-Phenylethynyl)benzyl bromide 100 mg (0.37 mmol) and thioacetic acid 34 mg (0.44 mmol) were dissolved in 2 mL of methanol. Then added first portion of K_2CO_3 (66 mg, 0.6 mmol) and stirred for 30 min under N_2 atmosphere. Another portion of K_2CO_3 (66 mg, 0.6 mmol) was added to the reaction mixture and stirred for 1 h under nitrogen atmosphere. Then 0.5 ~ 0.1 mL of dil HCl solution was added to neutralize the reaction solution (to pH ~ 6). Methanol was then evaporated under reduced pressure and the residue was extracted with 2 X 2 mL of $CHCl_3$. The chloroform layer was washed with water, dried over anhydrous Na_2SO_4, and the solvent removed under reduced pressure to yield 99% of (4-(phenylethynyl)phenyl)methanethiol.

^{1}H NMR (300 MHz, Chloroform-d) δ 7.55 – 7.48 (m, 4H), 7.37 – 7.30 (m, 5H), 3.75 (d, J = 7.5 Hz, 2H), 1.77 (t, J = 7.6 Hz, 1H).

4.7. (Z)-1-methoxy-4-styrylbenzene (2a):

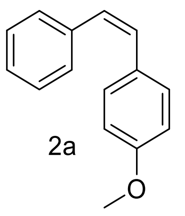

2a

The compound **2a** synthesized following the general procedure described for **2**.

Conversion 100%, Selectivity 98 %

^{1}H NMR (300 MHz, Chloroform-*d*) δ 7.36 – 7.16 (m, 7H), 6.78 (d, *J* = 8.9 Hz, 2H), 6.55 (d, *J* = 1.2 Hz, 2H), 3.81 (s, 3H).

4.8. (Z)-1-methyl-4-styrylbenzene (2b):

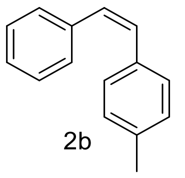

2b

The compound **2b** synthesized following the general procedure described for **2.**

Conversion 100%, Selectivity 99 %

^{1}H NMR (300 MHz, Chloroform-*d*) δ 7.33 – 7.18 (m, 5H), 7.15 (d, *J* = 8.1 Hz, 2H), 7.04 (d, *J* = 7.9 Hz, 2H), 6.56 (s, 2H), 2.32 (s, 3H).

4.9. (Z)-1-methoxy-4-(4-methylstyryl)benzene (2c):

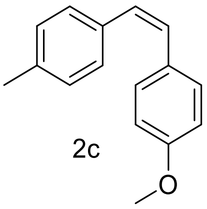

2c

The compound **2c** synthesized following the general procedure described for **2**.

Conversion 100% and Selectivity 99 %

^{1}H NMR (300 MHz, Chloroform-*d*) δ 7.20 (d, *J* = 8.8 Hz, 2H), 7.17 (d, *J* = 8.3 Hz, 2H), 7.04 (d, *J* = 7.8 Hz, 2H), 6.76 (d, *J* = 8.7 Hz, 2H), 6.48 (s, 2H), 3.79 (s, 3H), 2.32 (s, 3H).

4.10. (Z)-1-(tert-butyl)-4-styrylbenzene (2d):

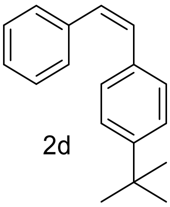

2d

The compound **2d** synthesized following the general procedure described for **2.**

Conversion 100%, Selectivity 99 %

^{1}H NMR (300 MHz, Chloroform-*d*) δ 7.34 – 7.20 (m, 9H), 6.59 (s, 2H), 1.32 (s, 9H).

4.11. (Z)-1-methyl-3-styrylbenzene (2e):

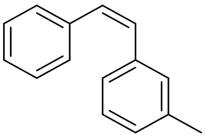

2e

The compound **2e** synthesized following the general procedure described for **2**.

Conversion 100%, Selectivity 99 %

^{1}H NMR (300 MHz, Chloroform-*d*) δ 7.28 – 7.18 (m, 5H), 7.14 – 7.00 (m, 4H), 6.58 (s, 2H), 2.27 (s, 3H).

4.12. (Z)-oct-1-en-1-ylbenzene (2f):

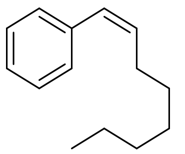

2f

The compound **2f** synthesized following the general procedure described for **2**.

Conversion 100%, Selectivity 99 %

^{1}H NMR (300 MHz, Chloroform-*d*) δ 7.36 – 7.19 (m, 5H), 6.41 (d, *J* = 11.7 Hz, 1H), 5.67 (dt, *J* = 11.3, 7.3 Hz, 1H), δ 2.37 – 2.29 (m, 2H), 1.48 – 1.40 (m, 2H), 1.33 – 1.21 (m, 6H), 0.88 (t, *J* = 6.7 Hz, 3H).

4.13. (Z)-3-phenylprop-2-en-1-ol (2g):

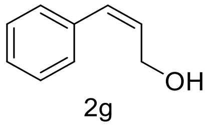

2g

The compound **2g** synthesized following the general procedure described for **2**.

Conversion 100%, Selectivity 99 %

^{1}H NMR (300 MHz, Chloroform-*d*) δ 7.38 – 7.33(m, 2H), 7.29 – 7.20 (m, 3H), 6.58 (d, *J* = 11.7 Hz, 1H), 5.88 (dt, *J* = 12.2, 6.4 Hz, 1H), 4.45 (d, *J* = 6.4 Hz, 2H), 1.56 (s, 1H).

4.14. (Z)-1-(tert-butyl)-4-(4-methylstyryl)benzene (2h):

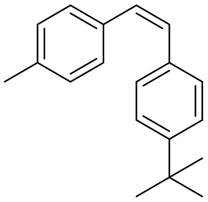

2h

The compound **2h** synthesized following the general procedure described for **2.**

Conversion 100%, Selectivity 99 %

^{1}H NMR (300 MHz, Chloroform-*d*) δ 7.23 – 7.18 (m, 6H), 7.05 (d, *J* = 7.9 Hz, 2H), 6.51 (s, 2H), 2.33 (s, 3H), 1.30 (s, 9H).

4.15. (Z)-1-methyl-3-(4-methylstyryl)benzene (2i):

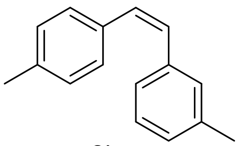

2i

The compound **2i** synthesized following the general procedure described for **2.**

Conversion 100%, Selectivity 99 %

^{1}H NMR (300 MHz, Chloroform-*d*) δ 7.15 (d, *J* = 8.1 Hz, 2H), 7.11 – 7.08(m, 3H), 7.05– 7.00 (m, 3H), 6.53 (s, 2H), 2.31 (s, 3H), 2.28 (s, 3H).

4.16. (Z)-1-methyl-4-(oct-1-en-1-yl)benzene (2j):

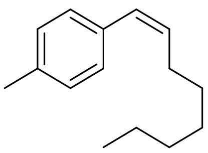

2j

The compound **2j** synthesized following the general procedure described for **2**.

Conversion 100 %, Selectivity 99 %

^{1}H NMR (300 MHz, Chloroform-*d*) δ 7.19 (d, *J* = 8.2 Hz, 2H), 7.14 (d, *J* = 8.2 Hz, 2H), 6.37 (d, *J* = 11.7 Hz, 1H), 5.62 (dt, *J* = 11.6, 7.2 Hz, 1H), 2.39 – 2.28 (m, 2H), 2.35 (s, 3H), 1.49 – 1.40 (m, 2H), 1.33 – 1.26 (m, 6H), 0.88 (t, *J* = 6.7 Hz, 3H).

4.17. (Z)-3-(p-tolyl)prop-2-en-1-ol (2k):

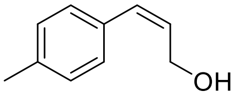

2k

The compound **2k** synthesized following the general procedure described for **2**.

Conversion 100%, Selectivity 99 %

^1H NMR (300 MHz, Chloroform-*d*) δ 7.16 (d, *J* = 8.1 Hz, 2H), 7.11 (d, *J* = 8.3 Hz, 2H), 6.54 (d, *J* = 11.7 Hz, 1H), 5.83 (dt, *J* = 11.7, 6.4 Hz, 1H), 4.44 (dd, *J* = 6.4, 1.7 Hz, 2H), 2.35 (s, 3H), 1.59 (s, 1H).

4.18. (Z)-1-(tert-butyl)-4-(4-methoxystyryl)benzene (2l):

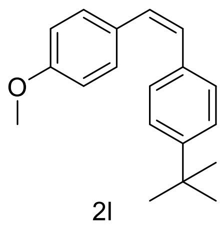

2l

The compound **2l** synthesized following the general procedure described for **2**.

Conversion 100%, Selectivity 99 %

^1H NMR (300 MHz, Chloroform-*d*) δ 7.25 – 7.21 (m, 6H), 6.78 (d, *J* = 8.7 Hz, 2H), 6.48 (s, 2H), 3.80 (s, 3H), 1.30 (s, 9H).

4.19. (Z)-1-(4-methoxystyryl)-3-methylbenzene (2m):

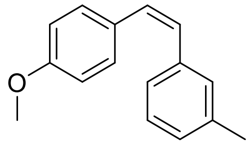

2m

The compound **2m** synthesized following the general procedure described for **2.**

Conversion 100%, Selectivity 99 %

^1H NMR (300 MHz, Chloroform-*d*) δ 7.22 (d, *J* = 8.7 Hz, 2H), 7.15 – 7.08 (m, 3H), 7.05 – 7.02 (m, 1H), 6.78 (d, *J* = 8.8 Hz, 2H), 6.52 (d, *J* = 1.7 Hz, 2H), 3.81 (s, 3H), 2.30 (s, 3H).

4.20. (Z)-3-(4-methoxyphenyl)prop-2-en-1-ol (2n):

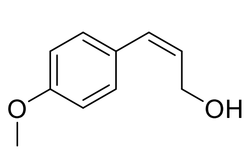

2n

The compound **2n** synthesized following the general procedure described for **2.**

Conversion 100%, Selectivity 99 %

^{1}H NMR (300 MHz, Chloroform-*d*) δ 7.16 (d, *J* = 8.7 Hz, 2H), 6.88 (d, *J* = 8.7 Hz, 2H), 6.51 (d, *J* = 11.7 Hz, 1H), 5.78 (dt, *J* = 11.6, 6.4 Hz, 1H), 4.44 (d, *J* = 6.8 Hz, 2H), 3.82 (s, 3H), 1.51 (s, 1H).

4.21. 1-methoxy-4-vinylbenzene (2o):

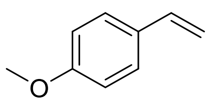

2o

The compound **2o** synthesized following the general procedure described for **2**.

Conversion 100%, Selectivity 99 %

^{1}H NMR (300 MHz, Chloroform-*d*) δ 7.35 (d, *J* = 8.7 Hz, 2H), 6.86 (d, *J* = 8.7 Hz, 2H), 6.66 (dd, *J* = 17.6, 10.9 Hz, 1H), 5.61 (dd, *J* = 17.6, 1.0 Hz, 1H), 5.12 (dd, *J* = 10.9, 1.0 Hz, 1H), 3.81 (s, 3H).

4.22. 1-(tert-butyl)-4-vinylbenzene (2p) :

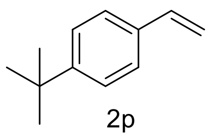

2p

The compound **2p** synthesized following the general procedure described for **2.**

Conversion 100%, Selectivity 98 %

^{1}H NMR (300 MHz, Chloroform-*d*) δ 7.35 (s, 4H), 6.70 (dd, *J* = 17.6, 10.9 Hz, 1H), 5.71 (dd, *J* = 17.6, 1.0 Hz, 1H), 5.19 (dd, *J* = 10.9, 1.0 Hz, 1H), 1.32 (s, 9H).

4.23. 1-vinylpyrene (2q):

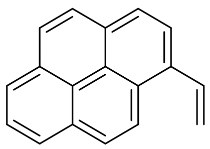

2q

The compound **2q** synthesized following the general procedure described for **2**.

Conversion 100%, Selectivity 89 %

^{1}H NMR (300 MHz, Chloroform-*d*) δ 8.42 (d, *J* = 9.3 Hz, 1H), 8.25 – 8.13 (m, 5H), 8.09 – 8.00 (m, 3H), 7.83 (dd, *J* = 17.3, 11.0 Hz, 1H), 6.02 (dd, *J* = 17.3, 1.3 Hz, 1H), 5.64 (dd, *J* = 11.0, 1.3 Hz, 1H).

4.24. N-allylbenzamide (2r):

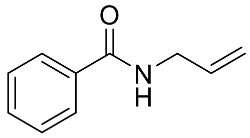

2r

The compound **2r** synthesized following the general procedure described for **2**.

Conversion 100%, Selectivity 78 %

^{1}H NMR (300 MHz, Chloroform-*d*) δ 7.80 – 7.74 (m, 2H)), 7.54 – 7.40 (m, 3), 6.17 (s, 1H), 5.95 (ddt, *J* = 17.1, 10.2, 5.7 Hz, 1H), 5.37 – 5.12 (m, 2H), 4.10 (tt, *J* = 5.7, 1.5 Hz, 2H).

4.25. N-(4-bromophenyl)pent-4-enamide (2s):

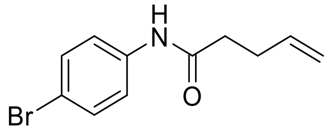

2s

The compound **2s** synthesized following the general procedure described for **2**

Conversion 100%, Selectivity 85 %

^{1}H NMR (300 MHz, Chloroform-*d*) δ 7.44 (s, 4H), 5.97– 5.84 (m, 1H), 5.19 – 5.07 (m, 2H), 2.49 (t, *J* = 1.7 Hz, 4H).

4.26. N-phenylpent-4-enamide (2t):

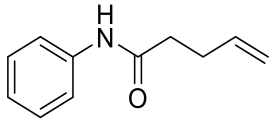

2t

The compound **2t** synthesized following the general procedure described for **2**.

Conversion 100%, Selectivity 98 %

^{1}H NMR (300 MHz, Chloroform-*d*) δ 7.54 – 7.47 (m, 2H), 7.31 (t, *J* = 7.9 Hz, 2H), 7.10 (t, *J* = 7.4 Hz, 1H), 5.95 – 4.82 (m, 1H), 5.16 – 4.04 (m, 2H), 2.47 (t, *J* = 1.9 Hz, 4H).

5. Supplementary ^{1}H NMR Spectra

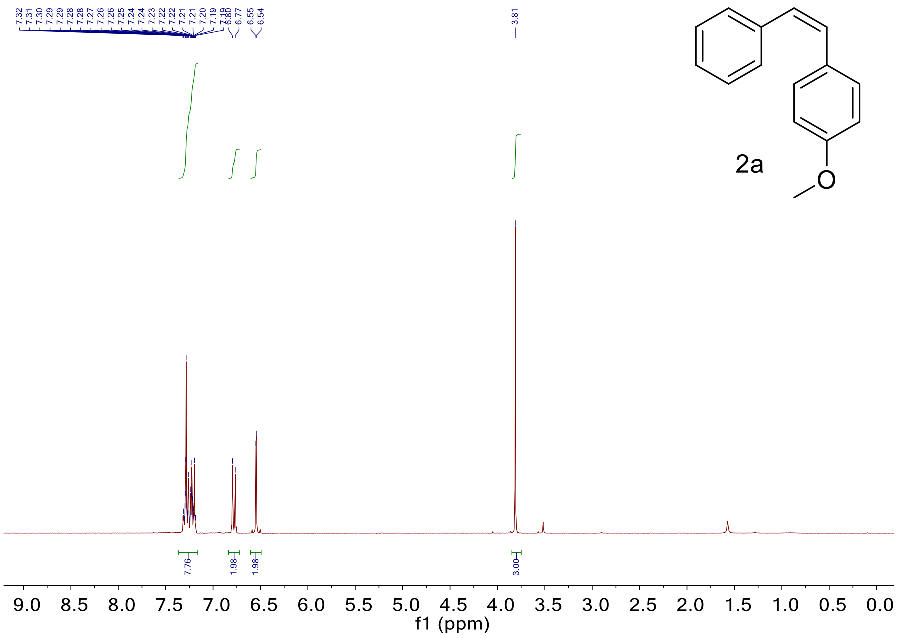

Supplementary Figure 48. ^{1}H NMR spectra of crude product **2a** in $CDCl_3$

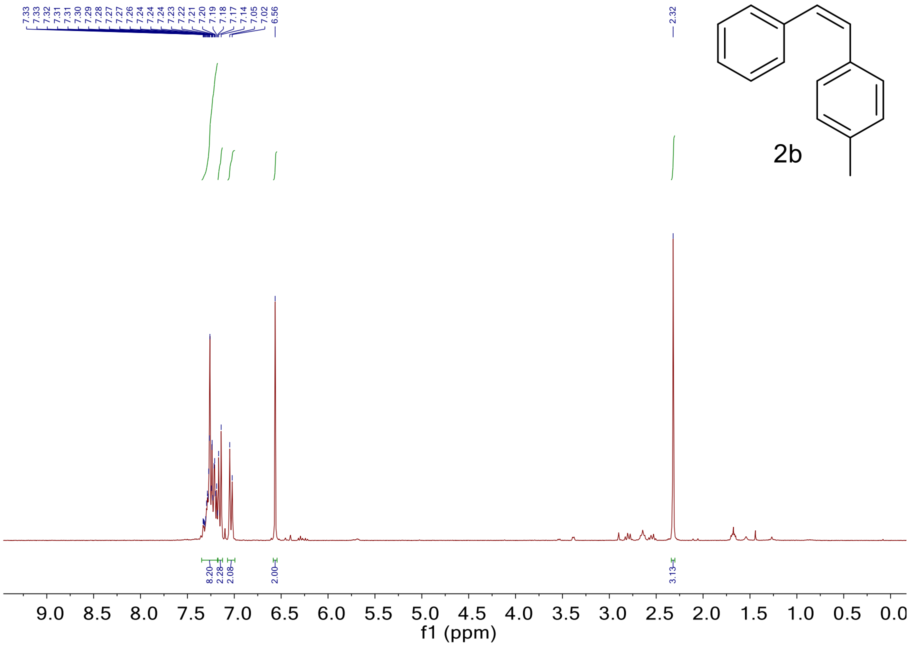

Supplementary Figure 49. ^{1}H NMR spectra of crude product **2b** in $CDCl_3$

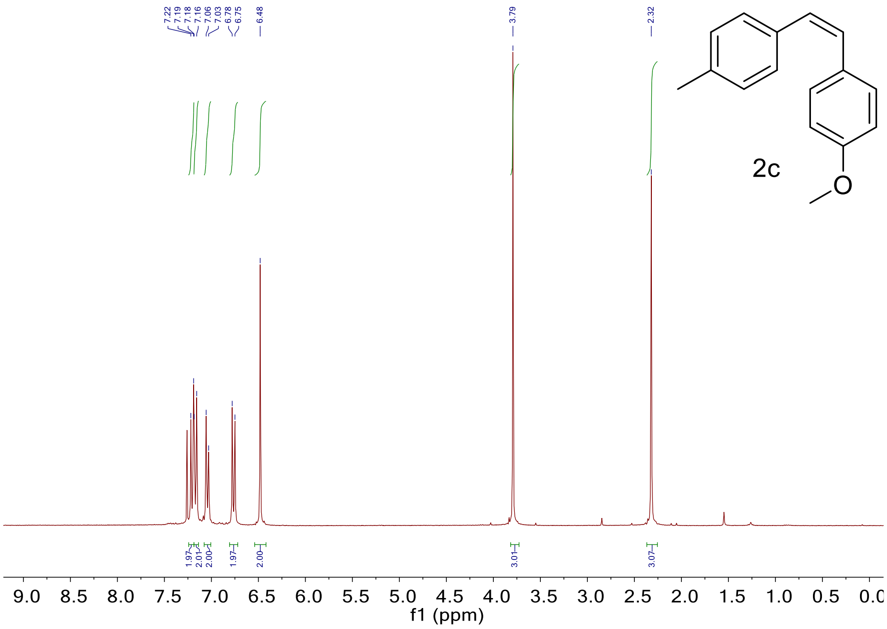

Supplementary Figure 50. ^{1}H NMR spectra of crude product **2c** in $CDCl_3$

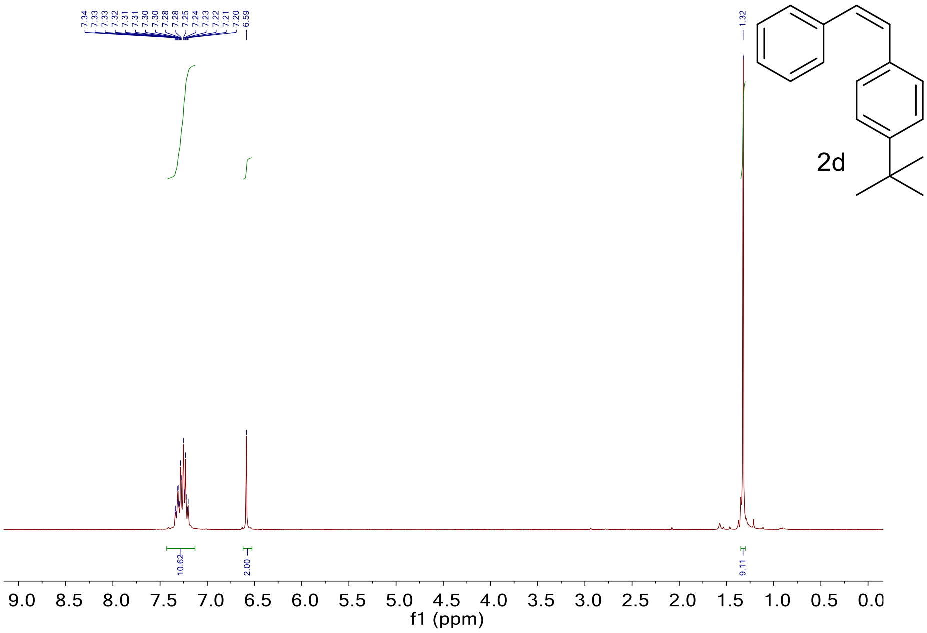

Supplementary Figure 51. ^{1}H NMR spectra of crude product **2d** in $CDCl_3$

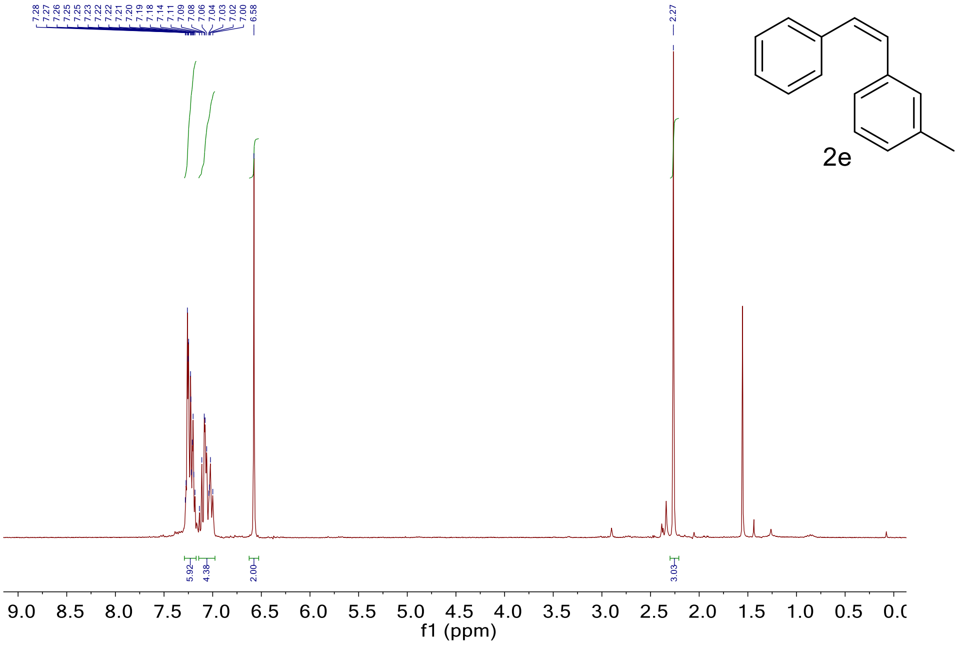

Supplementary Figure 52. ^{1}H NMR spectra of crude product **2e** in $CDCl_3$

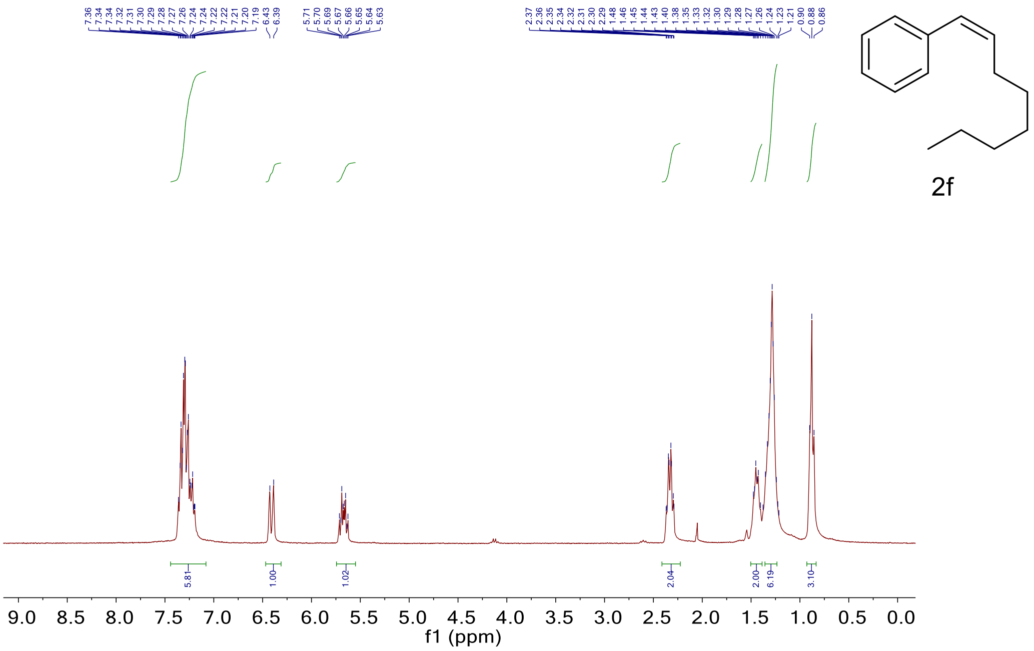

Supplementary Figure 53. ^{1}H NMR spectra of crude product **2f** in $CDCl_3$

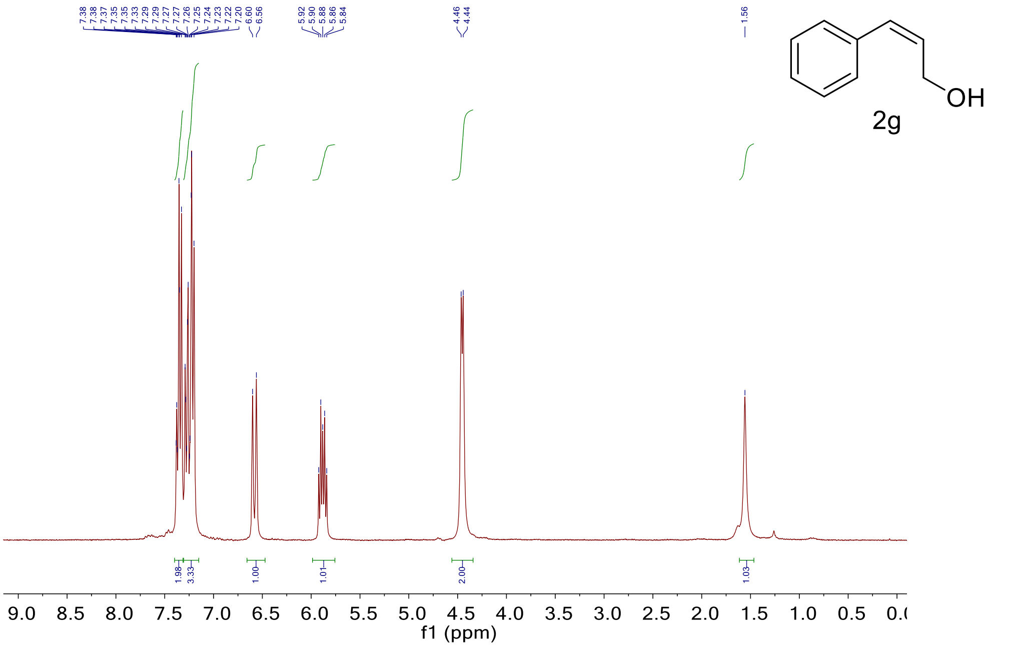

Supplementary Figure 54. ^{1}H NMR spectra of crude product **2g** in $CDCl_3$

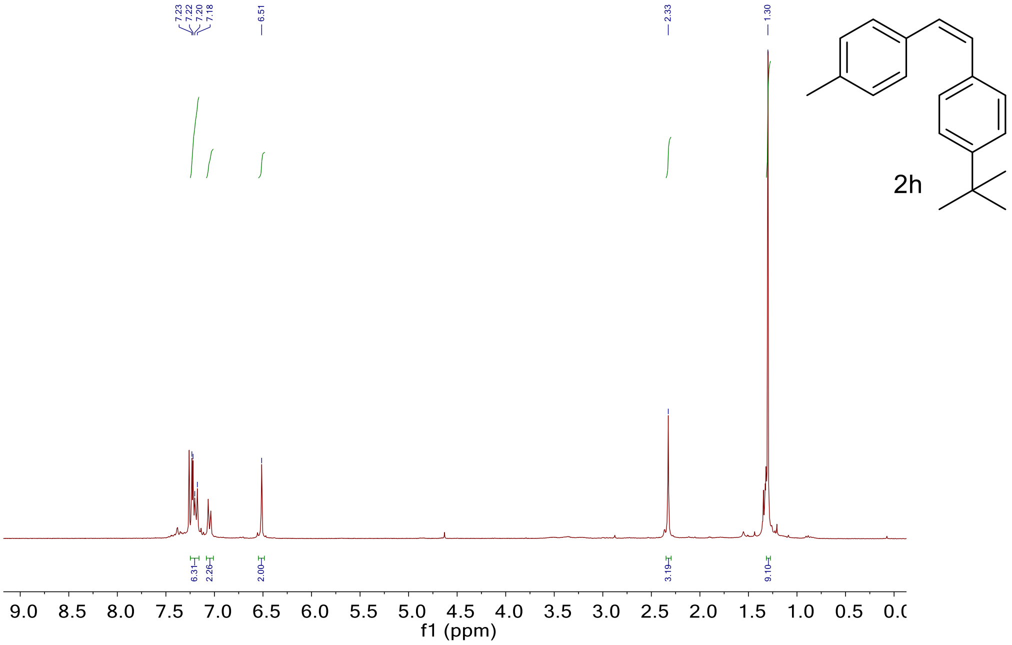

Supplementary Figure 55. ^{1}H NMR spectra of crude product **2h** in $CDCl_3$

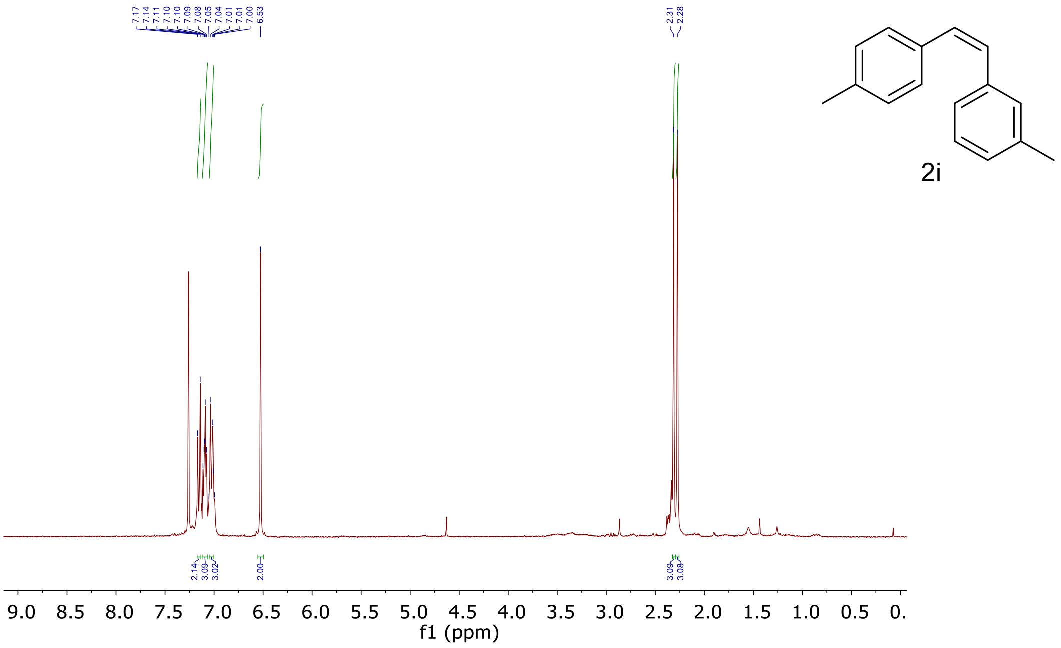

Supplementary Figure 56. ^{1}H NMR spectra of crude product **2i** in $CDCl_3$

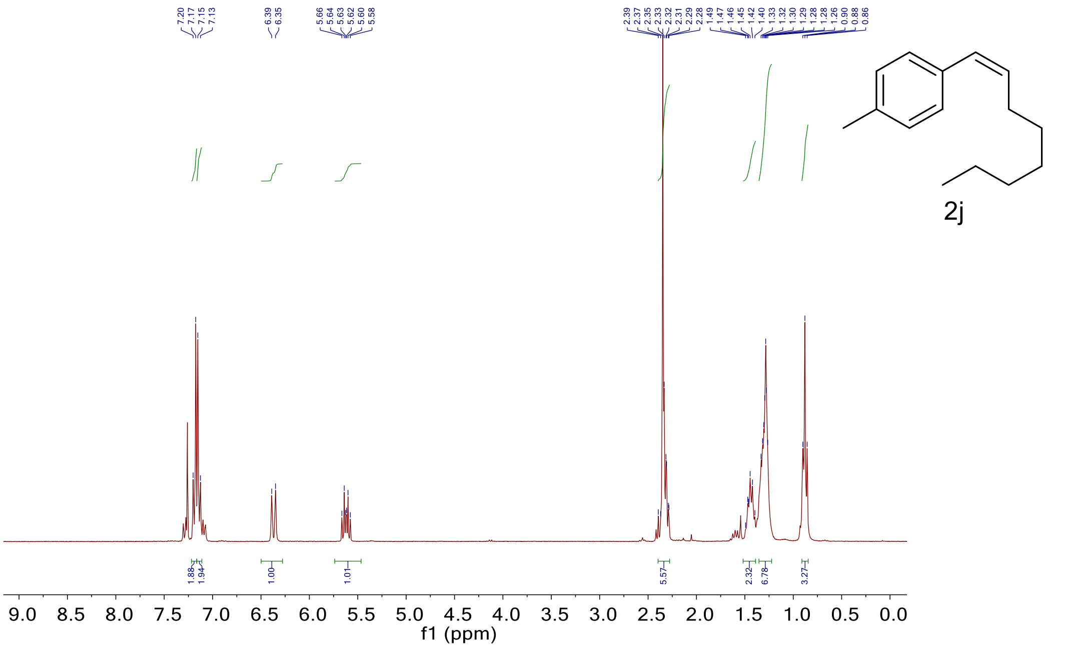

Supplementary Figure 57. ^{1}H NMR spectra of crude product **2j** in $CDCl_3$

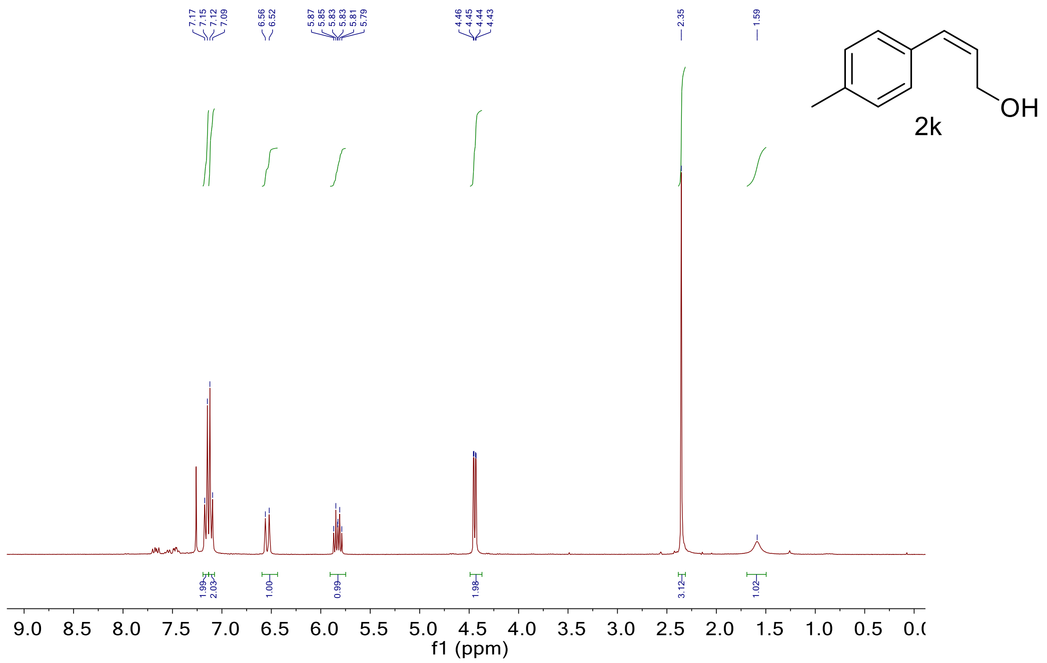

Supplementary Figure 58. ^{1}H NMR spectra of crude product **2k** in $CDCl_3$

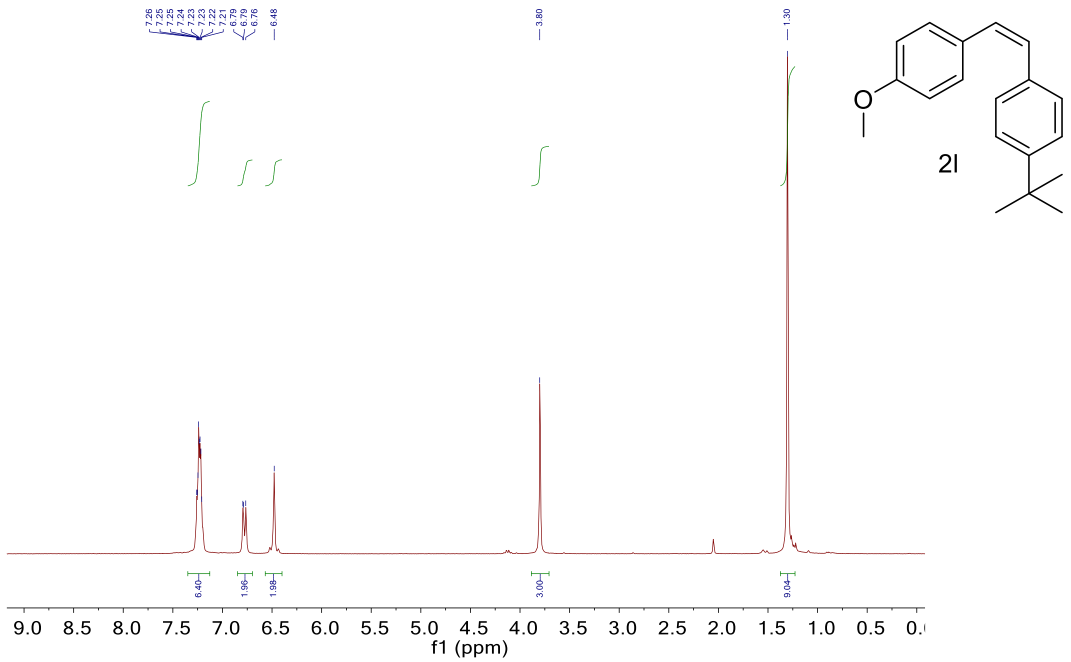

Supplementary Figure 59. ^{1}H NMR spectra of crude product **2l** in $CDCl_3$

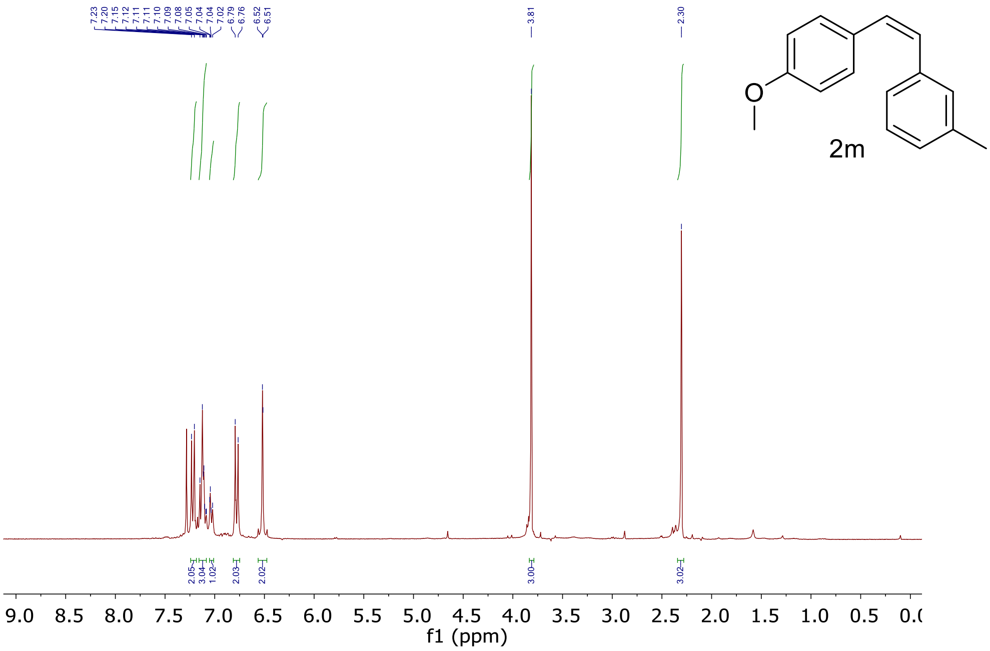

Supplementary Figure 60. ^{1}H NMR spectra of crude product **2m** in $CDCl_3$

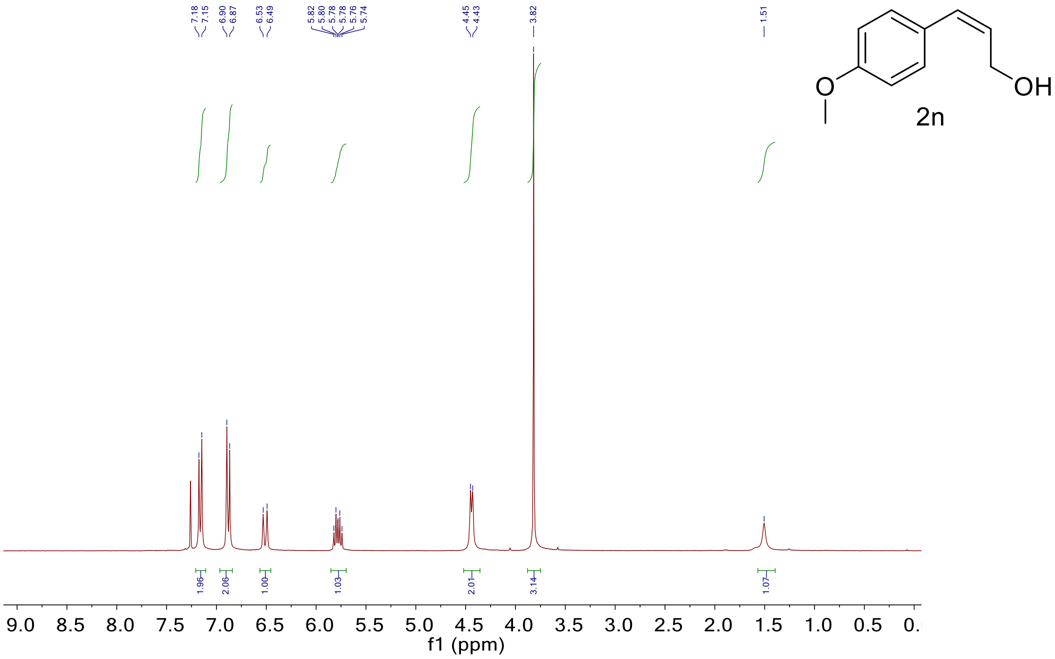

Supplementary Figure 61. ^{1}H NMR spectra of crude product **2n** in $CDCl_3$

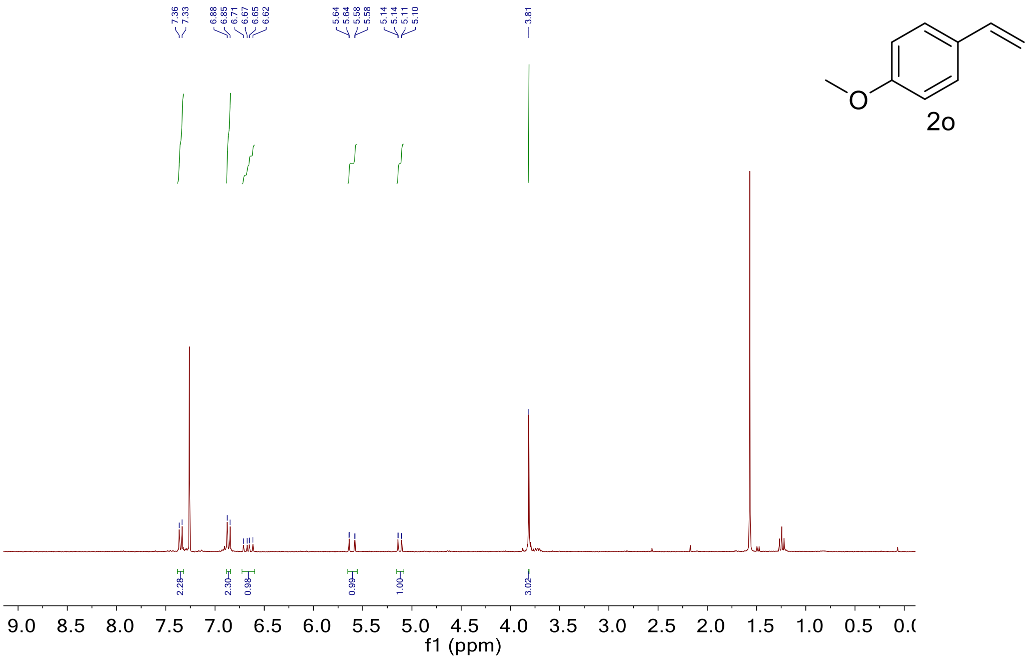

Supplementary Figure 62. ^{1}H NMR spectra of crude product **2o** in $CDCl_3$

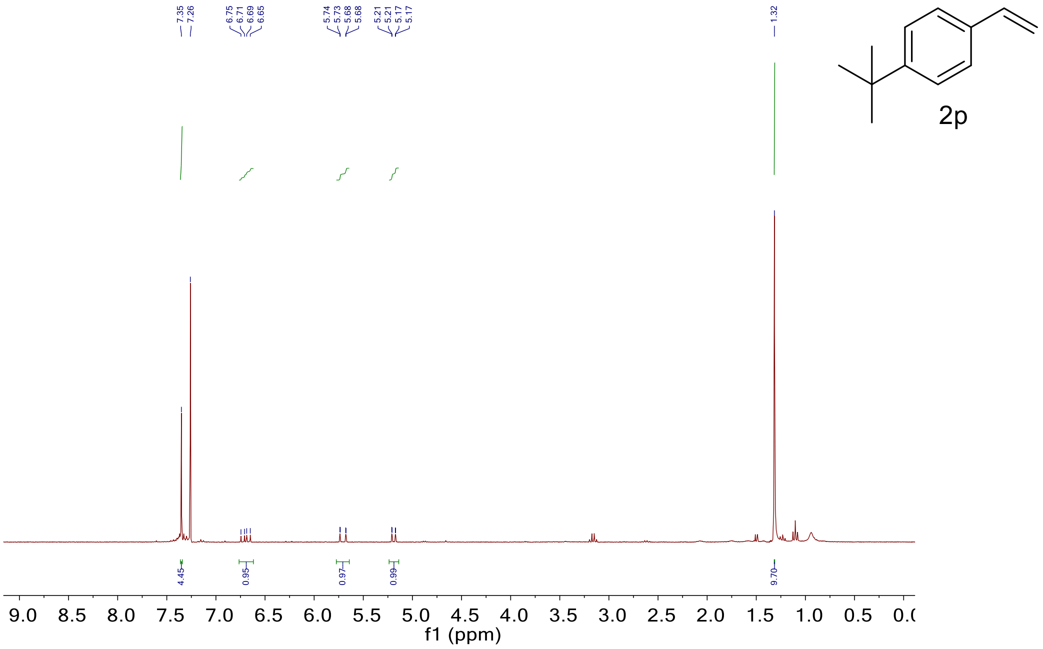

Supplementary Figure 63. ^{1}H NMR spectra of crude product **2p** in $CDCl_3$

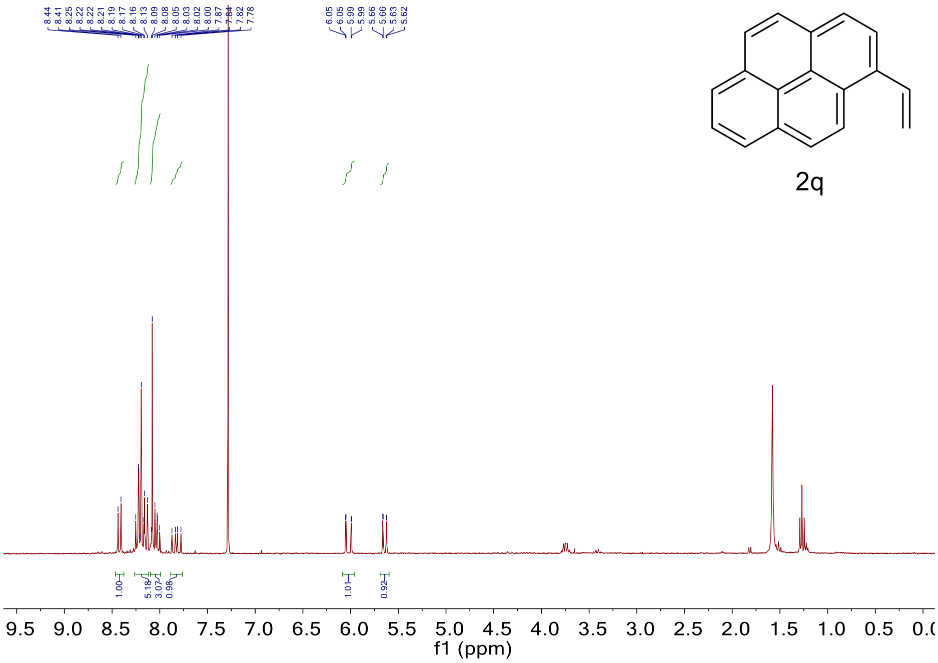

Supplementary Figure 64. ^{1}H NMR spectra of crude product **2q** in $CDCl_3$

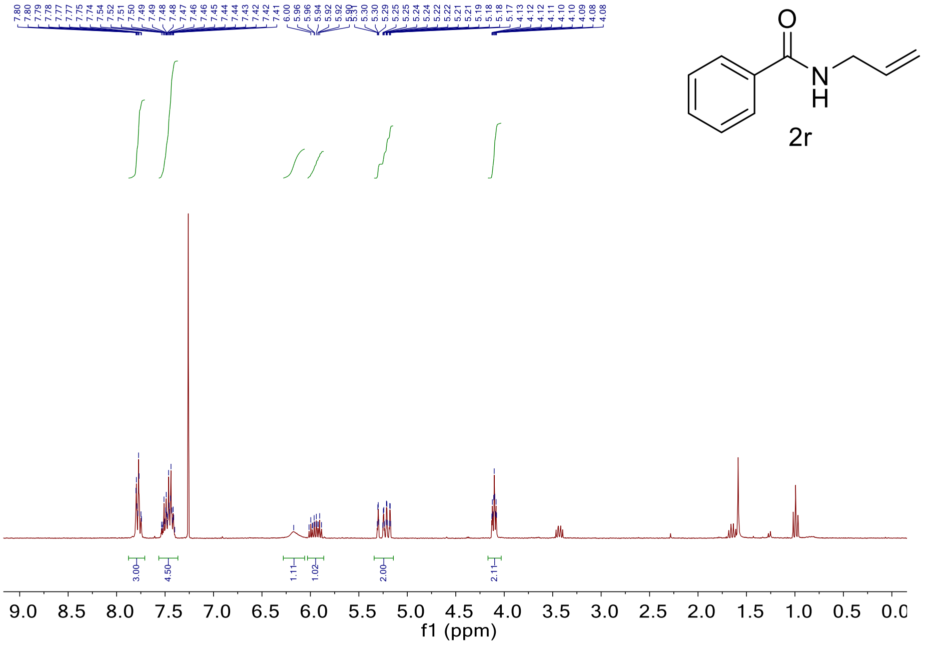

Supplementary Figure 65. ^{1}H NMR spectra of crude product **2r** in $CDCl_3$

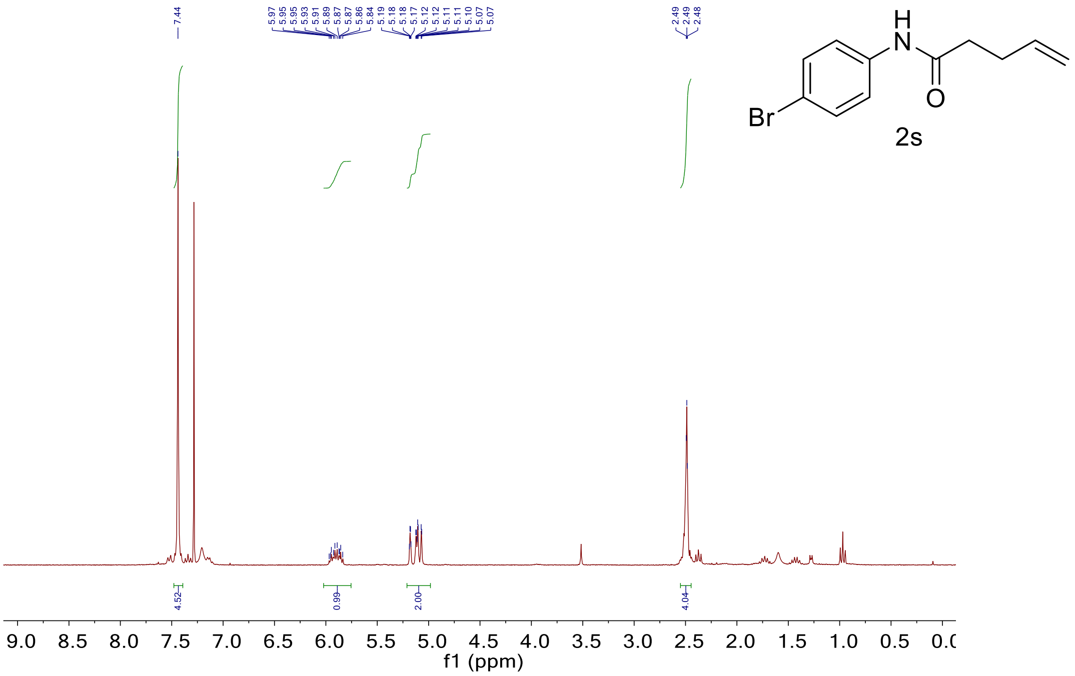

Supplementary Figure 66. ^{1}H NMR spectra of crude product **2s** in $CDCl_3$

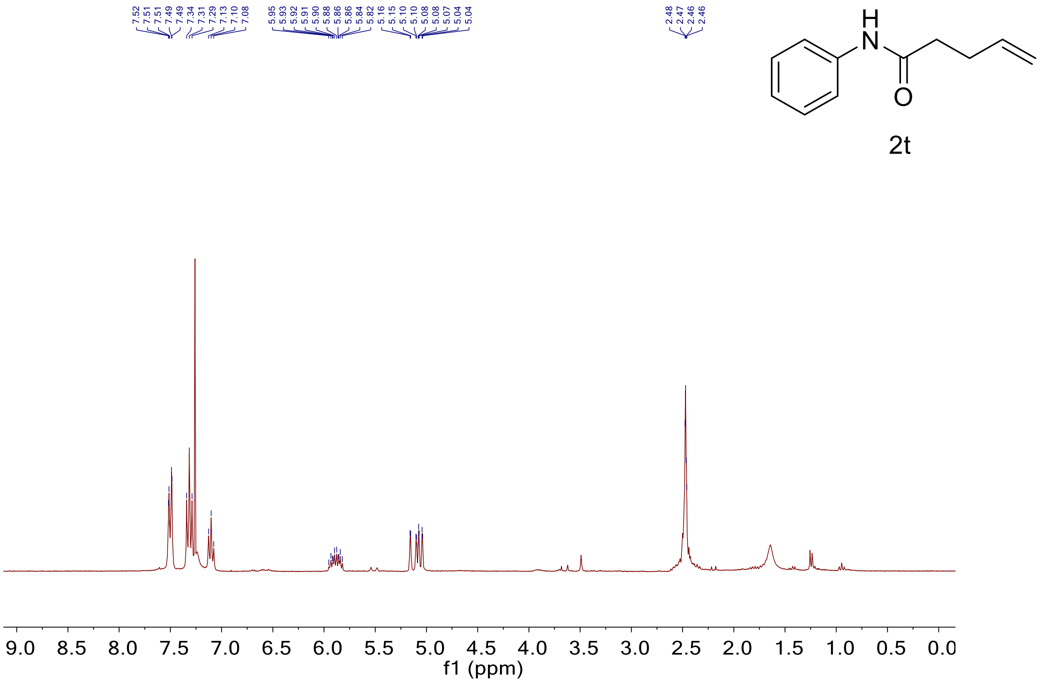

Supplementary Figure 67. ^{1}H NMR spectra of crude product **2t** in $CDCl_3$

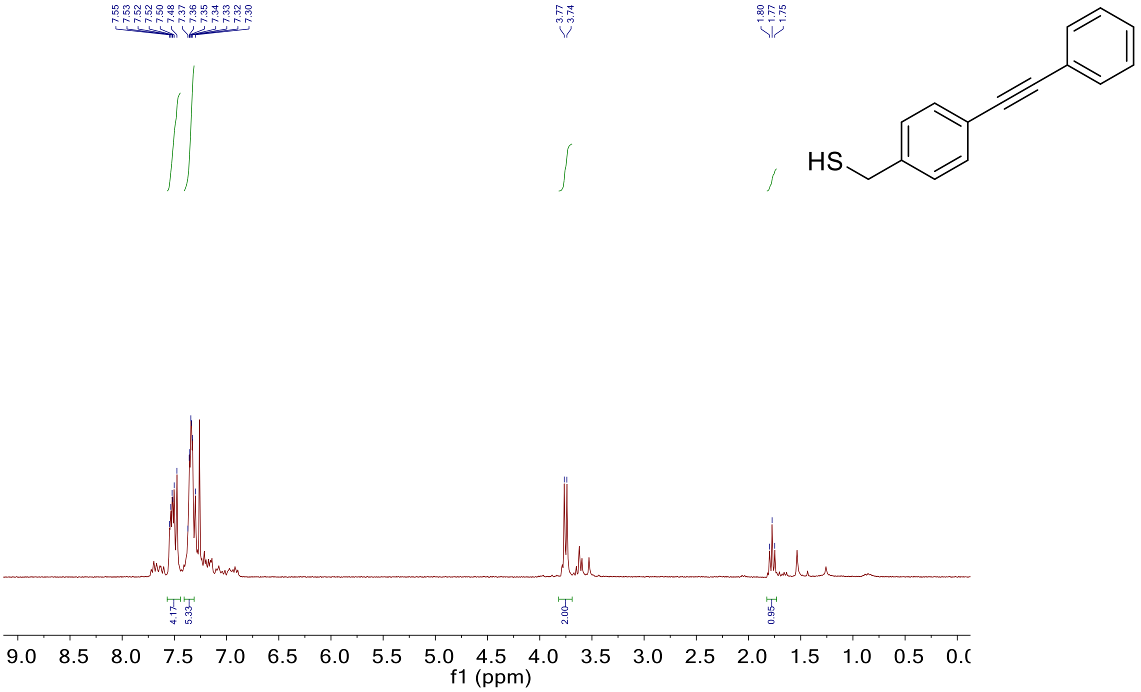

Supplementary Figure 68. ^{1}H NMR spectra of crude product (4-(phenylethynyl)phenyl)methanethiol in $CDCl_3$

SN	Name of catalyst	TOF (min^{-1})	conditions	H-Source	Time (min)	Con./Sel. (%)	Ref.
1	pCOL-Pd/AuNCs @*h*-SiO_2	63	RT; 1 atm	$NH_3.BH_3$	60	100/99	This work
2	PdCu@Cu_2O	0.625	30 °C; 0.1 MPa	H_2	800	99/95	8
3	Pd-In NPs	6.66	80 °C; 10 bar	H_2	15	100/85	9
4	Ni-Y Complex	0.025	70 °C; 4 atm	H_2	1440	99/99	10
5	Pd/NHC	0.21	145 °C;	H_2	120	99/98	11
6	CuPd@ZIF-8	14	RT; 1 atm	$NH_3.BH_3$	5	7/99	12
7	Pd+PEI@HSS	0.55	30 °C; 1atm	H_2	360	99/95	13
8	Pd_3Pb CNCs	0.23	50 °C; 2 atm	H_2	300	100/92	14
9	Mn Complex	0.083	60-70 °C;	$NH_3.BH_3$	1200	100/99	15
10	Co(II)/$NaBH_4$	0.069	RT; 3 bar	H_2	1440	100/84	16
11	$NiCl_2$	49	RT	$NaBH_4$	5	98/94	17
12	PdNPore	0.019	RT; 1atm	H_2	1080	95/97	18
13	Co/phen@SiO_2-800	0.11	120 °C; 30 bar	H_2	900	99/90	19
14	Pd^0-AmP-HSN	1.77	RT;1atm	H_2	210	93/89	20
15	Pd@Ag-0.20	1.26	RT; 1 atm	H_2	240	99/99	21
16	AuNPore	0.083	70 °C;	HCOOH	240	100/99	22
17	Pd/IL/MOF	8.6	30 °C; 1atm	H_2	180	99/99	23
18	Pd–Pb alloy NCs	0.91	RT; 1 atm	H_2	210	96/99	24
19	Pd/SBA-gt-PEI.	21.7	RT; 0.1 MPa	H_2	45	98/90	25

Supplementary Table 1. Table contains catalytic efficiencies and reaction conditions of the reported catalysts compared to pCOL-Pd/AuNC@*h*-SiO_2 for the semihydrogenation of alkyne (diphenylacetylene).

6. Supplementary References

1. Lee, J. E., Ahn, G., Shim, J., Lee, Y. S. & Ryu, S. Optical separation of mechanical strain from charge doping in graphene. *Nat Commun* **3**, 1024 (2012).
2. Liu, K.-K., Tadepalli, S., Tian, L. & Singamaneni, S. Size-Dependent Surface Enhanced Raman Scattering Activity of Plasmonic Nanorattles. *Chem. Mater.* **27**, 5261–5270 (2015).
3. Park, J.-E., Lee, Y. & Nam, J.-M. Precisely Shaped, Uniformly Formed Gold Nanocubes with Ultrahigh Reproducibility in Single-Particle Scattering and Surface-Enhanced Raman Scattering. *Nano Lett.* **18**, 6475–6482 (2018).
4. Pozzi, E. A. *et al.* Evaluating Single-Molecule Stokes and Anti-Stokes SERS for Nanoscale Thermometry. *J. Phys. Chem. C* **119**, 21116–21124 (2015).
5. Parvanov, V. M. *et al.* Materials for hydrogen storage: structure and dynamics of borane ammonia complex. *Dalton Trans.* 4514–4522 (2008) doi:10.1039/B718138H.
6. Ramachandran, P. V. & Gagare, P. D. Preparation of Ammonia Borane in High Yield and Purity, Methanolysis, and Regeneration. *Inorg. Chem.* **46**, 7810–7817 (2007).
7. Lee, G. *et al.* Excited-state dynamics of 4-dimethylamino-4′-nitrobiphenyl confined in AOT reverse micelles. *Journal of Molecular Liquids* **305**, 112873 (2020).
8. Liu, K. *et al.* Atomic overlayer of permeable microporous cuprous oxide on palladium promotes hydrogenation catalysis. *Nat Commun* **13**, 2597 (2022).
9. Chen, S. *et al.* Pd–In intermetallic nanoparticles with high catalytic selectivity for liquid-phase semi-hydrogenation of diphenylacetylene. *Nanoscale* **14**, 17661–17669 (2022).
10. Ramirez, B. L. & Lu, C. C. Rare-Earth Supported Nickel Catalysts for Alkyne Semihydrogenation: Chemo- and Regioselectivity Impacted by the Lewis Acidity and Size of the Support. *J. Am. Chem. Soc.* **142**, 5396–5407 (2020).
11. Denisova, E. A. *et al.* "Hidden" Nanoscale Catalysis in Alkyne Hydrogenation with Well-Defined Molecular Pd/NHC Complexes. *ACS Catal.* **12**, 6980–6996 (2022).
12. Li, L. *et al.* Accelerating Chemo- and Regioselective Hydrogenation of Alkynes over Bimetallic Nanoparticles in a Metal–Organic Framework. *ACS Catal.* **10**, 7753–7762 (2020).

13. Kuwahara, Y., Kango, H. & Yamashita, H. Pd Nanoparticles and Aminopolymers Confined in Hollow Silica Spheres as Efficient and Reusable Heterogeneous Catalysts for Semihydrogenation of Alkynes. *ACS Catal.* **9**, 1993–2006 (2019).

14. Zhang, J., Xu, W., Xu, L., Shao, Q. & Huang, X. Concavity Tuning of Intermetallic Pd–Pb Nanocubes for Selective Semihydrogenation Catalysis. *Chem. Mater.* **30**, 6338–6345 (2018).

15. Brzozowska, A. *et al.* Highly Chemo- and Stereoselective Transfer Semihydrogenation of Alkynes Catalyzed by a Stable, Well-Defined Manganese(II) Complex. *ACS Catal.* **8**, 4103–4109 (2018).

16. Chen, C., Huang, Y., Zhang, Z., Dong, X.-Q. & Zhang, X. Cobalt-catalyzed (Z)-selective semihydrogenation of alkynes with molecular hydrogen. *Chem. Commun.* **53**, 4612–4615 (2017).

17. Wen, X., Shi, X., Qiao, X., Wu, Z. & Bai, G. Ligand-free nickel-catalyzed semihydrogenation of alkynes with sodium borohydride: a highly efficient and selective process for cis-alkenes under ambient conditions. *Chem. Commun.* **53**, 5372–5375 (2017).

18. Lu, Y. *et al.* Highly Selective Semihydrogenation of Alkynes to Alkenes by Using an Unsupported Nanoporous Palladium Catalyst: No Leaching of Palladium into the Reaction Mixture. *ACS Catal.* **7**, 8296–8303 (2017).

19. Chen, F. *et al.* Selective Semihydrogenation of Alkynes with N-Graphitic-Modified Cobalt Nanoparticles Supported on Silica. *ACS Catal.* **7**, 1526–1532 (2017).

20. Verho, O. *et al.* Application of Pd Nanoparticles Supported on Mesoporous Hollow Silica Nanospheres for the Efficient and Selective Semihydrogenation of Alkynes. *ChemCatChem* **8**, 773–778 (2016).

21. Mitsudome, T. *et al.* Design of Core-Pd/Shell-Ag Nanocomposite Catalyst for Selective Semihydrogenation of Alkynes. *ACS Catal.* **6**, 666–670 (2016).

22. Wagh, Y. S. & Asao, N. Selective Transfer Semihydrogenation of Alkynes with Nanoporous Gold Catalysts. *J. Org. Chem.* **80**, 847–851 (2015).

23. Peng, L. *et al.* The ionic liquid microphase enhances the catalytic activity of Pd nanoparticles supported by a metal–organic framework. *Green Chem.* **17**, 4178–4182 (2015).

24. Niu, W., Gao, Y., Zhang, W., Yan, N. & Lu, X. Pd–Pb Alloy Nanocrystals with Tailored Composition

for Semihydrogenation: Taking Advantage of Catalyst Poisoning. *Angewandte Chemie International Edition* **54**, 8271–8274 (2015).

25. Long, W., Brunelli, N. A., Didas, S. A., Ping, E. W. & Jones, C. W. Aminopolymer–Silica Composite-Supported Pd Catalysts for Selective Hydrogenation of Alkynes. *ACS Catal.* **3**, 1700–1708 (2013).
